# Europeans’ support for refugees of varying background is stable over time

**DOI:** 10.1038/s41586-023-06417-6

**Published:** 2023-08-09

**Authors:** Kirk Bansak, Jens Hainmueller, Dominik Hangartner

**Affiliations:** 1 Department of Political Science, University of California, Berkeley, Berkeley, CA USA; 2 Immigration Policy Lab, Stanford University, Stanford, CA USA; 3 Immigration Policy Lab, ETH Zurich, Zurich, Switzerland; 4 Department of Political Science, Stanford University, Stanford, CA USA; 5 Public Policy Group, ETH Zurich, Zurich, Switzerland

**Keywords:** Politics, Sociology, Society

## Abstract

Protracted global conflicts during the past decade have led to repeated major humanitarian protection crises in Europe. During the height of the Syrian refugee crisis at the end of 2015, Europe hosted around 2.3 million people requesting asylum^1^. Today, the ongoing war in Ukraine has resulted in one of the largest humanitarian emergencies in Europe since World War II, with more than eight million Ukrainians seeking refuge across Europe^2^. Here we explore whether repeated humanitarian crises threaten to exhaust solidarity and whether Europeans welcome Ukrainian asylum seekers over other asylum seekers^3,4^. We conducted repeat conjoint experiments during the 2015–2016 and 2022 refugee crises, asking 33,000 citizens in 15 European countries to evaluate randomly varied profiles of asylum seekers. We find that public preferences for asylum seekers with specific attributes have remained remarkably stable and general support has, if anything, increased slightly over time. Ukrainian asylum seekers were welcomed in 2022, with their demographic, religious and displacement profile having a larger role than their nationality. Yet, this welcome did not come at the expense of support for other marginalized refugee groups, such as Muslim refugees. These findings have implications for our theoretical understanding of the drivers and resilience of public attitudes towards refugees and for policymakers tasked to find effective responses to the enduring stress on the asylum system^5–8^.

## Abstract (web summary)

Surveys conducted in 15 European countries in 2016 and 2022 show stable attitudes towards asylum seekers and refugees with different attributes over this period with a slight increase in support for asylum seekers in general.

## Main

While Europe is still grappling with the aftermath of the Syrian refugee crisis, it again faces millions of refugees, displaced by the Russian invasion of Ukraine. Theory suggests competing predictions for how European attitudes may respond to the ongoing humanitarian protection crisis. One hypothesis is that the European public may become more welcoming towards refugees as Russia’s invasion of Ukraine activates a resurgence in European (and more broadly Western) solidarity efforts. We have indeed seen such solidarity efforts at the government level, with NATO (North Atlantic Treaty Organization) enlargement initiatives and coordinated European sanctions and other measures in response to Russia’s invasion. Alongside these activities, a narrative has emerged of European citizens welcoming Ukrainian refugees with open arms—more open than they have been towards refugee populations in the past^3,9–11^. There has also been scholarly and media discussion about additional reasons for the purported preferential treatment of Ukrainians in Europe. Among the purported reasons are the notions that Ukrainian refugees are predominantly white, well-educated and Christian, that they are a better cultural ‘fit’ than other refugee groups, and that they are more likely to make positive economic contributions^12–16^.

A contrasting perspective holds that Europeans are growing wary of the ever increasing number of refugees. From this view, the Ukrainian crisis could have further enhanced a growing public perception that the ‘boat is full’ and therefore Europe should accept fewer refugees. In light of growing concerns about soaring energy prices and inflation across Europe, we may expect the public to turn away from refugees and towards policies that first and foremost support the local population. Given the rise of populism across Europe^17^, we might also expect greater polarization in attitudes across the ideological spectrum, with support for refugees decreasing particularly among right-wing voters. Furthermore, even if there is increased sympathy towards Ukrainians among some segments of the European public, this might come at the expense of public support for other refugee groups, in particular refugees who are seen as culturally or economically more distant from the European host countries^4^. With its concentration of wealthy host nations and proximity to hotspots of political volatility, Europe is the nucleus of the global asylum regime, and the underlying support and generosity of the European public is a key component. The resilience of that regime may then be threatened by instability at Europe’s doorstep if that instability diverts Europe’s attention away from the rest of the world.

Although there is no one-to-one relationship between public opinion and policies (and while public opinion can itself be influenced by politics and the media), previous scholarship has shown that voter attitudes can have a key role in shaping public policy in democratic countries^18, 19^. A sizable literature has identified drivers of public attitudes towards immigrants^20,21^ and, to a lesser extent, refugees^22,23^. Research during the height of the Syrian refugee crisis uncovered the important roles that economic, humanitarian and religious priorities had in guiding European attitudes towards refugees^5^. Yet, there is a paucity of research that examines how attitudes towards refugees change over time, how they react to repeated inflows of different refugee populations, and whether inflows of refugees from a neighbouring country crowd out support for refugees from more distant regions.

**Extended Data Fig. 1:**
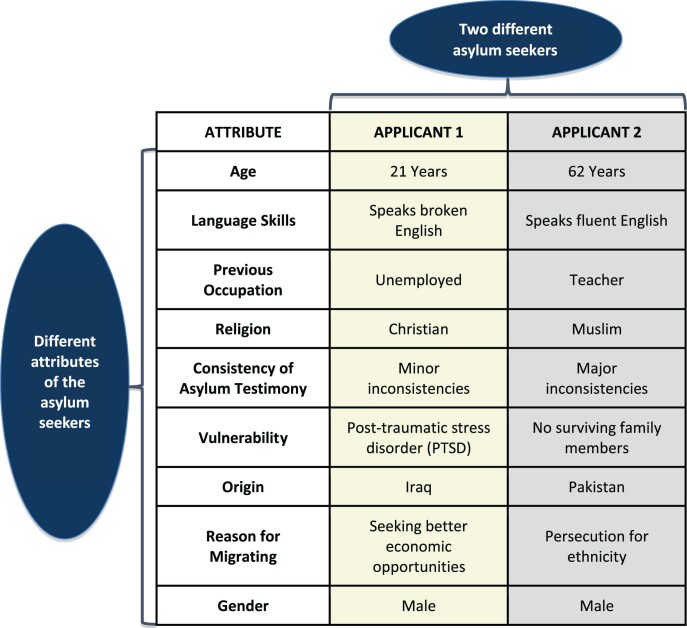
Experimental design. Figure shows an example of a pair of asylum-seeker profiles from the English version of the survey administered to respondents in the United Kingdom. Respondents evaluated five pairs in total. For each profile the specific attribute levels (the last two columns) were fully randomized between and within respondents. Note that the display order of the attributes (rows) was fully randomized between respondents but kept the same for each respondent across the five pairs shown to them.

We leverage survey experimental evidence from 2022 and 2016 to test these competing perspectives. Our goal is to provide a comprehensive empirical assessment of European attitudes towards refugees in light of the current Ukrainian crisis and to examine how attitudes have evolved since the height of the Syrian refugee crisis. When describing our study design and results, we use the terms ‘refugees’ and ‘asylum seekers’ interchangeably throughout, since we cover groups comprising both populations. We conducted a large-scale public opinion survey among approximately 15,000 vote-eligible citizens in 15 European countries. The survey was fielded in May and June 2022, in the midst of the humanitarian emergency in Ukraine. Our research design is based on a conjoint experiment^24,25^ that asks citizens to make choices over randomized profiles of asylum seekers that vary across multiple traits, including country of origin, religion, reasons for migrating and other attributes identified as important by asylum experts and the previous literature (for more information on the attributes and how they were selected for the design, see Extended Data Table 1, Extended Data Fig. 1 and Supplementary Information, ‘Materials and methods’). This conjoint experiment enables us to estimate which specific attributes of asylum seekers generate public support for admission and how this support varies across different groups of respondents and across countries. Crucially, we also conducted an almost identical survey experiment in the midst of the Syrian refugee crisis in 2016 in the same sample of countries, with approximately 18,000 vote-eligible citizens in that first wave^5^. The one difference across the 2016 and 2022 conjoint designs is that we added the ‘War’ level to the attribute on ‘Reason for migrating’ in the 2022 design, given the salience of the Russia–Ukraine war. In the Supplementary Information, we perform analyses of the 2022 survey that validate the comparability of the 2022 and 2016 results. Having fielded these surveys with the same sampling mechanism and almost identical experimental designs enables us to examine how European attitudes towards different refugees have changed over time from one major humanitarian emergency to the next, and whether the share of refugees respondents are willing to admit to their country has increased or decreased.

For each survey wave, we re-weight our sample data using entropy balancing^26^ to match the demographic margins from the populations of each country. For all main analyses, we also provide unweighted results and results based on an alternative set of weights that also account for political ideology, all of which are similar to the main weighted estimates (Supplementary Figs. 1–10). Details about the sample, design and statistical analysis are in Methods and Supplementary Information, Section A. All analyses, unless otherwise noted, were pre-specified in a preregistered analysis plan submitted at https://osf.io/jd8n3/ before the start of the survey.

## Attitudes towards Ukrainian asylum seekers

**Fig. 1:**
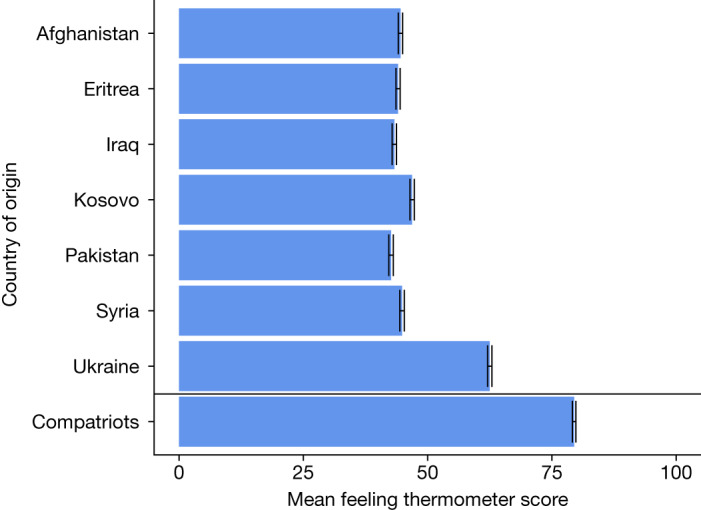
Relative warmth towards asylum seekers and compatriots in 2022. Bars indicate the weighted mean (±95% confidence interval) feeling thermometer score given to asylum seekers from the indicated origin countries as well as compatriots in the 2022 survey wave (*n* = 14,856). The underlying results are presented in Supplementary Table 8.

Figure 1 shows the results of an initial test of the hypothesis of general European warmth towards Ukrainian asylum seekers relative to those from other countries in 2022. Using a ‘feeling thermometer’ question that asked respondents to rate their warmth towards particular groups from 0 to 100, we find that general attitudes towards Ukrainian asylum seekers are markedly more positive compared to all other asylum seeker groups. The mean feeling thermometer score for Ukrainian asylum seekers is 62.5, which is higher than and statistically different from the mean feeling thermometer score for each of the other asylum seeker groups, which range from 42.7 to 46.9 (|*t|* = 50.08–62.92; maximum *P* *<* 0.00001; two-sided *t*-tests; *n* = 14,856). The mean score for Ukrainian asylum seekers is approximately equidistant between those for the non-Ukrainian asylum seekers and that for compatriots (79.5).

## Stability in preferred asylum seeker traits

**Fig. 2:**
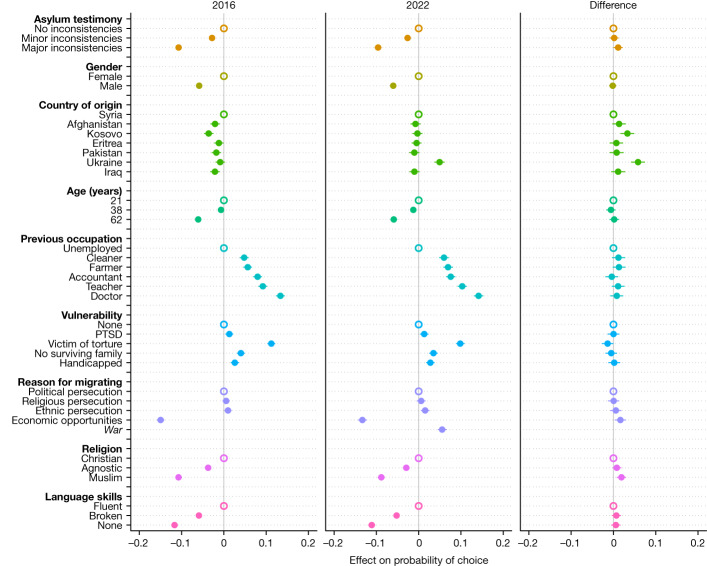
Effects of asylum seeker attributes on the probability of respondent choice in 2016 and 2022. Dots with horizontal lines indicate point estimates with cluster-robust 95% confidence intervals from linear (weighted) least-squares regression. The unfilled dots on the zero line denote the reference category for each asylum seeker attribute. The results from 2016 were first presented in ref. ^5^. Attributes that were only included in 2022 are shown in italics. *n* = 178,740 profiles evaluated for 2016 and *n* = 148,460 profiles evaluated for 2022. The underlying regression results are presented in Supplementary Table 9. PTSD, post-traumatic stress disorder.

To unpack this support, we leverage the data from the conjoint experiment. Figure 2 shows the estimated effects of the asylum seeker attributes on support, pooling across all respondents in the 2022 and the 2016 waves of the survey. These results are based on a forced choice outcome, which denotes whether a profile was preferred or not in a randomly generated pair of profiles. The Supplementary Information provides results for analogous analyses that use a rating outcome (with a scale of 1 to 7 that respondents used to rate profiles individually) and a dichotomized version of the rating variable, and the findings are similar (Supplementary Figs. 11 and 12). Figure 2 reveals several important findings.

First, we find that Europeans’ patterns of preferences regarding the desirable characteristics and traits of asylum seekers have remained remarkably stable between the height of the Syrian crisis in 2015–2016 and the height of the Ukrainian crisis in 2022 (Fig. 2). Inconsistencies in asylum testimony, gender, age, previous occupation, special vulnerabilities, reason for migrating, religion and language skills each have similar effects on the probability of being supported for admission in both survey waves. Major inconsistencies have a negative 9.6–10.7 percentage point effect relative to no inconsistencies (|*t*| = 23.42–28.38; maximum *P* *<* 0.00001 for effects in both survey waves based on two-sided *t*-tests of estimates from linear least-squares regressions; *n* = 178,740 in 2016; *n* = 148,460 in 2022). Being male has a negative 5.8–6.0 percentage point effect relative to being female (|*t*| = 18.72–19.64; maximum *P* *<* 0.00001). Being 62 years old has a negative 5.9–6.1 percentage point effect relative to being 21 years old (|*t*| = 14.39–15.54; maximum *P* *<* 0.00001). Having been previously employed has a positive 4.8–14.2 percentage point effect and having been employed in a high-skilled profession (accountant, teacher or doctor) has a positive 7.6–14.2 percentage point effect relative to having been unemployed (|*t*| = 9.06–25.05; maximum *P* *<* 0.00001). Being a victim of torture has a positive 9.8–11.2 percentage point effect relative to having no special vulnerabilities (|*t*| = 18.95–23.65; maximum *P* *<* 0.00001). Being Muslim has a negative 8.8–10.7 percentage point effect relative to being Christian (|*t*| = 21.39–28.04; maximum *P* *<* 0.00001). Having no host country language skills has a negative 11.1–11.7 percentage point effect relative to being fluent (|*t*| = 27.19–30.08; maximum *P* *<* 0.00001). Finally, there is a negative effect of 13.3–18.9 percentage points for asylum seekers who are migrating for economic reasons rather than fleeing persecution or war (with economic reasons set as the reference category, |*t*| = 25.50–36.26; maximum *P* *<* 0.00001).

**Extended Data Fig. 2:**
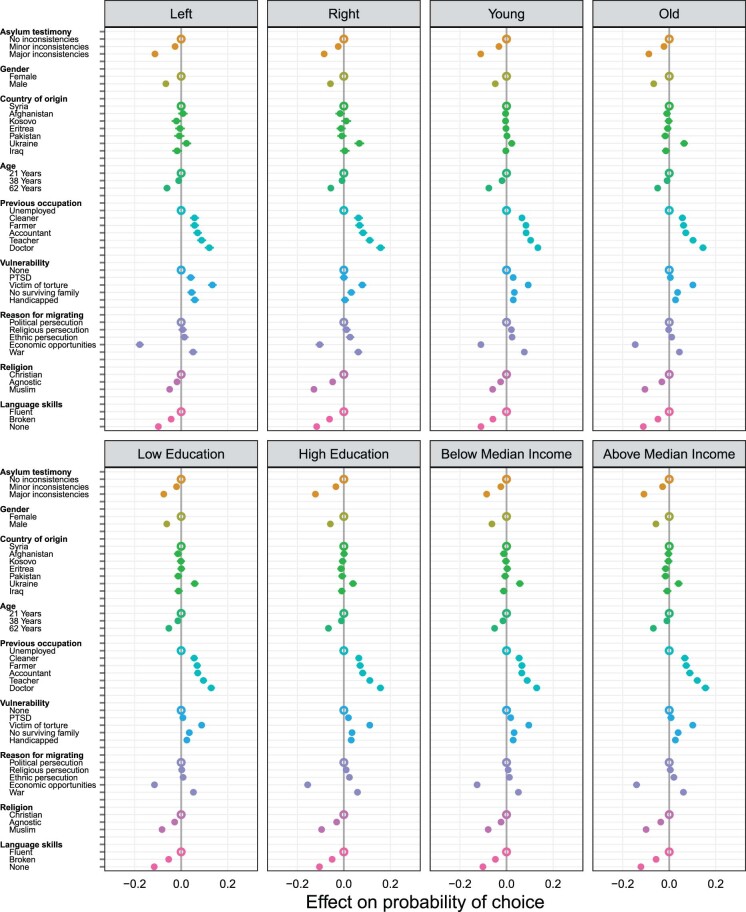
Effects of asylum-seeker attributes on the probability of respondent choice, across subgroups of respondents, results from 2022. .

**Extended Data Fig. 3:**
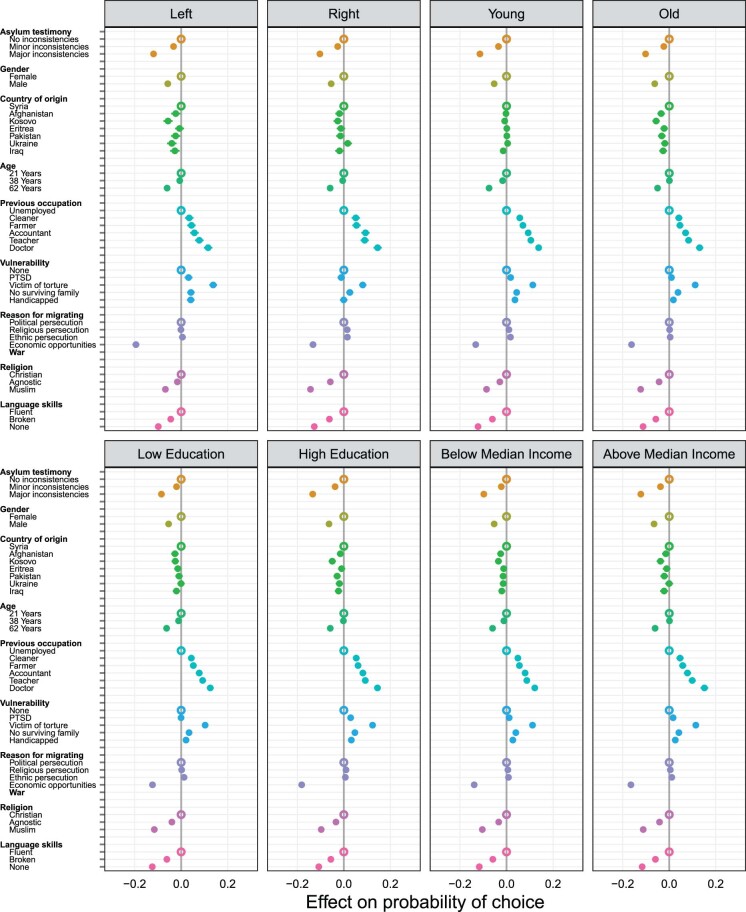
Effects of asylum-seeker attributes on the probability of respondent choice, across subgroups of respondents, results from 2016. .

**Extended Data Fig. 4:**
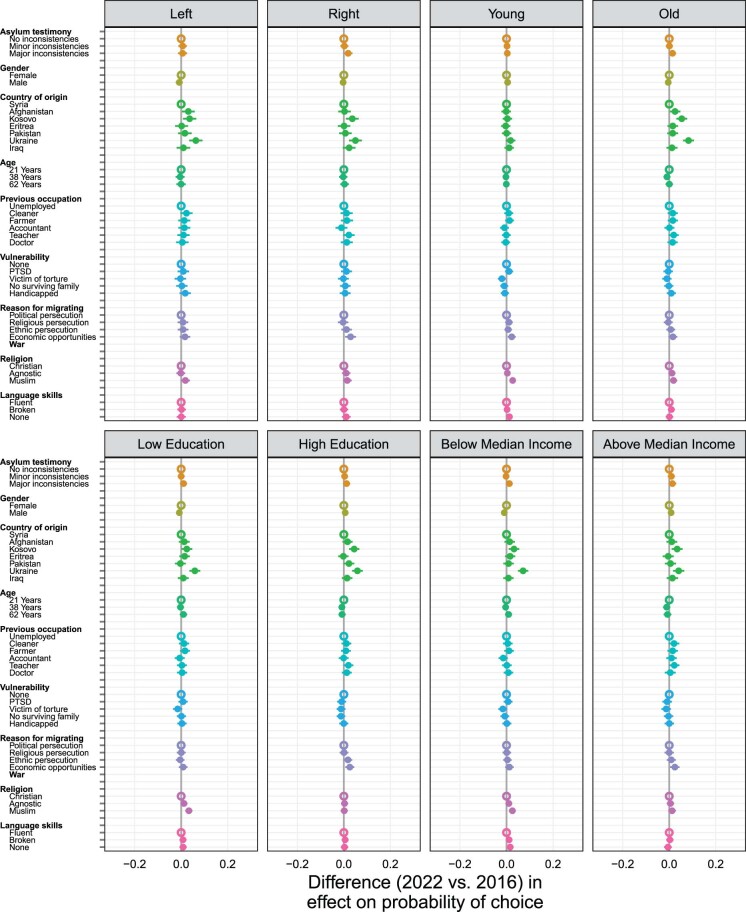
Difference (2022 vs. 2016) in effects of asylum-seeker attributes on the probability of respondent choice, across subgroups of respondents. .

Furthermore, the differences in the magnitudes of the effects of all of these characteristics (excluding country of origin) between the 2016 and the 2022 survey waves are minimal (Fig. 2). Of all the differences, only 4 out of 21 are statistically significant at *P* *<* 0.05 (|*t*| = 1.96–3.37 for the significant differences; |*t*| = 0.04–1.70 for the insignificant differences; two-sided *t*-tests; *n* = 178,740 in 2016; *n* = 148,460 in 2022), and the largest difference in absolute value is 0.019 (1.9 percentage points). Further, the two one-sided *t*-test procedure (TOST equivalence test) with equivalence bounds of *−*0.03 and 0.03—that is, 3 percentage point differences—yields rejection for all differences at *P* *<* 0.05 (*t* = 2.30–8.71 for one-sided *t*-tests against lower bound; *t* = −7.24 to −1.96 for one-sided *t*-tests against upper bound) (and rejection for all differences at *P* *<* 0.0005 for equivalence bounds of *−*0.04 and 0.04; *t* = 3.73 to 10.48 for one-sided *t*-tests against lower bound; *t* = −9.52 to −3.45 for one-sided *t*-tests against upper bound). This shows that rather than being sensitive to the repeated crises and protracted trends, the structure of European public attitudes towards asylum seekers is remarkably stable over time. These findings are also similar when we stratify the analysis by voters’ political ideology, age, education and income, or by the host country (Extended Data Figs. 2–4 and Supplementary Figs. 14–17).

A second major takeaway from the results in Fig. 2 relates to the role of country of origin. There is a statistically significant positive Ukraine effect in 2022, compared with a much smaller effect in 2016, suggesting that there have been some minor changes to European preferences in the face of the war in Ukraine. If forced to choose, Europeans in 2022 have a 5.5 percentage point higher probability of choosing a Ukrainian versus a non-Ukrainian asylum seeker (|*t*| = 11.71; *P* *<* 0.00001; two-sided *t*-test; *n* = 148,460), whereas in 2016 there is only a 0.9 percentage point effect (|*t*| = 2.13; *P* *<* 0.05; two-sided *t*-test; *n* = 178,740), where these percentage point effects are the estimates if all the other origins are pooled as the reference category. The 4.6 percentage point difference between these two is statistically significant (|*t*| = 7.18; *P* *<* 0.00001; two-sided *t*-test; *n* = 178,740 in 2016; *n* = 148,460 in 2022), and a TOST equivalence test of the 0.9 percentage point effect in 2016 with equivalence bounds of *−*3 and 3 percentage points rejects at *P* *<* 0.00001 (*t* = 9.03 for one-sided *t*-test against lower bound; *t* = −4.76 for one-sided *t*-test against upper bound). Nonetheless, the importance of Ukrainian origin is limited compared with the collective effects of the other attributes in 2022. For instance, in contrast to the 5.5 percentage point Ukraine effect, Europeans in 2022 expressed an 8.8 percentage point preference for Christians versus Muslims (|*t*| = 21.39; *P* *<* 0.00001; two-sided *t*-test; *n* = 148,460), a 7.6–14.2 percentage point preference for skilled professionals (accountants, teachers and doctors) relative to unemployed individuals (|*t*| = 13.70–24.88; maximum *P* *<* 0.00001) and a 6.0 percentage point preference for females versus males (|*t*| = 18.72; *P* *<* 0.00001).

In sum, European preferences with respect to the desirable features of asylum seekers have changed little across the Syrian and the Ukrainian humanitarian crises. It is in fact this resilience in preferences (rather than a sudden reaction to the state of affairs) that appears to be the primary driver of current support for Ukrainian refugees in Europe. Indeed, large-scale surveys carried out by the United Nations High Commissioner For Refugees (UNHCR) show that compared with refugees from other countries of origin, Ukrainian refugees are predominantly female, younger and Christian, they are more highly educated, and they are more likely to have worked in middle- and high-skilled occupations^15^. In light of this, our results suggest that the predominant source of support for Ukrainian asylum seekers in 2022 is the strong and longstanding preferences for specific traits that Ukrainian asylum seekers happen to possess. In other words, the main driver underlying the sympathy for Ukrainians is not a new attitudinal phenomenon, but rather a consequence of the socio-demographic composition and displacement profile of Ukrainian refugees.

As a more minor influence, we also find evidence for European solidarity as a new source of support for asylum seekers who are Ukrainian per se. Indeed, the small positive Ukraine effect that we see in the 2022 conjoint results is 2.7 percentage points higher among Europeans who possess higher sentiments of European solidarity compared with Europeans with lower sentiments (specifically, a 7.5 percentage point effect versus a 4.7 percentage point effect, the 2.7 percentage point difference of which is statistically significant at *P* *<* 0.01; |*t*| = 2.61; two-sided *t*-test; *n* = 148,460). Supplementary Information, ‘Additional analyses’ and Supplementary Fig. 18 provide further details, including a supplementary analysis that suggests that this effect may indeed be causally moderated^27^ by (rather than simply correlated with) European solidarity attitudes (Supplementary Fig. 19)—a result that resonates with the conscience collective discussed in ref. ^16^. Furthermore, we find that the Ukraine effect is specific to Ukraine during the invasion: we did not observe an effect of this magnitude in 2016 as described above, and it is also not driven by a general preference for all European asylum seekers, as we find no differential treatment of asylum seekers from Kosovo relative to those from Syria, Afghanistan, Eritrea, Iraq and Pakistan in 2022. For this latter comparison, we set the reference category to Kosovo and estimate the effects for Syria, Afghanistan, Eritrea, Iraq and Pakistan—none of the effects are statistically significant at *P* *<* 0.05 (|*t*| = 0.34–1.20; two-sided *t*-tests; *n* = 148,460), and TOST equivalence tests with equivalence bounds of *−*0.03 and 0.03 (3 percentage point bounds) yield rejection for all effects at *P* *<* 0.0001 (*t* = 3.74–5.43 for one-sided *t*-tests against lower bound; *t* = −6.18 to −4.46 for one-sided *t*-tests against upper bound).

## Increase in support for asylum seekers overall

The previous findings suggest that attitudes towards the types of asylum seekers who are preferred by Europeans are remarkably stable across the two major crises. We next consider whether general support for the admission of asylum seekers has increased or decreased over time. To assess this, we analyse which asylum seeker profiles in our conjoint experiment gained support based on a dichotomized version of a rating variable that distinguishes between accepted and rejected profiles, rather than focusing on forced choices between profiles.

**Fig. 3:**
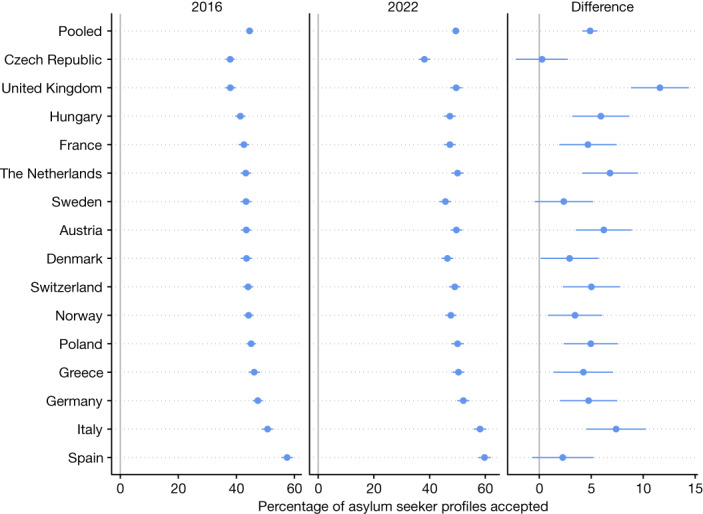
The percentage of asylum seeker profiles accepted in 2016 and 2022 surveys. For comparability, profiles that were randomly assigned the ‘War’ level for the ‘Reason for migrating’ attribute in 2022 are omitted. Data are mean ± 95% confidence interval. The 2016 data were first presented in ref. ^5^. *n* = 178,740 profiles evaluated for 2016 and *n* = 118,807 profiles evaluated for 2022. The underlying results are presented in Supplementary Table 10.

**Extended Data Fig. 5:**
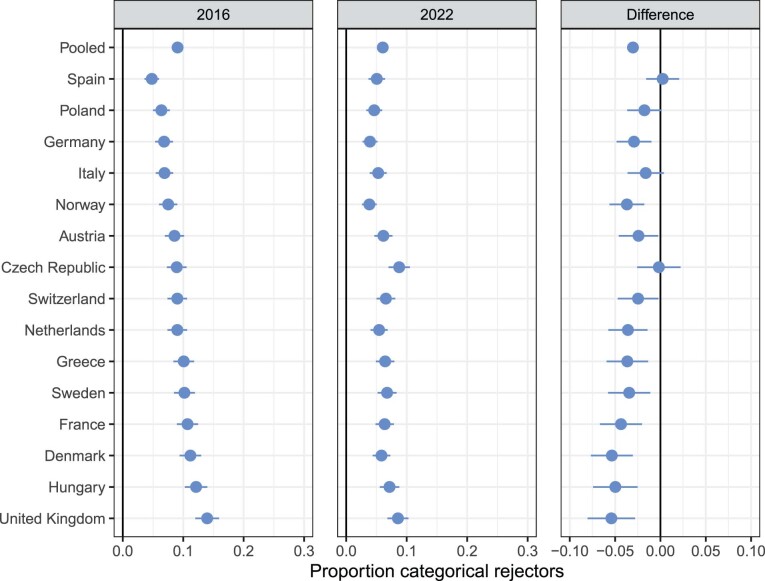
Proportion of respondents who are categorical rejecters, results from 2016, 2022, and difference. .

**Extended Data Fig. 6:**
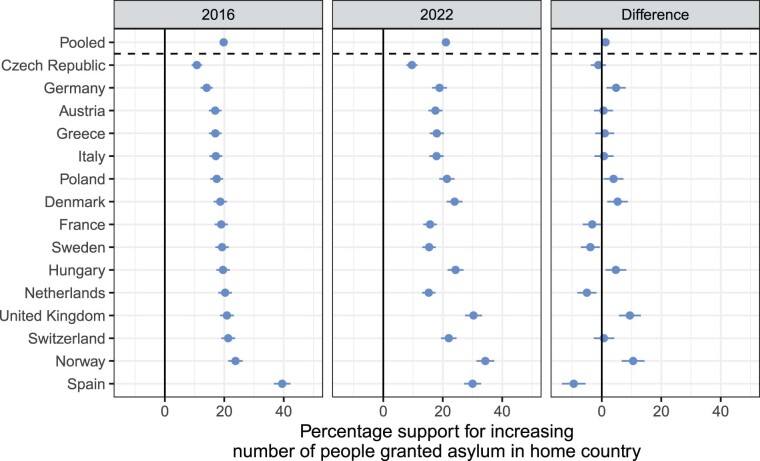
Support for increasing the number of people granted asylum in home country, results from 2016, 2022, and difference. .

**Extended Data Fig. 7:**
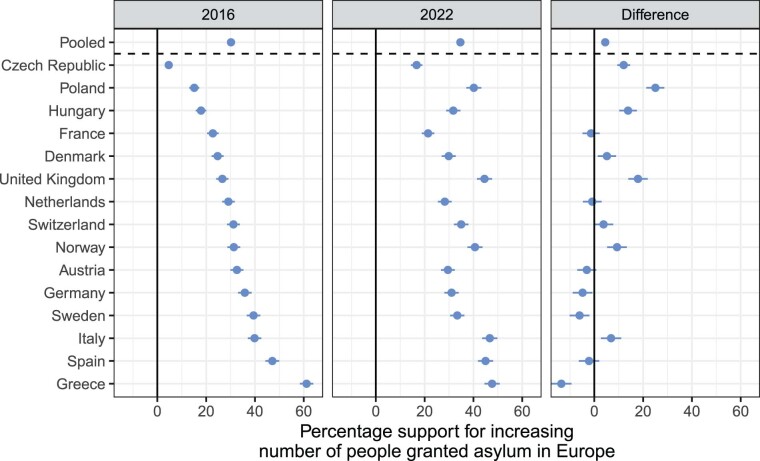
Support for increasing the number of people granted asylum in Europe, results from 2016, 2022, and difference. .

Figure 3 plots the fraction of asylum seeker profiles that respondents are willing to accept for each country in 2022 and 2016, as well as the difference between the two waves. For fairer comparison across the 2022 and 2016 results, profiles that were randomly assigned the ‘War’ level for the ‘Reason for migrating’ attribute in the 2022 data are omitted from the analysis (including these profiles would lead to a minor boost in the increased support in 2022, consistent with the results when omitting the profiles). In stark contrast to the idea that Europeans have become more wary of admitting refugees in light of repeated major crises, we find that support for asylum seekers today is, if anything, slightly higher than six years ago at the height of the Syrian refugee crisis. As shown in the right panel of Fig. 3, the percentage of profiles accepted pooling over all countries increased by 4.9 percentage points from 2016 to 2022 (|*t*| = 13.41; *P* *<* 0.00001; two-sided *t*-test; *n* = 178,740 profiles evaluated in 2016; *n* = 118,807 in 2022). Furthermore, for the majority of countries individually there was a statistically significant increase (*P* *<* 0.05 in 12 countries; |*t*| = 2.06–8.22 in these countries; two-sided *t*-tests; *n* = 11,280–12,020 profiles evaluated in each of these countries in 2016; *n* = 7,669–8,060 in 2022), and there was not a single country with a negative point estimate. In other words, in Europeans’ minds the boat is not full (or at least not any fuller than in 2016). We further supplement this evidence with two additional analyses. First, we categorize individual respondents as ‘categorical rejecters’ if they gave a rating of lower than 4 (out of 7) to all of the profiles they viewed, and compute the proportion of respondents who are categorical rejecters (Extended Data Fig. 5). Second, we analyse the results from simple survey questions asking whether the granting of asylum should be increased at home and in Europe (Extended Data Figs. 6 and 7). For both analyses, we find similar results: limited but statistically significant increases in support for granting asylum (1.3 percentage point increase for asylum at home and 4.5 percentage point increase for asylum in Europe), along with a 3.0 percentage point decrease in the prevalence of categorical rejecters, in 2022 versus 2016, pooling across all countries (|*t*| = 2.83–10.43; *P* *<* 0.005 for each contrast; two-sided *t*-tests; *n* = 17,883 in 2016; *n* = 14,856 in 2022).

**Fig. 4:**
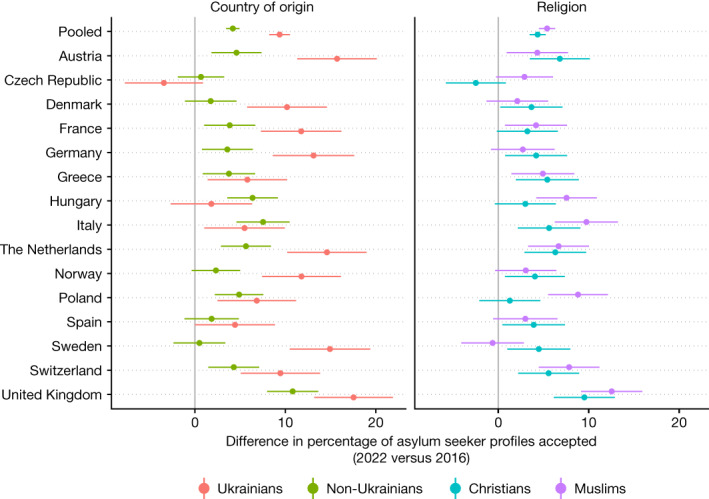
Difference in the percentage of asylum seeker profiles accepted, by country of origin and religion of asylum seeker, in 2022 versus 2016. For comparability, profiles that were randomly assigned the ‘War’ level for the ‘Reason for migrating’ attribute in 2022 are omitted. Data are mean ± 95% confidence interval. The underlying results are presented in Supplementary Table 11.

There could be several possible reasons for this resilience in support for refugees overall. One possibility is that the increased support for asylum seekers today is driven primarily by the Russian invasion of Ukraine and European solidarity leading to a one-off increase in generosity towards Ukrainians, but this may come at the expense of support for asylum seekers from other countries. To test this, we plot the difference in the fraction accepted separately for Ukrainian and non-Ukrainian asylum seeker profiles in our conjoint experiments (this test was not pre-specified) (Fig. 4). The findings strongly contradict the idea that the increase in general support is limited to Ukrainian refugees. Indeed, in most countries there is a statistically significant increase in the percentage of accepted non-Ukrainian asylum seeker profiles in 2022 versus 2016 (*P* *<* 0.05 in 10 countries; |*t*| = 2.50–7.51 in these countries; two-sided *t*-tests; *n* = 9,742–10,306 non-Ukrainian profiles evaluated in each of these countries in 2016; *n* = 6,533–6,913 in 2022), and there is no country where the point estimate of the difference is negative. This suggests that the increased support for refugees extends to other, non-Ukrainian groups of asylum seekers and that there is no evidence of substitution effects. This is also the case when focusing on religion, another politically salient dimension. In stark contrast to the prediction that other refugee groups would face decreased support, we find that the percentage of accepted Muslim profiles has significantly increased in the majority of countries (*P* *<* 0.05 in 9 countries; |*t*| = 2.39–7.27 in these countries; two-sided *t*-tests; *n* = 3,920–4,066 Muslim profiles evaluated in each of these countries in 2016; *n* = 2,643–2,738 in 2022), and the only point estimate that is negative (a 0.62 percentage point decrease in Sweden) is not statistically significant at *P* *<* 0.05 (|*t*| = 0.35; *P* = 0.72; two-sided *t*-test; *n* = 3,878 in 2016; *n* = 2,641 in 2022). Again, there is no evidence of substitution of acceptance towards Ukrainians and Christians for acceptance towards other asylum seekers.

**Fig. 5:**
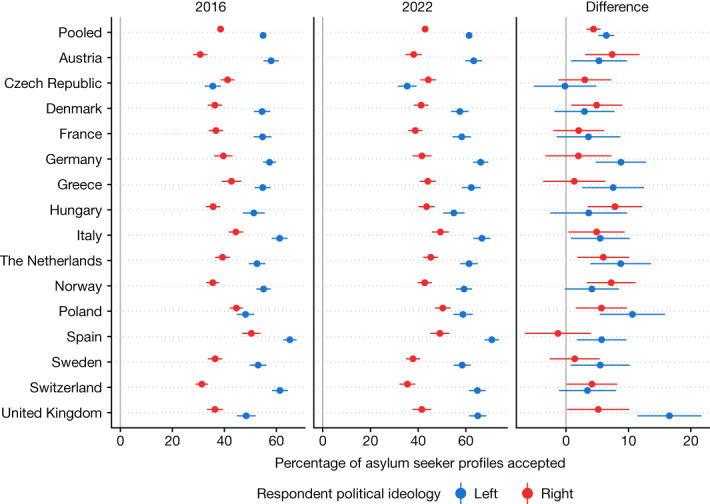
The percentage of asylum seeker profiles accepted, by political ideology of respondent, in 2016 and 2022. For comparability, profiles that were randomly assigned the ‘War’ level for the ‘Reason for migrating’ attribute in 2022 are omitted. Data are mean ± 95% confidence interval. The underlying results are presented in Supplementary Tables 12 and 13 display the underlying results.

Another possibility is that the increased support for asylum seekers is driven by increased political polarization. To examine this, Fig. 5 breaks down the support and the change in support by political ideology (this test was not pre-specified). The past decade has seen the rise of right-wing parties, populism and polarization^28^. Such political contention has included and often revolved around immigration issues, and indeed right-wing voters are in general less likely to support refugees than left-wing voters (Fig. 5): pooling across all countries in 2022, the percentage of profiles accepted is 61.3 for left-wing voters and 42.9 for right-wing voters, an 18.40 percentage point difference that is statistically significant (|*t*| = 28.10; *P* *<* 0.00001; two-sided *t*-test; *n* = 37,731 profiles evaluated by left-wing voters; *n* = 40,979 for right-wing voters). However, we also find increased support for asylum seekers in 2022 relative to 2016 that manifests among both right-wing and left-wing voters: pooling across countries, there is a 4.4 percentage point increase in support among right-wing voters and a 6.5 percentage point increase among left-wing voters, both of which are statistically significant increases (|*t*| = 7.58–10.11; maximum *P* *<* 0.00001; two-sided *t*-tests; *n* = 57,450 profiles evaluated by left-wing voters in 2016; *n* = 61,440 for right-wing voters in 2016; *n* = 37,731 for left-wing voters in 2022; *n* = 40,979 for right-wing voters in 2022).

In sum, these results demonstrate that support for asylum seekers has increased despite the repeated crises, and that this increase in support extends broadly to all refugees, not just to Ukrainians, and is also shared among voters on both ends of the political spectrum.

## Discussion

Our repeated conjoint experiments covering 33,000 respondents in 15 European countries during the 2015–2016 and 2022 refugee crises find that public preferences for specific classes of asylum seekers have remained remarkably stable and general support has, if anything, slightly increased over time. This study has two main limitations. There are, as with all surveys, potential concerns about external validity related to the measurement instrument and the sample. Validation tests have shown that the immigrant preferences elicited by our measurement instrument, the paired conjoint design, have high external validity and replicate respondents’ real-world voting behaviour^29^. Another validation study based on eye-tracking methodology found that the statistical importance measures inferred from respondents’ stated choice in conjoint experiments are correlated with attribute importance measures based on their eye movement^30^. In addition, we replicate our analysis using a rating outcome as well as a dichotomized version of the rating outcome and find similar results (Supplementary Figs.  11 and 12). With regard to representativeness of the sample, we conduct two robustness tests to probe the stability of our results. First, we find that unweighted results are similar to the estimates weighted for each country’s age, gender and education distributions (1–5). We also conduct our analyses using a second set of weights that, in addition, take into account each country’s political ideological distribution—measured on a standard 0–10 left–right ideology scale. Again, the results are similar to the original weighted estimates (Supplementary Figs. 6–10). In addition, we note that the same sampling mechanism was employed for both the 2016 and 2022 waves, limiting concerns about non-comparable samples.

A second limitation of our study is that our data were only collected during the 2015–2016 and 2022 humanitarian protection crises, and not at any points in between. Thus, we cannot be certain about any fluctuations in public preferences and support that may have taken place across the entire period. Nevertheless, the considerable consistency that we see across our two waves in preferences over the various asylum seeker attributes—that is, the fact that the effects in Fig. 2 are virtually identical across 2016 and 2022 with the exception of country of origin—is highly suggestive of broader stability and a limited likelihood of significant fluctuations in that regard. In addition, the fact that overall support for asylum seekers has remained robust in 2022 relative to 2016 in spite of repeated crises—and in spite of continuous strain on the asylum system over the entire period—indicates an underlying longer-term durability in public support, even if there were fluctuations between 2016 and 2022. Furthermore, we conducted supplementary analyses using data from the biennial European Social Survey (ESS) to examine attitudes over a longer time period from 2002 to 2020 (see Supplementary Information, ‘Additional analyses’ for more details; the ESS analysis was not preregistered). Although the ESS does not regularly ask about refugees in particular, it does measure support for two related types of immigration—from poorer countries outside Europe and of people of a different race or ethnic group than the majority group. We find that attitudes towards both types of immigration were fairly stable across the past two decades and there may have been a small increase in support in the most recent ESS waves (Supplementary Figs. 27–30). In addition, in our own surveys, we find a close and stable correspondence between support for immigration in general and support for asylum seekers (Supplementary Table 14), suggesting that these attitudes are linked. Overall, the stability of attitudes to immigration evidenced in the ESS is highly consistent with the stability in the asylum preferences we find comparing 2016 and 2022, and further corroborates the pattern of an underlying longer-term resilience in public support for refugees.

Our results have important implications for theory and policy. For theory, these results suggest that attitudes towards asylum seekers are more stable than previously thought and that they can become increasingly generous despite repeated humanitarian emergencies and a rise in populism. These findings are consistent with research that has found a similar stability in general attitudes towards immigrants^7,8^ and political preferences more broadly^31^. Our results also speak against the prominent claim that the emergence of new and culturally less distant immigrant groups will lead to a backlash of public preferences and decreased support for other minority groups^4^. Our data provide little evidence that public support for different refugee groups is characterized by substitutive or zero-sum reasoning. By contrast, we find that support for all refugees remains relatively generous when it arguably matters most: during times of crisis when refugee arrivals peak.

For policy, the remarkable stability in preferences suggests that public attitudes may be less malleable than previously thought and there seems to be a strong and durable consensus over the specific types of asylum seekers that are preferred^5,32^. Yet, our results suggest that policymakers should be able to leverage generosity towards Ukrainians in light of the current crisis and that there is little evidence of asylum fatigue getting in the way of efforts to continue to provide protection to people in need. In this regard, it appears that the regional priority to protect Ukrainians, at least in the eyes of the European public, does not crowd out the global outlook of the European asylum system and the efforts to protect other refugee groups.

## Methods

### Data collection, sample and sample weights

Our online surveys in 2022 and 2016 were fielded by the survey research firm Respondi and its local partners in the same set of 15 European countries: Austria, the Czech Republic, Denmark, France, Germany, Greece, Hungary, Italy, the Netherlands, Norway, Poland, Spain, Sweden, Switzerland and the United Kingdom. The number of respondents per country was about 1,200 (totalling *n* = 18,030) in the 2016 wave and about 1,000 per country in the 2022 wave (totalling *n* = 14,976); see Supplementary Table 1 for details. For each survey wave, we re-weight our sample to match the age, gender and educational attainment margins for each country. In addition, the Supplementary Information provides unweighted estimates and weighted estimates that also account for the distribution of political ideology in the country. Our 2016 survey was conducted according to the University of Zurich’s policy for human subjects research and approved by Stanford University’s Institutional Review Board (protocol ID 34881). Our 2022 survey was approved by Stanford University’s Institutional Review Board (protocol ID 34881) and ETH Zurich’s Ethics Committee (protocol IRB00007709).

### Experimental design

To measure respondents’ support for specific types of asylum seekers, we deploy a fully randomized paired profiles conjoint design. Each respondent was presented with five pairs of profiles of hypothetical asylum seekers displayed side by side (Extended Data Fig. 1). The profiles described hypothetical asylum seekers with nine attributes, including the asylum seeker’s age, proficiency in the host country language, previous occupation, religion, consistency of the asylum testimony, special vulnerabilities, country of origin, reason for migrating and gender. The sole difference between the conjoint design in the 2016 and the 2022 wave is that we added a ‘War’ level for the attribute ‘Reason for migrating’ to the 2022 wave.

### Outcomes

We elicited two outcome measures for each pair of asylum seeker profiles shown. First, we measured how supportive respondents would be of allowing the hypothetical asylum seeker to stay in their country. For this rating outcome variable, we asked respondents to rate each profile separately on a scale from 1 (definitely send the applicant back) to 7 (definitely allow the applicant to stay). Second, for our forced choice outcome variable, we asked respondents for each pair to pick the one asylum seeker that they would prefer to be allowed to stay in the country. This forced choice outcome is coded as 1 for the preferred profiles and 0 for the rejected profiles. Both outcomes generate similar results and we focus on the latter for our main analysis in Fig. 2.

### Statistical analysis

Each of the approximately 33,000 respondents across the 2 waves evaluated 5 pairs of profiles, resulting in a total of approximately 330,000 asylum seeker profiles. Since the attribute values were randomly assigned across respondents and profiles, we can estimate the average marginal component effects, which measure the average causal effect of each attribute on respondents’ acceptance of an asylum seeker. We use linear (weighted) least-squares regression to regress the rating and choice outcomes on sets of indicator variables that measure the values of each attribute while omitting one level of each attribute as the reference category. To account for correlation in outcomes within the same respondent, we cluster standard errors by respondent.

All analyses in the main text, except those in Figs. 4 and 5, were pre-specified in a preregistered analysis plan submitted at https://osf.io/jd8n3/ before the start of the survey. Supplementary Information, Section A provides further details on sample, design, questionnaire, statistical analysis and deviations from the preregistered analysis plan.

### Reporting summary

Further information on research design is available in the Nature Portfolio Reporting Summary linked to this article.

## Online content

Any methods, additional references, Nature Portfolio reporting summaries, source data, extended data, supplementary information, acknowledgements, peer review information; details of author contributions and competing interests; and statements of data and code availability are available at 10.1038/s41586-023-06417-6.

### Supplementary information

Supplementary Information This file contains supplementary methods that provide further details on the survey questionnaire, sampling, weighting and statistical analysis, and Supplementary Tables 1–14 and Supplementary Figs. 1–33, which extend the main analysis.

Reporting Summary

## Figures and Tables

**Extended Data Table 1:**
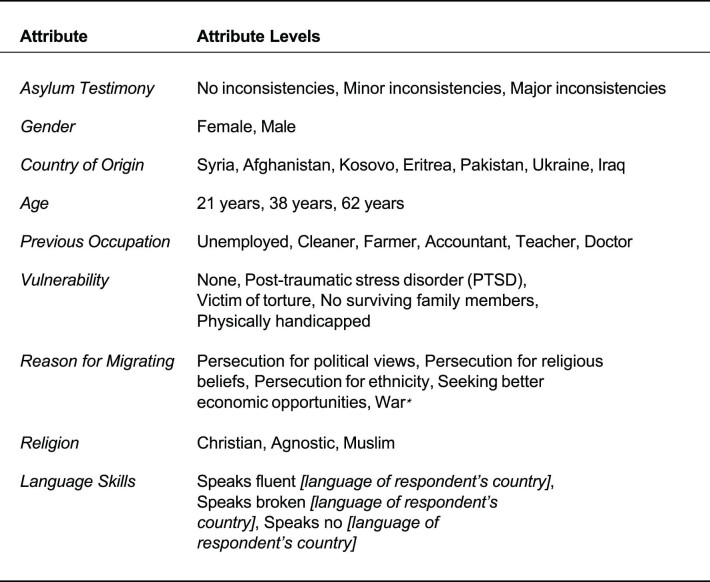
The table shows the attributes and attribute levels that are used in the conjoint experiment to construct the asylum-seeker profiles.

The list is based on the exact wording shown to respondents in the United Kingdom. Note: ^⋆^ For *Reason for Migrating* the attribute level “War” was added in the 2022 wave of the survey to better capture the Ukrainian crisis. We included “Seeking better economic opportunities” as one of the values for the *Reason for Migrating* attribute because the group of asylum seekers who come to Europe is heterogeneous and, in fact, includes people who migrate in search of economic opportunities. These types of asylum seekers are often referred to as economic migrants, and from a purely legal perspective, a person who migrates for purely economic reasons does not meet the requirements to be granted refugee status under international law, which requires that the person faces persecution because of their race, religion, nationality, or political opinion. Our conjoint experiment was designed to capture these various motivations of asylum seekers, rather than limiting the profiles to the narrower group of asylum seekers that meet the requirements of refugee status under the 1951 Refugee Convention, in order to investigate the extent to which public preferences are consistent with international law.

## Data Availability

All data from our two-wave survey required to replicate our analyses are available at the publicly accessible Harvard Dataverse: 10.7910/DVN/FTL1MM. Researchers interested in replicating the supplementary analysis based on the ESS can download the data from https://www.europeansocialsurvey.org/data. All analyses in the main text, except those in Figs. 4 and 5, were pre-specified in a preregistered analysis plan submitted at https://osf.io/jd8n3/ before the start of the survey. Supplementary Information, Section A discusses deviations from the preregistered analysis plan.

## Code availability

Code to replicate all analyses presented here is available at the publicly accessible Harvard Dataverse: 10.7910/DVN/FTL1MM.
