# Supplementary material for: Europeans’ support for refugees of varying background is stable over time
Source: Nature. 2023 Aug 9;620(7975):849–54. doi: 10.1038/s41586-023-06417-6 (PMC10447233; doi:10.1038/s41586-023-06417-6)
Supplement: Supplementary file 1 — This file contains supplementary methods that provide further details on the survey questionnaire, sampling, weighting and statistical analysis, and Supplementary Tables 1–14 and Supplementary Figs. 1–33, which extend the main analysis. [file 41586_2023_6417_MOESM1_ESM.pdf]

nature portfolio

https://doi.org/10.1038/s41586-023-06417-6

Supplementary information

Europeans' support for refugees of varying background is stable over time

In the format provided by the authors and unedited

Supplementary Information:

Europeans' support for refugees of varying background is stable over time

Kirk Bansak,[1,4] Jens Hainmueller,[2,4] Dominik Hangartner[3,4]

[1] Department of Political Science, University of California, Berkeley, 210 Social Sciences Building, Berkeley, CA 94720-1950.

[2] Department of Political Science, 616 Serra Street Encina Hall West, Room 100, Stanford, CA 94305-6044.

[3] Public Policy Group, ETH Zurich, Leonhardshalde 21, 8092 Zurich, Switzerland.

[4] Immigration Policy Lab, Stanford University, Stanford, CA 94305 and ETH Zurich, 8092 Zurich, Switzerland.

Contents

A Supplementary Materials and Methods **ii**

B Supplementary Text **xx**

C Supplementary Figures and Tables **xxii**

A Supplementary Materials and Methods

Sample Our surveys in 2022 and 2016 were fielded in the same set of 15 European countries including Austria, the Czech Republic, Denmark, France, Germany, Greece, Hungary, Italy, the Netherlands, Norway, Poland, Spain, Sweden, Switzerland, and the United Kingdom. These countries were originally selected because they all belong to the Common European Asylum System (CEAS) that sets common legal standards for the regulations of the asylum system in the European Union (EU).[1] In addition, the countries cover a wide variety of characteristics including larger and smaller countries, border, coastal, and non-coastal border countries, and countries with varying numbers of asylums seekers.

The first survey wave was conducted between late February and early March 2016, the second wave between mid May and early June 2022. For each country and survey wave, the survey firm Respondi and its local partners sampled eligible voters from their online panel. Respondi recruits new panelists for its panel from the general population, mostly through online channels and to a lesser extent through computer-assisted telephone interviews (CATI). After completing the enrollment interview, Respondi invites panelists to participate in several surveys (like ours), for which they are compensated. The same sampling mechanism was used for both our 2016 and 2022 survey waves. In addition to Respondi's standard recruitment processes, the recruitment of panelists for both waves of our survey also employed age and gender quotas to roughly match the population margins for each of the countries in our study. Post-stratification weights were constructed to account for remaining imbalances, as explained below.

The number of respondents per country was about 1,200 (totaling $N = 18,030$) in the 2016 wave and about 1,000 in the 2022 wave (totaling $N = 14,976$); see Table S1 for details. The median length of interview (LOI) was 20.53 minutes in the 2016 and 16.43 minutes in the 2022 survey.

[1]The United Kingdom withdrew from the EU in January 2020.

Our 2016 survey was conducted according to the University of Zurich's policy for human subjects research and approved by Stanford University's Institutional Review Board (protocol ID: 34881). Our 2022 survey was approved by Stanford University's Institutional Review Board (protocol ID 34881) and ETH Zurich's Ethics Committee (protocol IRB00007709).

Descriptive statistics on the number of respondents per country in each survey wave are shown in Table S1. Descriptive statistics for the distributions of gender, age, income, political ideology, and education are displayed in Tables S2 and S3.

Sample weights In both survey waves in 2022 and 2016 our sample is somewhat skewed towards more educated and younger respondents. This skew is common in surveys with online panels [33]. To address these imbalances we follow the design described in our pre-analysis plan and match our sample to the demographic margins in each country using entropy balancing [26]. In particular, we adjust the sample so that it matches the distribution of each country on age, gender, and educational attainment. For gender we match on the % female on the population. For age we match on three age categories, including the % aged 18-39, the % aged 40-59, and the % aged 60+. For education we match on three categories of highest educational attainment, including the % below upper secondary education, the % with upper secondary or post-secondary non-tertiary education, and the % with tertiary education. We trim the weights at 6. For all weighted analyses we drop 147 respondents from the 2016 survey wave and 120 respondents from the 2022 survey wave for whom weights cannot be constructed due to missing data on the covariates. We calculated the population margins using the most recently available statistics from the OECD at the time of each survey wave. For age and gender we used the OECD Population Statistics (http://stats.oecd.org/; file EAG_NEAC_29062022222713553). For education we use the table on the share of population by educational attainment in the OECD Education at a Glance database (http://stats.oecd.org/).

We conduct both weighted and unweighted analyses. The unweighted results, which are similar to the weighted estimates, are provided for all main analyses in Figures S1–S5. Further, we also construct an alternative set of weights that match our sample to each country's political

ideological distribution in addition to age, gender, and educational attainment as described above. For political ideology, we use placement on the 0-10 left-right ideology scale, and we match on three categories: left (0-4), center (5), and right (6-10). For this alternative set of weights, we again use entropy balancing and trim the weights at 6. We calculated the population margins for political ideology for each country using the most recently available statistics from the European Election Study and the European Social Survey, as both of these sources employ the same 0-10 left-right ideology scale we employed in our survey. For our 2022 sample, we use the 2019 European Election Study for all countries except for Norway and Switzerland, for which we use the 2018 European Social Survey. For our 2016 sample, we use the 2016 European Social Survey for all countries except for Denmark and Greece, for which we use the 2014 European Election Study. Again, the results are similar to the original weighted estimates. These alternative weighted results are provided for all main analyses in Figures S6–S10.

Experimental design We leverage a fully randomized paired profiles conjoint design [24] to measure the importance of specific asylum-seeker characteristics for voters' support to welcome asylum seekers into their countries. Randomized conjoint experiments are widely used across the social sciences and marketing research in industry to measure multidimensional preferences over objects or persons that are comprised of multiple attributes, such as consumer products, political candidates, job seekers, etc. (for a review see [25, 37]). [29] found that the paired profiles conjoint design performs best among several conjoint and vignette designs to reduce social desirability bias and replicate real-world behavior (see also [39]).

Each respondent was presented with five pairs of profiles of hypothetical asylum seekers displayed side-by-side (see Figure ED1 for an example). The profiles described hypothetical asylum seekers with nine attributes, including the asylum seeker's age, proficiency in the host country language, previous occupation, religion, consistency of the asylum testimony, special vulnerabilities, country of origin, reason for migrating, and gender. As explained in [5], the attributes and attribute values were selected in consultation with asylum policy experts from the Migration Policy Group, UNHCR, and the Swiss Refugee Council, and based on the detailed handbook [41, 42] that the

Swiss State Secretariat of Migration provides for its asylum officers. This handbook specifies the topics on which the officers are required to elicit information during the asylum interviews. The goal was to ensure that we captured the most relevant characteristics that officials typically consider when deciding on asylum claims. In addition, we also included attributes the previous academic literature had identified as important for generating support for the admission of immigrants [32]. Table ED1 describes the full list of attributes and the possible values each attribute could take.

The conjoint design in the 2022 wave was almost identical to the one in the 2016 wave, allowing us to also see how public attitudes have changed. There was only one minor difference. Specifically, for 2022 we added a "War" level for the attribute "Reason for migrating" given the salience of the Russia-Ukraine war. However, we also perform analyses while omitting profiles with this war level to enable a fully identical comparison with the 2016 design.

In addition, the 2022 wave also included an additional experiment where, earlier in the survey, we randomized a set of frames that were shown to respondents. The frames are described in the Survey text subsection below. Unless otherwise noted, all analyses of the conjoint experiment pool over the frames, as we did not find effects of the frames (more detail below).

Outcomes After each pair of asylum-seeker profiles was shown, we asked respondents to perform two tasks displayed on the same screen. First, we asked respondents to rate each profile separately on a scale from 1 (absolutely send the applicant back) to 7 (definitely allow the applicant to stay) to measure how supportive they would be of allowing the hypothetical asylum seeker to stay in the host country. We use the answers to this question to construct our rating outcome variable. Second, for each pair we asked respondents to pick the one asylum seeker that they would prefer to be allowed to stay in the country. So in contrast to the rating task where respondents can support or reject both profiles, for this latter question they are forced to make a choice between the two profiles in each pair. We use the answers to this question to construct our forced choice outcome variable. It is coded as 1 for the preferred profiles and 0 for the rejected profiles. The Survey text subsection below describes the exact question wording, and Table S7 describes the dependent

variables used for analysis in greater detail.

Survey text

A.1.1 Conjoint

The following text was shown to respondents to introduce them to the conjoint section of the survey.

Prelude:

"Now we would like to show you the profiles of potential applicants for asylum in Europe. You will be shown pairs of asylum seekers, along with several of their attributes. We would like to know your opinion regarding whether you would be in favor of sending each applicant back to their country of origin or allowing them to stay in [Respondent's Country].

In total, we will show you five comparison pairs. Please take your time when reading the descriptions of each applicant. People have different opinions about this issue, and there are no right or wrong answers."

Questions:

1. *(Rating)* "On a scale from 1 to 7, where 1 indicates that [Respondent's Country] should absolutely send the applicant back to their country of origin and 7 indicates that [Respondent's Country] should definitely allow the applicant to stay, how would you rate each of the asylum

seekers described above?"

2. *(Choice)* "Now imagine that you had to choose one applicant who would be allowed to stay in [Respondent's Country], and the other applicant would be sent back to their own country of origin. Which of the two applicants would you personally prefer to be allowed to stay in [Respondent's Country]?"

Figure ED1 provides an example of a pair of profiles evaluated by respondents. In addition to the conjoint component, the survey instrument also contained a number of questions that measured various characteristics and attitudes of the respondents. Tables S4-S6 describe these additional variables.

A.1.2 Feeling Thermometer

Respondents were asked about their relative feelings toward their compatriots as well as asylum seekers from various countries of origin. Specifically, they were asked the following:

"We would like to get your feelings toward different groups of people. Below we'll show you the name of a group and we'd like you to rate that group using something we call the feeling thermometer. Ratings between 50 degrees and 100 degrees mean that you feel favorable and warm toward the group. 100 degrees is the highest level and means that you feel very favorable and warm toward the group. Ratings between 0 degrees and 50 degrees mean that you don't feel favorable toward the group and that you don't care too much for that group. 0 degrees is the lowest level and means that you feel very unfavorable and cold toward the group. You would rate the group at the 50 degree mark if you don't feel particularly warm or cold toward the group.
How would you rate:"

- “citizens from [Respondent’s Country]”
- “asylum seekers from Afghanistan”
- “asylum seekers from Eritrea”
- “asylum seekers from Iraq”
- “asylum seekers from Kosovo”
- “asylum seekers from Pakistan”
- “asylum seekers from Syria”
- “asylum seekers from Ukraine”

For each group, respondents provided a rating between 0 and 100.

A.1.3 General Asylum Preferences

Respondents were asked about their general preferences for increasing/decreasing the number of people granted asylum. Specifically, they were asked the following two questions:

Asylum in Home Country:
“Do you think [Respondent’s Country] should increase or decrease the number of people it grants asylum to?”

- Greatly increase
- Increase
- Neither increase nor decrease
- Decrease
- Greatly increase

Asylum in Europe:

"Do you think European countries should increase or decrease the number of people it grants asylum to?"

- Greatly increase
- Increase
- Neither increase nor decrease
- Decrease
- Greatly increase

A.1.4 Frames

At the beginning of the survey, after instructing respondents that they would be asked about European asylum issues, respondents were each randomly shown one of four possible frames. The frames included the following:

1. *(General)* "Your opinions on these matters are extremely important these days, as the number of asylum applications in Europe is expected to continue to rise over the next several years. There is ongoing unrest in regions across the entire world, including in the Middle East, Eastern Europe, Africa, Latin America, and elsewhere. As a result, asylum seekers arriving into Europe are extremely diverse, and the surge of asylum seekers from many countries around the world is likely to continue into the future."

2. *(Mediterranean)* "Your opinions on these matters are extremely important these days, as the number of asylum applications in Europe is expected to continue to rise over the next several years. A particular problem over the past several years has been caused by ongoing unrest in countries in the Middle East and Africa, such as Syria, Iraq, Eritrea, and Sudan. As a result, large numbers of asylum seekers have been arriving into European countries on the

Mediterranean Sea. The surge of asylum seekers via the Mediterranean is likely to continue into the future."

3. *(Afghanistan)* "Your opinions on these matters are extremely important these days, as the number of asylum applications in Europe is expected to continue to rise over the next several years. A particular problem has been caused by the United States' recent military withdrawal from Afghanistan and subsequent takeover by the Taliban. As a result, large numbers of Afghans have been arriving and seeking asylum in countries across Europe. The surge of asylum seekers from Afghanistan is likely to continue into the future."

4. *(Ukraine)* "Your opinions on these matters are extremely important these days, as the number of asylum applications in Europe is expected to continue to rise over the next several years. A particular problem has been caused by the recent Russian invasion of Ukraine and the resulting instability there. As a result, large numbers of Ukrainians are fleeing across the border into countries in Eastern and Central Europe. Many of these Ukrainians will seek asylum in countries in Europe, and the surge of asylum seekers from Ukraine is likely to continue into the future."

Survey translations We designed the survey instrument in English and then professionally translated in each of the country's languages. We also professionally back-translated the translated questionnaires to verify the quality and accuracy of the translations.

Statistical analysis Our analyses follow a preregistered analysis plan available at the Center for Open Science registry (`https://osf.io/jd8n3/`). All analyses were pre-specified except those contained in Figures 4 and 5, for which we subset the analysis by asylum seekers' country of origin and religion and respondent's ideology, respectively. While we registered the main analysis of the fraction of of asylum-seeker profiles accepted (Figure 3), we only investigated the subsample effects during the manuscript writing stage. In addition, the secondary analysis of the ESS data, suggested by a reviewer and conducted during the revision process, is also not pre-specified. The analysis plan was posted on May 18, 2022, the day when fieldwork began for the 2022 wave of the

survey. In addition, unless otherwise noted, all of our analyses pool the data across the different frames since we found no meaningful effects of the frames (more detail below).

A.1.5 AMCEs

Each of the approximately 33,000 respondents across the two waves evaluated five pairs of profiles, resulting in a total of approximately 330,000 asylum seeker profiles being evaluated. Since the attribute values were randomly assigned across respondents and profiles, we can estimate the so-called average marginal component-specific effects (AMCEs) which measure the average causal effect of each attribute on respondents' acceptance of an asylum seeker [24]. We use linear (weighted) least squares regression to regress the rating and choice outcomes (see Table S7) on sets of indicator variables that measure the values of each attribute while omitting one level of each attribute as the reference category and clustering the standard errors by respondent. All regressions employ the sample weights, unless otherwise noted. Note that the results are similar regardless of whether we use the rating or choice outcome, so we focus the presentation of the results mainly on the latter. The robustness of the results when using the rating outcome is shown in Figures S11 and S12. In addition, we also compute the AMCEs as described above for different subsets of respondents based upon respondent characteristics (see Figures ED2, ED3, and ED4) and the host country (see Figures S14, S15, and S16).

A.1.6 Percentage of Profiles Accepted

In addition to evaluating how different asylum-seeker attributes affect support, we also evaluate overall support for asylum seekers by computing the overall percentage of profiles that were accepted by respondents. To do so, we employ a dichotomized version of the Rating outcome in which every profile that was rated higher than 4 (on the 1-7 Rating scale) is coded as having been accepted, and otherwise not accepted. We then use this to compute the percentage of profiles that

are accepted. In addition, to facilitate comparison across the 2022 and 2016 results, profiles that were randomly assigned the "War" level for the "Reason for Migrating" attribute in the 2022 data are omitted from the analysis.

To account for clustering by respondent (i.e. the fact that each respondent evaluated 10 profiles) as well as give equal weight to each respondent (since we omit "War" profiles for the 2022 data), we first compute the percentage of profiles accepted by each respondent, and then perform estimation and statistical inference by analyzing the data at the respondent level. The sample weights are employed to compute weighted mean and weighted variance estimates, and 95% confidence intervals are constructed.

These results are computed separately for the 2016 and 2022 data, along with the difference, and results are presented when pooling across all respondents as well as separately by respondent country (Figure 3). In analyses that were not pre-registered, the results are also presented when subsetting to profiles of particular asylum-seeker country of origin and religion (Figure 4) and when subsetting to respondents with left versus right political ideology (Figure 5).

A.1.7 Additional Analyses

The following describes additional analyses performed.

AMCEs conditional upon Reason for Migrating. To investigate whether the inclusion of "War" as a new level in the "Reason for migrating" attribute in 2022 affected our ability to compare the results across our 2022 and 2016 survey waves, we analyzed the influence of the "War" level on the effects of the other attributes. Specifically, in the 2022 data, we separate all of the conjoint profiles into two subsets: those with "War" as the "Reason for migrating" and those with any other level as the "Reason for migrating". Separately for each subset, we then re-estimate the AMCEs for all of the other attributes, and then compare each AMCE across the two subsets. As shown

in Figure S13, there is virtually no difference in the results across the two subsets. Of the 24 comparisons, only one has a difference with a p-value below 0.05.

Categorical rejecters. We categorize individual respondents as "categorical rejecters" if they gave a rating of lower than 4 to all of the profiles they viewed. We then compute the proportion of respondents who are categorical rejecters. We do this separately for 2016 and 2022, as well as computing the difference, and we do this while pooling across all respondent countries as well as separately for each country (Figure ED5). The sample weights are employed to compute weighted mean and weighted variance estimates, and 95% confidence intervals are constructed.

European solidarity and preference for Ukrainian asylum seekers. As detailed above, we compute the AMCEs for different subsets of respondents based upon respondent characteristics. One dimension of particular interest across which we subset relates to the respondents' sentiments of solidarity with Europe / the West. To measure this, we create a European Solidarity index using two questions asked of respondents. The first asked about the respondent's view of the European Union (EU): "Generally speaking, do you think that [Respondent's Country's] membership of the European Union is...?"[2] Answer options (and numerical codings used for index construction) included: A very good thing (2), A good thing (1), Neither good nor bad (0), A bad thing (-1), and A very bad thing (-2). The second question asked about about the respondent's view of the North Atlantic Treaty Organization (NATO): "[Respondent's Country's] membership in NATO is a good thing."[3] Answer options (and numerical codings) included: Agree strongly (2), Agree (1), Neither agree nor disagree (0), Disagree (-1), and Disagree strongly (-2). We added these two items together to create a European Solidarity index, and then created an indicator for High European Solidarity by coding as a 1 any value above the median value, and 0 otherwise.

We begin by estimating the AMCEs of all attributes separately for respondents with a 1 vs.

[2]For countries not in the EU, the question was modified to "Generally speaking, if [Respondent's Country] were to join the EU, do you think that [Respondent's Country's] membership of the European Union would be...?"
[3]For countries not in NATO, the question was modified to "[Respondent's Country's] membership in NATO would be a good thing (if [Respondent's Country] were to join NATO)."

0 for High European Solidarity, with the results shown in Figure S18. To focus specifically on the effect of an asylum seeker being from Ukraine, we then collapse all of the other non-Ukraine levels in the country of origin attribute to create a binary indicator for whether an asylum seeker is from Ukraine or not. We then estimate the marginal effect of this Ukraine indicator on the probability of respondent choice separately for respondents with a 1 vs. 0 for High European Solidarity. As can be seen in the upper panel of Figure S19, the Ukraine effect is about 50% larger for the high European Solidarity subset relative to the low European Solidarity subset (i.e. a 7.5 percentage-point effect vs. a 5 percentage-point effect). This provides tentative evidence that European solidarity is a source of the Ukraine effect.

We then further investigate the precise role of European solidarity in driving the Ukraine effect in an analysis that was not pre-registered. Specifically, we distinguish between treatment effect heterogeneity across respondents with different levels of European solidarity (i.e. the fact that the Ukraine marginal effect is different for respondents with a 1 vs. 0 for High European Solidarity) and the possibility that the difference in the Ukraine effect can be causally attributed to European solidarity itself. To do so, we employ the framework for causal moderation as presented in [27], using the parallel-regression approach to control for a number of respondent characteristics that are upstream of European solidarity sentiments and could themselves be responsible for the observed effect heterogeneity. Specifically, we control for respondent country, gender, income, political ideology, age, education, level on a nationalism index, and level on a cosmopolitanism index. In addition, the sample weights are employed, and the standard errors are clustered by respondent. The bottom panel of Figure S19 shows the results, comparing this causal moderation effect (bottom bar of bottom panel) to the simple difference in the subset effects (upper bar of bottom panel). As can be seen, the heterogeneity in the Ukraine effect across low and high European solidarity subsets is robust to this causal moderation analysis. While it is impossible to control for every possible variable that might account for this heterogeneity, we do control for several variables of high theoretical importance; hence, the robustness of this result provides meaningful (even if tentative) evidence that European solidarity is indeed one of the drivers of the Ukraine effect.

In contrast to these results, we do not find any evidence that perceptions of the threat posed by Russia influences the Ukraine marginal effect, from the perspective of either simple treatment effect heterogeneity or causal moderation. To assess this, we perform the same analysis as described above using a Russian Threat index (in place of the European Solidarity index), upon which we also perform a median split. The results are shown in Figures S22-S23. The Russian Threat index is an additive index comprised of the following four questions. “Do you think that Russia’s economic and political power is a major threat, a minor threat, or not a threat to [Respondent’s Country]?” Answer options (and numerical codings used for index construction) included: Major threat (2), Minor threat (1), and Not a threat (0). “How concerned are you about Russia using military force against [Respondent’s Country]?” Answer options (and numerical codings) included: Extremely concerned (4), Very concerned (3), Somewhat concerned (2), Not very concerned (1), and Not at all concerned (0). “How strongly do you agree or disagree with each of the following statements regarding the North Atlantic Treaty Organization (NATO) and Russia? NATO should intervene in Ukraine against the ongoing Russian invasion.” Answer options (and numerical codings) included: Agree strongly (2), Agree (1), Neither agree nor disagree (0), Disagree (-1), and Disagree strongly (-2). “Russia’s invasion of Ukraine represents a threat to all of Europe.” Answer options (and numerical codings) included: Agree strongly (2), Agree (1), Neither agree nor disagree (0), Disagree (-1), and Disagree strongly (-2).

Effects of frames. To assess the effects of the frames, we first fit the linear (weighted) least squares regression described above and used for Figure 2 (regressing the forced choice outcome on indicators for each attribute level) separately across the four frames. This allows for visual inspection and comparison. Figure S24 shows the results. To further investigate, we then fit the main regression (pooling all the frames) while also including indicators for the frames (with the General frame as the omitted reference level) and interactions between each frame and each attribute-level indicator. We then assess the distribution of the p-values associated with the interactions from that regression using a quantile-quantile plot. Figure S25 shows the results. There is no systematic evidence of important interactions. Note that the null distribution of no interactions (theoretical quantiles) assumes independent estimates, which is certainly violated given sets of interactions that

are associated with each attribute. Nonetheless, the presence of meaningful nonzero interactions would still produce p-values that are systematically and substantially lower than their theoretical values, especially at the lower quantiles. There are 4 p-values that are lower than 0.05. The expected number of p-values that are lower than 0.05 under the null hypothesis of no interactions is 4.2 (given that there are 84 interactions in the model).

In addition, we also investigate the effect of the frames on the feeling thermometer scores by fitting a linear (weighted) least squares regression of the feeling thermometer score for each group on indicators for the different frames (with the General frame as the reference category). As shown in Figure S26, we find no evidence of an effect of the frames on the feeling thermometer scores either.

General asylum preferences. As described in Section A above, we also asked about the respondents' preferences for increasing/decreasing the number of people granted asylum in (a) their home country and (b) Europe more broadly. We dichotomize their answers into an indicator for support for an increase (i.e. a 1 for answer options Greatly increase and Increase, and 0 otherwise). We then compute the percentage who support an increase. These results are computed separately for the 2016 and 2022 data, along with the difference, and results are presented when pooling across all respondents as well as separately by respondent country. Figure ED6 shows the results for increasing asylum in the respondents' home country, and Figure ED7 shows the results for increasing asylum in Europe. The sample weights are employed to compute weighted mean and weighted variance estimates, and 95% confidence intervals are constructed. This analysis was not pre-registered.

We further break these results down by respondent left-right ideology, shown in Figures S20 and S21. Consistent with the left-right divide in our conjoint results (as in Figure 5 in the main text), we also see a general left-right divide in responses to these direct survey questions. However, we do not see robust evidence of polarization over time——that is, the left-right divide has not on average increased by much, if at all, from 2016 to 2022. In terms of support for increasing the

number of asylum seekers in Europe, we find virtually no evidence of polarization even at the level of individual countries: within each country, the change in support from 2016 to 2022 is substantively similar and almost entirely statistically indistinguishable between the left and right. On support for increasing asylum seekers within one's own country, the average polarization (i.e. degree to which the difference between left and right has increased) is also substantively very small. Yet there is some heterogeneity in this measure across a few countries. For example, in the United Kingdom, we see polarization over time largely due to increasing support on the left (and constant support on the right). On the other hand, there has been a decrease in polarization in one country, Hungary. In totality, while there are a few country-specific idiosyncrasies, there do not appear to be any general patterns of left-right polarization over time.

Immigration Attitudes Over Time. One potential concern with our analyses is that our survey only covers two points in time, 2016 at the height of the Syrian crisis and 2022 at the height of the Ukrainian crisis. To examine how public attitudes have evolved over a longer time period, including before and after the 2016 crisis, we conducted supplementary analyses using data from the European Social Survey (ESS). The ESS is a biennial cross-national survey of attitudes using face-to-face interviews with probability samples that are representative of persons aged 15 and over residing within private households in each country (see `www.europeansocialsurvey.org/`). We leverage all available ESS data from the first round in 2002 to the most recent round from 2020. For our analyses we trim the ESS sample to the same 15 countries included in our own surveys to make the results comparable. Most of these 15 countries appear in all ten waves of the ESS.[4]

The ESS does not include randomized conjoint designs like we leverage in our own survey to measure detailed attitudes towards refugees. Yet, the ESS offers two questions that are asked in all waves and allow us to track general attitudes towards two related types of immigration. The first question asks "To what extent do you think [Respondent Country] should allow people from the

[4]Austria is missing in ESS rounds 4-6; the Czech Republic is missing in round 3; Denmark is missing in rounds 8 and 10; Greece is missing in rounds 3 and 6-9; the United Kingdom is missing in round 10; and Italy is missing in rounds 2-5 and 7.

poorer countries outside Europe people to come and live here?” The second question asks “How about people of a different race or ethnic group from most [Respondent Country] people?” There are four answer options including “Allow many to come and live here”, “Allow some”, “Allow a few”, and “Allow none.”

Figure S27 displays immigration attitudes as measured by both questions for the pooled ESS sample across all ten rounds from 2002-2020. Consistent with the results from our own survey, there is a high degree of stability in immigration attitudes over time. If anything, there is a small increase in support for both types of immigration in the most recent waves. This indicates that the comparison point of the Syrian crisis in 2016 is not a particular low point in the trend of European support for immigration. In fact, support for immigration did not erode with the onset of the 2016 crisis even though it was heavily politicized and debated, involving even dramatic measures such as the closing of borders within the EU.

Figure S28 shows the results when replicating the analysis only for the sample of countries that are included in all ten waves of the ESS survey and the results are similar, again showing a high degree of stability over time and if anything a small increase in support in the most recent waves. Figures S29 and S30 show the trends in immigration attitudes broken down by country. Again, the results are fairly consistent across countries in that attitudes are broadly stable over time and if anything become somewhat more positive in most recent waves. One exception to this is Sweden where attitudes turn somewhat more negative in the most recent ESS wave, but note that Sweden in general is the most supportive country in the sample. Overall these findings from the ESS are highly consistent with the results from our own surveys. This analysis was not pre-registered

Distribution of Choice Probabilities over Conjoint Profiles. Figure 2 in the main text displays the AMCE estimates for each attribute using the Choice outcome. The estimates were produced using a linear probability model, as it is shown in [24] that a linear probability model (fit with indicator variables corresponding to the levels of each attribute) recovers unbiased and consistent estimates of AMCEs with conjoint data. In addition to considering the AMCEs, another

evaluation of the collective influence of the conjoint attributes can proceed by evaluating the distribution of the probability of choice across profiles. For this purpose, it is useful to consider more flexible modeling approaches that, rather than having a focus on consistent/unbiased estimation of AMCEs, are tailored to producing an accurate prediction function with well-calibrated predicted probabilities.

Hence, to conduct such an evaluation, we employ stochastic gradient boosted trees. We fit boosted tree models that predict the choice outcome as a function of the attributes, with the tree depth and number of trees selected via 5-fold cross-validation. To validate the models, which we fit separately for the 2016 and 2022 data, we assess their probability calibration using their cross-validated predicted probabilities. These calibration results are shown in Figure S31. The points in the figure correspond to the ventile bins of predicted probabilities. For each ventile, the x-axis displays the mean predicted probability in the bin and the y-axis displays the true proportion/mean of positives in the bin. For visual evaluative purposes, the points are connected and a dashed identity line is overlaid. As can be seen, the points trace tightly along the identity line, demonstrating well-calibrated models.

We then apply these models to generate predicted probabilities over the full set of all possible profiles—i.e. a predicted probability for all of the possible combinations of attribute levels (170,100 combinations for the 2022 data and 136,080 combinations for the 2016 data, since the 2016 data did not contain the War level for the Reason for Migrating attribute). We visualize the distribution of the probabilities with histograms, shown in Figure S32. The vertical red line in the figure denotes the probability of 0.5, which would be the single predicted probability for all profiles under the null/intercept-only model. As can be seen, the conjoint attributes generate a large amount of variation in collective preferences for specific asylum-seeker profiles. For our 2022 survey results, the minimum and maximum probabilities are 0.15 and 0.84, with a standard deviation of 0.12, and for our 2016 survey results, the minimum and maximum probabilities are 0.17 and 0.83, with a standard deviation of 0.12. This analysis was not pre-registered.

Number of asylum-seeker profiles accepted across respondents. As a final evaluation of within-respondent variation, we focus on the dichotomized version of the Rating outcome for respondents' evaluations of each asylum-seeker profile they viewed in their conjoint tasks. As described above, profiles rated higher than 4 (on the 1-7 Rating scale) were coded as having been accepted, and otherwise not accepted. Using this measure, we can then determine for each individual respondent the number of profiles they accepted out of the ten total profiles they viewed. Figure S33 shows the distribution of the number of profiles accepted across respondents. As can be seen, a sizable majority of respondents accepted greater than zero and less than ten of the profiles they viewed. In other words, only a limited minority of respondents unconditionally accepted (or failed to accept) all of the profiles they saw, indicating the general importance of variation in asylum-seeker attributes for determining support at the individual level. This analysis was not pre-registered.

B Supplementary Text

Notes on conjoint plots and regression tables

1. Plots of the conjoint AMCEs contained in the figures in the main text and the SI are based on linear (weighted) least squares regression estimates. The dots with horizontal lines indicate point estimates with cluster-robust 95% confidence intervals. The unfilled dots on the zero line denote the reference category for each asylum-seeker attribute.

2. Unless otherwise noted, all regression models corresponding to the conjoint AMCEs employ the choice outcome as the dependent variable (*Forced Choice*). Where specified, certain models also employ the other two other dependent variables *Binary Rating* and *Scaled Rating*. See Table S7 for more detail on the alternative dependent variables.

3. Unless otherwise noted, all regression models regress the dependent variable on the full set of asylum-seeker profile attributes (a dummy variable for all levels except for one reference level per attribute). The reference categories for the nine attributes are as follows. Asylum

Testimony: No inconsistencies, Gender: Female, Country of Origin: Syria, Age: 21 Years, Previous Occupation: Unemployed, Vulnerability: None, Reason for Migrating: Political persecution, Religion: Christian, Language Skills: Fluent.

4. Unless otherwise noted, all analyses use the post-stratification weights.

5. The number of respondents for the weighted models is slightly lower than the total number of respondents since the data necessary for constructing the post-stratification weights were missing for 147 respondents from the 2016 survey wave and 120 respondents from the 2022 survey wave.

C Supplementary Figures and Tables

Table S1: Number of respondents per country and survey wave

Country:	Wave 2016	Wave 2022	Total
Austria	1206	1001	2207
Czech Republic	1202	1001	2203
Denmark	1201	995	2196
France	1203	997	2200
Germany	1200	993	2193
Greece	1200	1002	2202
Hungary	1200	1001	2201
Italy	1200	996	2196
Netherlands	1200	993	2193
Norway	1202	1001	2203
Poland	1201	999	2200
Spain	1203	999	2202
Sweden	1203	1000	2203
Switzerland	1208	997	2205
United Kingdom	1201	1001	2202
Total	18030	14976	33006

Tables

Table S2: Summary statistics for gender, age, income, and ideology. By country and survey wave.

	Survey	Gender (%)	Age (%)					Income Quintile (%)					Ideology (%)		
	Year	Female	< 29	30-39	40-49	50-59	60+	First	Second	Third	Fourth	Fifth	Left	Center	Right
Austria	2016	50.0	22.0	19.0	23.9	18.2	17.0	29.1	23.5	22.1	16.7	7.7	33.7	37.5	28.8
Austria	2022	50.4	19.9	18.9	23.7	19.8	17.8	27.4	23.5	21.5	20.1	7.0	33.8	37.3	29.0
Czech Republic	2016	51.0	41.9	28.0	16.1	8.9	5.1	14.8	19.3	22.5	28.0	15.2	22.5	42.8	34.7
Czech Republic	2022	52.1	40.3	27.6	17.7	8.5	6.0	14.1	16.3	22.4	23.6	23.6	21.5	47.5	31.1
Denmark	2016	50.0	20.1	21.1	21.9	20.0	17.0	26.1	19.6	24.7	15.6	13.2	36.7	25.1	38.1
Denmark	2022	51.2	18.7	20.1	22.3	21.0	17.9	25.1	21.0	21.8	16.2	15.5	34.0	25.1	40.9
France	2016	51.1	23.3	20.2	21.2	20.1	15.2	28.6	22.9	16.9	22.4	9.0	29.5	34.6	35.9
France	2022	51.5	23.0	20.7	20.8	19.9	15.7	25.1	20.1	16.4	17.9	20.6	29.3	33.1	37.6
Germany	2016	50.0	19.8	18.0	25.0	21.1	16.1	24.6	22.5	21.0	20.2	11.3	40.9	35.6	23.5
Germany	2022	50.2	20.4	17.8	23.3	21.8	16.7	20.7	16.7	23.9	21.7	16.5	39.9	38.0	22.2
Greece	2016	47.0	33.2	29.4	24.7	10.1	2.6	37.3	28.4	18.8	10.5	4.9	36.2	35.8	28.0
Greece	2022	47.7	35.7	23.9	21.1	13.4	6.0	30.0	26.0	21.5	15.2	7.2	29.2	37.1	33.6
Hungary	2016	51.0	43.2	28.4	14.2	8.1	6.1	17.5	20.2	15.2	21.2	25.6	18.7	48.3	33.0
Hungary	2022	51.5	42.7	26.5	17.6	7.8	5.5	16.3	15.4	13.9	18.0	36.4	23.0	39.9	37.2
Italy	2016	50.0	19.0	22.0	23.0	19.0	17.0	27.3	25.8	21.8	18.3	6.6	35.2	26.2	38.5
Italy	2022	50.4	18.3	21.6	22.7	19.6	17.9	21.6	23.2	25.1	21.1	8.9	36.1	29.0	34.8
Netherlands	2016	50.0	22.0	17.2	22.8	21.0	17.0	30.2	21.8	19.3	18.8	9.4	27.7	33.2	39.2
Netherlands	2022	50.7	18.9	18.6	22.4	22.1	18.0	19.4	14.9	22.6	21.5	21.6	27.1	30.1	42.8
Norway	2016	49.0	22.2	21.8	21.0	19.0	16.1	27.7	26.0	19.2	13.2	13.2	33.8	24.0	42.3
Norway	2022	50.4	19.7	22.1	21.6	19.9	16.8	24.9	23.9	22.0	15.4	13.6	32.5	27.9	39.7
Poland	2016	49.0	19.0	29.8	25.1	15.3	10.8	14.6	21.6	20.3	23.9	19.7	23.2	38.6	38.2
Poland	2022	49.4	18.5	27.4	27.1	17.5	9.4	11.4	19.0	21.6	23.0	24.9	26.9	35.1	37.9
Spain	2016	50.1	21.9	25.1	21.9	17.0	14.0	23.3	25.4	18.2	16.4	16.5	50.8	22.5	26.7
Spain	2022	50.1	21.4	24.3	22.3	17.4	14.5	17.1	26.6	24.4	21.3	10.2	45.7	25.5	28.7
Sweden	2016	49.1	22.1	20.0	20.9	19.0	18.0	21.5	15.2	19.7	18.0	25.1	37.4	26.0	36.6
Sweden	2022	49.7	21.5	20.0	20.3	19.3	18.9	13.4	16.0	27.2	24.6	18.6	36.2	25.0	38.8
Switzerland	2016	49.8	22.1	19.9	22.8	19.2	16.0	32.3	29.1	19.6	14.1	4.2	29.1	30.2	40.7
Switzerland	2022	51.0	20.7	19.6	23.3	19.7	16.9	33.0	25.3	18.3	16.4	6.9	32.5	34.1	33.4
United Kingdom	2016	52.0	26.0	18.8	21.0	18.2	16.1	28.6	21.3	18.7	18.9	12.4	26.7	42.8	30.5
United Kingdom	2022	51.5	25.1	18.4	20.9	18.9	16.8	25.2	22.0	18.8	21.6	12.4	29.9	42.4	27.8

Table S3: Summary statistics for educational attainment. By country and survey wave.

	Survey	Educational Attainment (ISCED 2011 level; %)				
	Year	Level: 0-2	Level: 3	Level: 4-5	Level: 6	Level: 7-8
Austria	2016	8.5	52.9	20.1	5.3	12.9
Austria	2022	8.3	48.8	19.6	8.5	14.8
Czech Republic	2016	5.2	55.9	15.1	9.9	13.8
Czech Republic	2022	9.1	53.7	18.1	7.9	10.3
Denmark	2016	22.3	37.1	10.2	22.1	7.6
Denmark	2022	22.2	33.3	7.8	23.4	12.7
France	2016	9.6	44.9	20.8	8.6	15.9
France	2022	6.7	40.8	19.2	12.9	20.3
Germany	2016	4.8	36.0	27.4	13.7	12.2
Germany	2022	5.3	31.4	27.2	15.7	16.7
Greece	2016	3.6	32.3	18.8	29.4	15.9
Greece	2022	1.2	31.3	15.5	33.5	18.0
Hungary	2016	4.2	49.2	18.2	18.8	9.2
Hungary	2022	5.5	50.6	15.3	19.0	9.1
Italy	2016	13.2	48.2	4.1	10.8	23.3
Italy	2022	13.2	53.6	3.9	12.8	16.5
Netherlands	2016	31.8	33.5	7.4	11.7	15.2
Netherlands	2022	27.8	36.7	7.7	15.0	12.6
Norway	2016	13.2	34.6	16.6	23.2	11.4
Norway	2022	12.0	38.0	13.5	23.5	12.1
Poland	2016	9.2	35.2	12.7	13.6	29.0
Poland	2022	14.4	37.1	8.7	13.1	26.2
Spain	2016	23.1	18.7	15.9	17.5	24.9
Spain	2022	20.9	18.8	17.4	16.5	26.2
Sweden	2016	11.5	37.2	27.3	11.1	11.1
Sweden	2022	6.9	37.0	23.2	18.2	14.1
Switzerland	2016	10.1	49.3	23.4	7.4	9.2
Switzerland	2022	15.8	47.5	14.9	9.5	11.8
United Kingdom	2016	17.4	37.0	14.4	20.6	10.7
United Kingdom	2022	16.2	34.8	13.5	23.6	12.0

Notes: Levels are based on the International Standard Classification of Education (ISCED) as follows: Level 0 is “Less than primary”, level 1 is “Primary”, level 2 is “Lower secondary”, level 3 is “Upper secondary”, level 4 is “Post-secondary non tertiary”, level 5 is “Short-cycle tertiary”, level 6 is “Bachelor or equivalent”, level 7 is “Master or equivalent”, and level 8 is “Doctoral or equivalent.”

Table S4: Other Variables

The table describes other respondent characteristics measured in the survey and used in our analyses.

Variable	Description
Age	Self-reported age.
Gender	Self-reported gender.
Education	Self-reported highest level of education achieved. The customized country-specific educational level questionnaire options used by the European Social Survey were employed for each country, and the respondents' educational levels were mapped onto the harmonized European version of the International Standard Classification of Education (EISCED) scale. The scale contains seven major categories: less than lower secondary (1), lower secondary (2), lower tier upper secondary (3), upper tier upper secondary (4), advanced vocational/sub-degree (5), lower tertiary education/BA level (6), and higher tertiary education/$\geq$ MA level (7).
Income	Self-reported household income. Respondents' household income levels were measured using the customized country-specific household income decile bins constructed in the European Social Survey (the most recent available version of the European Social Survey was used for each country in the sample). For certain analyses, these deciles were coarsened into quintiles.
Employment status	Self-reported employment status. Respondents were asked to answer the following question: "Which of these options best describes your situation (in the last seven days)?" Answer options included: "Paid employee (including temporary leave of absence due to maternity/paternity, accident, illness or vacation)", "Self-employed (e.g. freelancer, independent contractor, or family-owned business), "Student (excluding employer-sponsored education), "Unemployed, actively searching for a job", "Unemployed, not actively searching", "Chronic illness or permanent disability", "Retired", and "Working at home, caring for children or others."
Political ideology	Self-reported placement on the left-right political ideological spectrum. Ideological placement was measured using a standard self-identification scale ranging from 0 on the left to 10 on the right. The question wording used to measure the respondents' ideology was as follows: "In politics people often talk of 'left' and 'right'. On this scale from 0 (left) to 10 (right), where would you classify your own political views?"

Table S5: Other variables, continued.

Variable	Description
Native-born	Self-reported indicator for having been born in home country.
Nationalism index	Multi-construct measure of nationalism. This index is an additive index comprised of agreement/disagreement with the following four prompts: "I would rather be a citizen of [Respondent's Country] than of any other country in the world"; "Generally speaking [Respondent's Country] is a better country than most other countries"; "[Respondent's Country] should follow its own interests, even if this leads to conflicts with other nations"; and "[Respondent's Country's] government should just try to take care of the wellbeing of [Respondent's Country's] citizens and not get involved with other nations." For each prompt, answer options (and numerical codings used for index construction) included: Agree strongly (2), Agree (1), Neither agree nor disagree (0), Disagree (-1), and Disagree strongly (-2).
Cosmopolitanism index	Multi-construct measure of cosmopolitanism. This index is an additive index comprised of agreement/disagreement with the following four prompts: "Although the media often reports about national and international events and developments, this news is seldom as interesting as the things that happen directly in our own community and neighborhood"; "I feel more like a citizen of the world than of any country"; "I enjoy learning about different cultures"; and "[Respondent's Country] has many things to learn from other countries." For the first prompt, answer options (and numerical codings used for index construction) included: Agree strongly (-2), Agree (-1), Neither agree nor disagree (0), Disagree (1), and Disagree strongly (2). For the remaining prompts, answer options (and numerical codings used for index construction) included: Agree strongly (2), Agree (1), Neither agree nor disagree (0), Disagree (-1), and Disagree strongly (-2).
General asylum preferences, home country	Self-reported preference for increasing or decreasing the number of people granted asylum in respondent's home country. Respondents were asked: "Do you think [Respondent's Country] should increase or decrease the number of people it grants asylum to?" Answer options included greatly increase (2), increase (1), neither increase nor decrease (0), decrease (-1), and greatly decrease (-2).
General asylum preferences, Europe	Self-reported preference for increasing or decreasing the number of people granted asylum in Europe. Respondents were asked: "Do you think European countries should increase or decrease the number of people it grants asylum to?" Answer options included greatly increase (2), increase (1), neither increase nor decrease (0), decrease (-1), and greatly decrease (-2).
General immigration preferences	Self-reported preference for increasing or decreasing the number of immigrants admitted in respondent's home country. Respondents were asked: "In general, do you think [Respondent's Country] should increase or decrease the number of immigrants it admits (i.e. not only asylum-seekers but all immigrants)? "Answer options included greatly increase (2), increase (1), neither increase nor decrease (0), decrease (-1), and greatly decrease (-2).

Table S6: Other variables, continued.

Variable	Description
EU support	Self-reported support for membership in the European Union (EU). Respondents were asked: "Generally speaking, do you think that [Respondent's Country's] membership of the European Union is...?" (For countries not in the EU, the question was modified to "Generally speaking, if [Respondent's Country] were to join the EU, do you think that [Respondent's Country's] membership of the European Union would be...?") Answer options included: A very good thing (2), A good thing (1), Neither good nor bad (0), A bad thing (-1), and A very bad thing (-2).
NATO support	Self-reported support for membership in the North Atlantic Treaty Organization (NATO). Respondents were prompted with the following statement: "[Respondent's Country's] membership in NATO is a good thing." (For countries not in NATO, the prompt was modified to "[Respondent's Country's] membership in NATO would be a good thing (if [Respondent's Country] were to join NATO).") Response options included: Agree strongly (2), Agree (1), Neither agree nor disagree (0), Disagree (-1), and Disagree strongly (-2).
Russia economic-political threat	Self-reported perception of the economic and political threat posed by Russia. Respondents were asked: "Do you think that Russia's economic and political power is a major threat, a minor threat, or not a threat to [Respondent's Country]?" Answer options included: Major threat (2), Minor threat (1), and Not a threat (0).
Russia military threat	Self-reported perception of the military threat posed by Russia. Respondents were asked: "How concerned are you about Russia using military force against [Respondent's Country]?" Answer options included: Extremely concerned (4), Very concerned (3), Somewhat concerned (2), Not very concerned (1), and Not at all concerned (0).
Ukraine intervention	Self-reported belief in the need for NATO to intervene in Ukraine against Russia. Respondents were prompted with the following statement: "NATO should intervene in Ukraine against the ongoing Russian invasion." Answer options included: Agree strongly (2), Agree (1), Neither agree nor disagree (0), Disagree (-1), and Disagree strongly (-2).
Russia threat to Europe	Self-reported view on the threat to Europe posed by Russia's invasion of Ukraine. Respondents were prompted with the following statement: "Russia's invasion of Ukraine represents a threat to all of Europe." Answer options included: Agree strongly (2), Agree (1), Neither agree nor disagree (0), Disagree (-1), and Disagree strongly (-2).

Table S7: Outcome Variables

The table describes the dependent variables used in the conjoint analysis.

**Variable**	**Description**
Forced Choice	Binary indicator for whether or not a profile was the preferred profile in its respective pair. It is based on respondents' answers to the following question: "Now imagine that you had to choose one applicant who would be allowed to stay in [Respondent's Country], and the other applicant would be sent back to their own country of origin. Which of the two applicants would you personally prefer to be allowed to stay in [Respondent's Country]?"
Binary Rating	Dichotomized version of the Rating outcome. The Rating outcome was measured based on respondents' answers to the following question: "On a scale from 1 to 7, where 1 indicates that [Respondent's Country] should absolutely send the applicant back to their country of origin and 7 indicates that [Respondent's Country] should definitely allow the applicant to stay, how would you rate each of the asylum seekers described above?" The *Binary Rating* variable was then coded as a 1 for profiles rated higher than 4 (out of 7) and 0 otherwise, thus serving as an indicator for acceptance.
Scaled Rating	Scaled version of the Rating outcome, where the original 1-7 coding is rescaled to vary between 0 and 1.

Table S8: Mean feeling thermometer scores. The results in the table below are those used to construct Fig. 1 of the main text.

	Estimate	Standard Error	95% CI
Asylum seekers from Afghanistan	44.55	0.22	[44.12, 44.99]
Asylum seekers from Eritrea	44.06	0.22	[43.63, 44.50]
Asylum seekers from Iraq	43.31	0.22	[42.88, 43.75]
Asylum seekers from Kosovo	46.89	0.22	[46.46, 47.32]
Asylum seekers from Pakistan	42.65	0.22	[42.21, 43.09]
Asylum seekers from Syria	44.87	0.23	[44.41, 45.32]
Asylum seekers from Ukraine	62.50	0.22	[62.07, 62.94]
Compatriots	79.48	0.18	[79.14, 79.83]

Table S9: Pooled conjoint regression results. The results in the table below are those used to construct the conjoint plot presented in Fig. 2 of the main text.

	2016	2022	Difference
(Intercept)	0.647^{*}	0.588^{*}	
	(0.008)	(0.009)	
**Asylum Testimony:**			
Minor inconsistencies	-0.028^{*}	-0.026^{*}	0.002
	(0.004)	(0.004)	(0.005)
Major inconsistencies	-0.107^{*}	-0.096^{*}	0.011^{*}
	(0.004)	(0.004)	(0.006)
**Gender:**			
Male	-0.058^{*}	-0.060^{*}	-0.002
	(0.003)	(0.003)	(0.004)
**Country of Origin:**			
Afghanistan	-0.021^{*}	-0.007	0.013
	(0.006)	(0.006)	(0.008)
Kosovo	-0.036^{*}	-0.003	0.033^{*}
	(0.006)	(0.006)	(0.008)
Eritrea	-0.012^{*}	-0.005	0.007
	(0.006)	(0.006)	(0.008)
Pakistan	-0.018^{*}	-0.010	0.008
	(0.006)	(0.006)	(0.008)
Ukraine	-0.009	0.049^{*}	0.058^{*}
	(0.006)	(0.006)	(0.008)
Iraq	-0.021^{*}	-0.010	0.011
	(0.006)	(0.006)	(0.008)
**Age:**			
38 Years	-0.007	-0.013^{*}	-0.006
	(0.004)	(0.004)	(0.005)
62 Years	-0.061^{*}	-0.059^{*}	0.002
	(0.004)	(0.004)	(0.006)
**Previous Occupation:**			
Cleaner	0.048^{*}	0.060^{*}	0.012
	(0.005)	(0.006)	(0.008)
Farmer	0.056^{*}	0.069^{*}	0.013
	(0.005)	(0.006)	(0.008)
Accountant	0.080^{*}	0.076^{*}	-0.004
	(0.005)	(0.006)	(0.008)
Teacher	0.092^{*}	0.103^{*}	0.011
	(0.005)	(0.006)	(0.008)
Doctor	0.134^{*}	0.142^{*}	0.008
	(0.005)	(0.006)	(0.008)
**Vulnerability:**			
PTSD	0.013^{*}	0.013^{*}	0.000
	(0.005)	(0.005)	(0.007)
Victim of torture	0.112^{*}	0.098^{*}	-0.014^{*}
	(0.005)	(0.005)	(0.007)
No surviving family	0.040^{*}	0.035^{*}	-0.005
	(0.005)	(0.005)	(0.007)
Handicapped	0.026^{*}	0.028^{*}	0.002
	(0.005)	(0.005)	(0.007)
**Reason for Migrating:**			
Religious persecution	0.005	0.006	0.001
	(0.004)	(0.005)	(0.007)
Ethnic persecution	0.010^{*}	0.015^{*}	0.006
	(0.004)	(0.005)	(0.007)
Economic opportunities	-0.150^{*}	-0.133^{*}	0.016^{*}
	(0.004)	(0.005)	(0.007)
War		0.056^{*}	
		(0.005)	
**Religion:**			
Agnostic	-0.037^{*}	-0.029^{*}	0.008
	(0.004)	(0.004)	(0.005)
Muslim	-0.107^{*}	-0.088^{*}	0.019^{*}
	(0.004)	(0.004)	(0.006)
**Language Skills:**			
Broken	-0.059^{*}	-0.052^{*}	0.007
	(0.004)	(0.004)	(0.005)
None	-0.117^{*}	-0.111^{*}	0.006
	(0.004)	(0.004)	(0.006)
R^2	0.063	0.056	
Adj. R^2	0.063	0.056	
Num. obs.	178740	148460	

$^{*}p < 0.05$

Table S10: Percentage of asylum-seeker profiles accepted, results from 2016, 2022, and difference. The results in the table below are those used to construct Fig. 3 of the main text.

Year	Respondent Country	Estimate	Standard Error	95% CI Lower	95% CI Upper
2016	Pooled	44.54	0.24	44.08	45.01
2016	Austria	43.43	0.88	41.70	45.17
2016	Czech Republic	37.88	0.79	36.32	39.44
2016	Denmark	43.50	0.94	41.66	45.34
2016	France	42.62	0.92	40.82	44.43
2016	Germany	47.39	0.89	45.64	49.15
2016	Greece	46.14	0.97	44.23	48.05
2016	Hungary	41.39	0.89	39.63	43.14
2016	Italy	50.75	0.95	48.89	52.61
2016	Netherlands	43.24	0.90	41.47	45.01
2016	Norway	44.21	0.87	42.49	45.92
2016	Poland	45.10	0.81	43.52	46.68
2016	Spain	57.48	0.98	55.55	59.40
2016	Sweden	43.34	0.96	41.46	45.21
2016	Switzerland	44.04	0.91	42.26	45.82
2016	United Kingdom	37.94	0.90	36.18	39.70
2022	Pooled	49.44	0.28	48.89	49.98
2022	Austria	49.65	1.05	47.59	51.71
2022	Czech Republic	38.15	0.99	36.21	40.08
2022	Denmark	46.42	1.06	44.33	48.50
2022	France	47.31	1.07	45.21	49.40
2022	Germany	52.14	1.09	50.01	54.27
2022	Greece	50.39	1.08	48.27	52.51
2022	Hungary	47.31	1.08	45.19	49.43
2022	Italy	58.14	1.12	55.94	60.34
2022	Netherlands	50.05	1.03	48.03	52.08
2022	Norway	47.65	1.00	45.68	49.62
2022	Poland	50.06	1.07	47.97	52.15
2022	Spain	59.73	1.14	57.50	61.97
2022	Sweden	45.69	1.07	43.60	47.79
2022	Switzerland	49.06	1.06	46.98	51.13
2022	United Kingdom	49.56	1.09	47.41	51.70
Difference	Pooled	4.89	0.37	4.18	5.61
Difference	Austria	6.22	1.37	3.53	8.91
Difference	Czech Republic	0.27	1.27	-2.22	2.75
Difference	Denmark	2.92	1.42	0.14	5.70
Difference	France	4.69	1.41	1.93	7.45
Difference	Germany	4.75	1.41	1.99	7.51
Difference	Greece	4.25	1.45	1.40	7.10
Difference	Hungary	5.93	1.40	3.18	8.68
Difference	Italy	7.39	1.47	4.52	10.27
Difference	Netherlands	6.81	1.37	4.12	9.50
Difference	Norway	3.44	1.33	0.83	6.05
Difference	Poland	4.96	1.34	2.34	7.58
Difference	Spain	2.26	1.51	-0.69	5.21
Difference	Sweden	2.36	1.43	-0.45	5.16
Difference	Switzerland	5.02	1.39	2.28	7.75
Difference	United Kingdom	11.62	1.41	8.85	14.39

Table S11: Difference in percentage of asylum-seeker profiles accepted, between 2022 and 2016 survey waves, for nationality- and religion-based subgroups of asylum seekers. The results in the table below are those used to construct Fig. 4 of the main text.

Respondent Country	Asylum-seeker Subgroup	Estimate	Standard Error	95% CI Lower	95% CI Upper
Pooled	Non-Ukrainians	4.20	0.37	3.46	4.93
Austria	Non-Ukrainians	4.60	1.41	1.84	7.37
Czech Republic	Non-Ukrainians	0.67	1.30	-1.89	3.23
Denmark	Non-Ukrainians	1.75	1.45	-1.10	4.59
France	Non-Ukrainians	3.85	1.44	1.02	6.68
Germany	Non-Ukrainians	3.59	1.43	0.78	6.40
Greece	Non-Ukrainians	3.76	1.48	0.87	6.66
Hungary	Non-Ukrainians	6.38	1.42	3.59	9.17
Italy	Non-Ukrainians	7.54	1.50	4.60	10.48
Netherlands	Non-Ukrainians	5.65	1.41	2.89	8.40
Norway	Non-Ukrainians	2.33	1.37	-0.36	5.01
Poland	Non-Ukrainians	4.89	1.37	2.21	7.57
Spain	Non-Ukrainians	1.85	1.53	-1.15	4.85
Sweden	Non-Ukrainians	0.50	1.46	-2.37	3.37
Switzerland	Non-Ukrainians	4.30	1.43	1.50	7.11
United Kingdom	Non-Ukrainians	10.81	1.44	7.99	13.64
Pooled	Ukrainians	9.37	0.58	8.22	10.51
Austria	Ukrainians	15.72	2.24	11.33	20.11
Czech Republic	Ukrainians	-3.43	2.20	-7.75	0.89
Denmark	Ukrainians	10.19	2.25	5.77	14.61
France	Ukrainians	11.75	2.27	7.30	16.20
Germany	Ukrainians	13.12	2.30	8.62	17.63
Greece	Ukrainians	5.80	2.24	1.41	10.20
Hungary	Ukrainians	1.82	2.30	-2.69	6.33
Italy	Ukrainians	5.49	2.27	1.05	9.93
Netherlands	Ukrainians	14.60	2.24	10.21	18.99
Norway	Ukrainians	11.78	2.22	7.43	16.14
Poland	Ukrainians	6.84	2.21	2.50	11.18
Spain	Ukrainians	4.43	2.26	0.01	8.86
Sweden	Ukrainians	14.94	2.27	10.49	19.39
Switzerland	Ukrainians	9.46	2.23	5.08	13.84
United Kingdom	Ukrainians	17.55	2.22	13.20	21.89
Pooled	Christians	4.36	0.45	3.47	5.24
Austria	Christians	6.81	1.69	3.50	10.12
Czech Republic	Christians	-2.48	1.69	-5.80	0.84
Denmark	Christians	3.68	1.75	0.24	7.11
France	Christians	3.22	1.72	-0.15	6.60
Germany	Christians	4.19	1.75	0.76	7.62
Greece	Christians	5.43	1.77	1.95	8.90
Hungary	Christians	3.00	1.72	-0.37	6.37
Italy	Christians	5.62	1.76	2.17	9.07
Netherlands	Christians	6.31	1.74	2.91	9.71
Norway	Christians	4.06	1.68	0.76	7.36
Poland	Christians	1.28	1.72	-2.09	4.65
Spain	Christians	3.92	1.77	0.46	7.39
Sweden	Christians	4.48	1.77	1.01	7.96
Switzerland	Christians	5.57	1.72	2.20	8.94
United Kingdom	Christians	9.52	1.72	6.15	12.89
Pooled	Muslims	5.40	0.45	4.52	6.29
Austria	Muslims	4.32	1.73	0.93	7.70
Czech Republic	Muslims	2.90	1.61	-0.25	6.05
Denmark	Muslims	2.11	1.74	-1.30	5.52
France	Muslims	4.18	1.75	0.75	7.61
Germany	Muslims	2.72	1.79	-0.79	6.23
Greece	Muslims	4.93	1.77	1.46	8.41
Hungary	Muslims	7.55	1.71	4.20	10.90
Italy	Muslims	9.75	1.78	6.26	13.24
Netherlands	Muslims	6.67	1.72	3.31	10.04
Norway	Muslims	3.05	1.72	-0.33	6.42
Poland	Muslims	8.82	1.69	5.51	12.13
Spain	Muslims	3.00	1.81	-0.55	6.55
Sweden	Muslims	-0.62	1.76	-4.08	2.83
Switzerland	Muslims	7.84	1.71	4.48	11.19
United Kingdom	Muslims	12.55	1.73	9.17	15.93

Table S12: Percentage of asylum-seeker profiles accepted for respondents with left political ideology, results from 2016, 2022, and difference. The results in the table below are those used to construct Fig. 5 of the main text.

Year	Respondent Country	Respondent Ideology	Estimate	Standard Error	95% CI Lower	95% CI Upper
2016	Pooled	Left	54.88	0.42	54.07	55.70
2016	Austria	Left	57.99	1.47	55.09	60.89
2016	Czech Republic	Left	35.55	1.58	32.45	38.66
2016	Denmark	Left	54.49	1.58	51.39	57.60
2016	France	Left	54.67	1.72	51.29	58.06
2016	Germany	Left	57.37	1.28	54.85	59.89
2016	Greece	Left	54.73	1.58	51.63	57.84
2016	Hungary	Left	51.31	2.13	47.12	55.50
2016	Italy	Left	61.25	1.56	58.18	64.32
2016	Netherlands	Left	52.55	1.63	49.35	55.75
2016	Norway	Left	55.07	1.41	52.30	57.85
2016	Poland	Left	48.15	1.68	44.83	51.46
2016	Spain	Left	65.15	1.33	62.54	67.76
2016	Sweden	Left	52.97	1.60	49.81	56.12
2016	Switzerland	Left	61.35	1.54	58.33	64.38
2016	United Kingdom	Left	48.39	1.82	44.80	51.98
2022	Pooled	Left	61.34	0.49	60.39	62.29
2022	Austria	Left	63.27	1.75	59.83	66.71
2022	Czech Republic	Left	35.39	2.01	31.42	39.35
2022	Denmark	Left	57.45	1.88	53.76	61.15
2022	France	Left	58.26	1.95	54.43	62.10
2022	Germany	Left	66.17	1.61	63.01	69.33
2022	Greece	Left	62.28	1.97	58.42	66.15
2022	Hungary	Left	54.94	2.29	50.43	59.45
2022	Italy	Left	66.73	1.83	63.13	70.33
2022	Netherlands	Left	61.32	1.86	57.65	64.98
2022	Norway	Left	59.22	1.70	55.88	62.55
2022	Poland	Left	58.78	2.08	54.69	62.87
2022	Spain	Left	70.86	1.53	67.86	73.86
2022	Sweden	Left	58.45	1.80	54.91	61.99
2022	Switzerland	Left	64.80	1.75	61.35	68.25
2022	United Kingdom	Left	64.96	1.86	61.29	68.62
Difference	Pooled	Left	6.45	0.64	5.20	7.71
Difference	Austria	Left	5.28	2.29	0.79	9.77
Difference	Czech Republic	Left	-0.17	2.56	-5.18	4.85
Difference	Denmark	Left	2.96	2.46	-1.85	7.77
Difference	France	Left	3.59	2.60	-1.51	8.68
Difference	Germany	Left	8.80	2.06	4.76	12.83
Difference	Greece	Left	7.55	2.52	2.61	12.49
Difference	Hungary	Left	3.63	3.12	-2.49	9.75
Difference	Italy	Left	5.48	2.41	0.77	10.19
Difference	Netherlands	Left	8.76	2.47	3.92	13.61
Difference	Norway	Left	4.14	2.20	-0.18	8.47
Difference	Poland	Left	10.63	2.67	5.40	15.87
Difference	Spain	Left	5.70	2.02	1.73	9.67
Difference	Sweden	Left	5.48	2.41	0.76	10.21
Difference	Switzerland	Left	3.44	2.33	-1.13	8.01
Difference	United Kingdom	Left	16.57	2.61	11.46	21.68

Table S13: Percentage of asylum-seeker profiles accepted for respondents with right political ideology, results from 2016, 2022, and difference. The results in the table below are those used to construct Fig. 5 of the main text.

Year	Respondent Country	Respondent Ideology	Estimate	Standard Error	95% CI Lower	95% CI Upper
2016	Pooled	Right	38.54	0.38	37.80	39.28
2016	Austria	Right	30.73	1.41	27.95	33.51
2016	Czech Republic	Right	41.23	1.34	38.58	43.87
2016	Denmark	Right	36.35	1.39	33.62	39.07
2016	France	Right	36.77	1.41	34.00	39.54
2016	Germany	Right	39.60	1.78	36.10	43.11
2016	Greece	Right	42.72	1.88	39.02	46.41
2016	Hungary	Right	35.68	1.41	32.90	38.46
2016	Italy	Right	44.42	1.41	41.65	47.18
2016	Netherlands	Right	39.29	1.44	36.47	42.12
2016	Norway	Right	35.52	1.26	33.03	38.00
2016	Poland	Right	44.64	1.28	42.13	47.15
2016	Spain	Right	50.38	1.77	46.90	53.87
2016	Sweden	Right	36.44	1.41	33.67	39.21
2016	Switzerland	Right	31.34	1.24	28.91	33.77
2016	United Kingdom	Right	36.38	1.55	33.33	39.42
2022	Pooled	Right	42.94	0.44	42.07	43.80
2022	Austria	Right	38.13	1.71	34.76	41.49
2022	Czech Republic	Right	44.24	1.68	40.93	47.55
2022	Denmark	Right	41.24	1.54	38.21	44.28
2022	France	Right	38.79	1.51	35.82	41.77
2022	Germany	Right	41.58	2.01	37.63	45.54
2022	Greece	Right	44.03	1.70	40.70	47.37
2022	Hungary	Right	43.52	1.71	40.17	46.87
2022	Italy	Right	49.32	1.80	45.77	52.86
2022	Netherlands	Right	45.27	1.56	42.20	48.33
2022	Norway	Right	42.75	1.53	39.74	45.77
2022	Poland	Right	50.31	1.66	47.04	53.58
2022	Spain	Right	49.10	2.02	45.12	53.08
2022	Sweden	Right	37.83	1.47	34.93	40.73
2022	Switzerland	Right	35.49	1.66	32.23	38.75
2022	United Kingdom	Right	41.54	2.00	37.61	45.47
Difference	Pooled	Right	4.40	0.58	3.26	5.53
Difference	Austria	Right	7.40	2.22	3.04	11.75
Difference	Czech Republic	Right	3.01	2.15	-1.21	7.23
Difference	Denmark	Right	4.90	2.08	0.83	8.97
Difference	France	Right	2.02	2.07	-2.03	6.08
Difference	Germany	Right	1.98	2.68	-3.28	7.24
Difference	Greece	Right	1.32	2.53	-3.64	6.28
Difference	Hungary	Right	7.84	2.22	3.50	12.18
Difference	Italy	Right	4.90	2.29	0.42	9.38
Difference	Netherlands	Right	5.97	2.12	1.82	10.13
Difference	Norway	Right	7.24	1.99	3.34	11.14
Difference	Poland	Right	5.67	2.10	1.56	9.79
Difference	Spain	Right	-1.28	2.69	-6.56	3.99
Difference	Sweden	Right	1.39	2.04	-2.61	5.39
Difference	Switzerland	Right	4.15	2.07	0.09	8.20
Difference	United Kingdom	Right	5.16	2.53	0.21	10.12

Table S14: Correlations between general immigration attitudes and attitudes toward asylum seekers, results from 2016 and 2022. The results in the table below display the (weighted) correlations between three direct survey questions measuring attitudes toward immigrants and asylum seekers, described in Table S5 (*General immigration preferences*; *General asylum preferences, home country*; and *General asylum preferences, Europe*). The correlations are shown separately for the 2016 and 2022 data.

**Correlations between:** (2016 / 2022)	*General immigration preferences*	*General asylum preferences, home country*	*General asylum preferences, Europe*
General immigration preferences	1.00 / 1.00	0.80 / 0.80	0.66 / 0.64
General asylum preferences, home country	0.80 / 0.80	1.00 / 1.00	0.73 / 0.74
General asylum preferences, Europe	0.66 / 0.64	0.73 / 0.74	1.00 / 1.00

Figures

Figure S1: Unweighted version of Figure 1

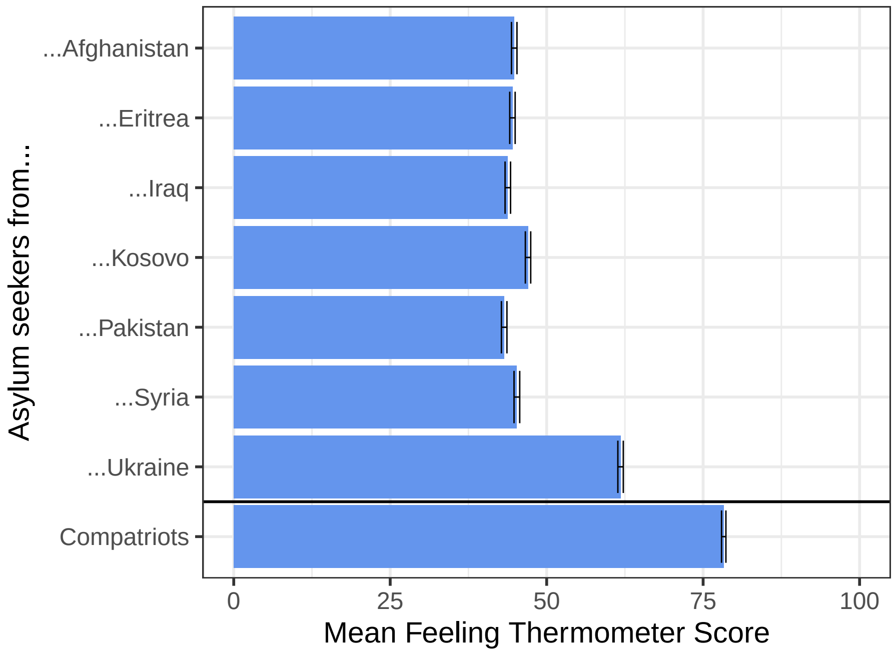

Figure S2: Unweighted version of Figure 2

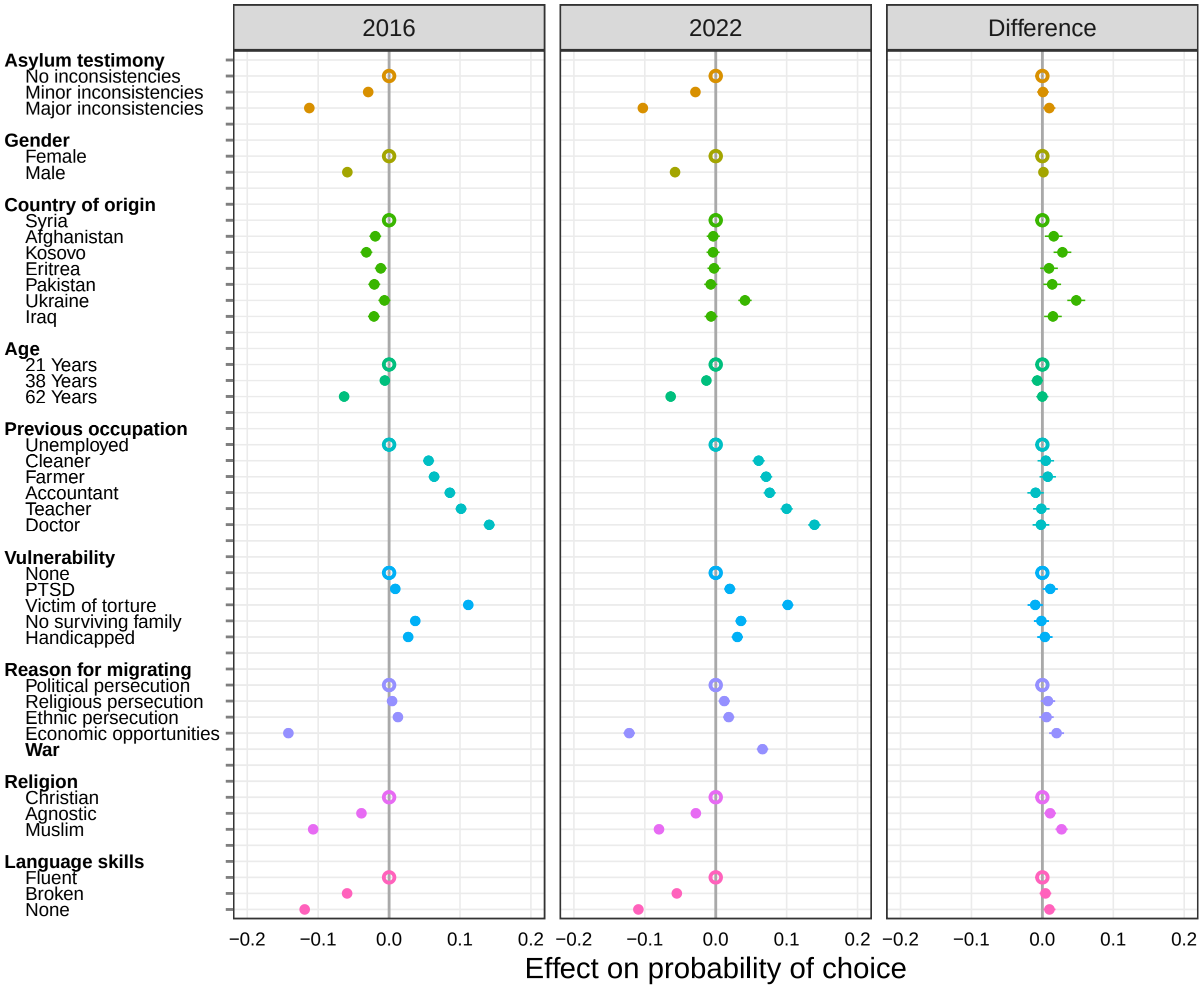

Figure S3: Unweighted version of Figure 3

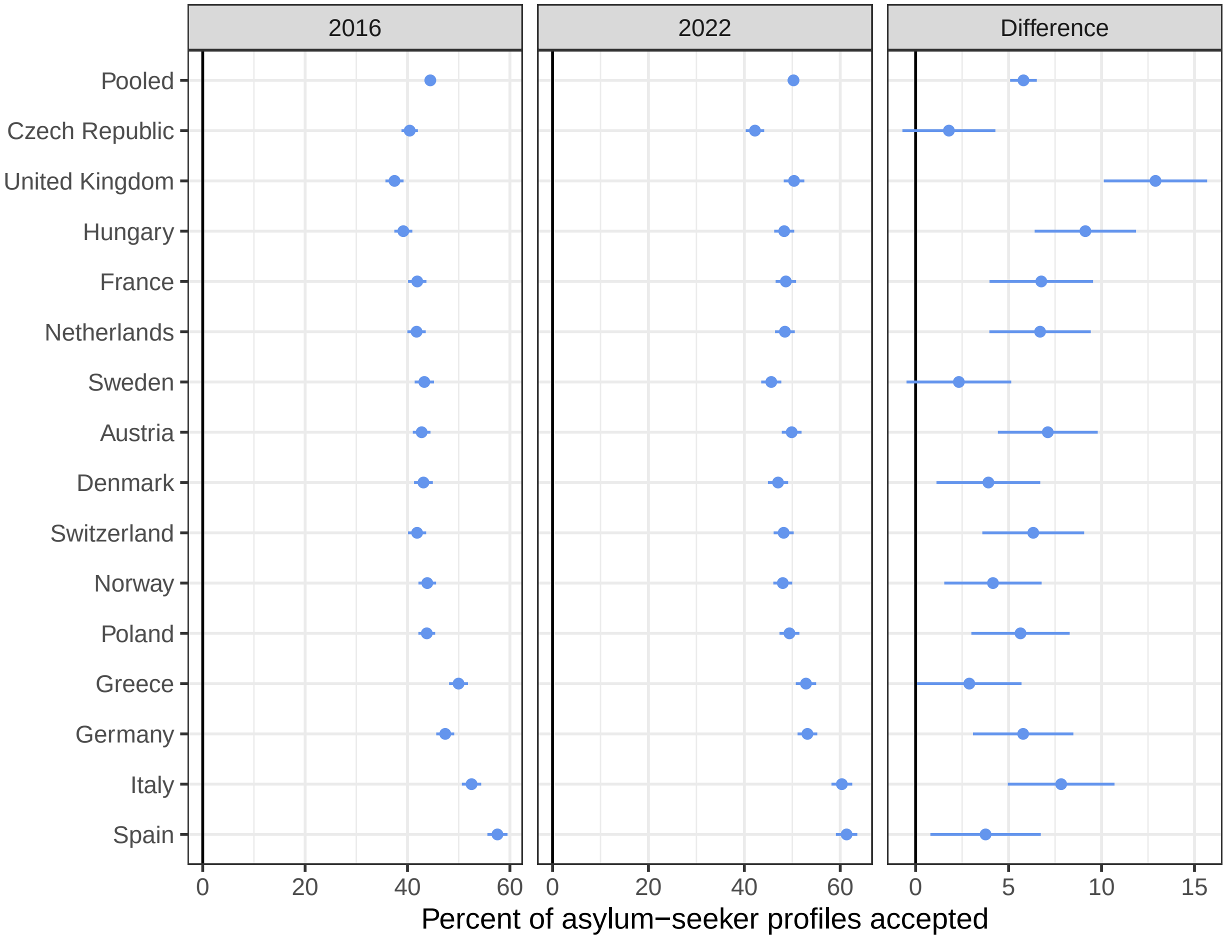

Figure S4: Unweighted version of Figure 4

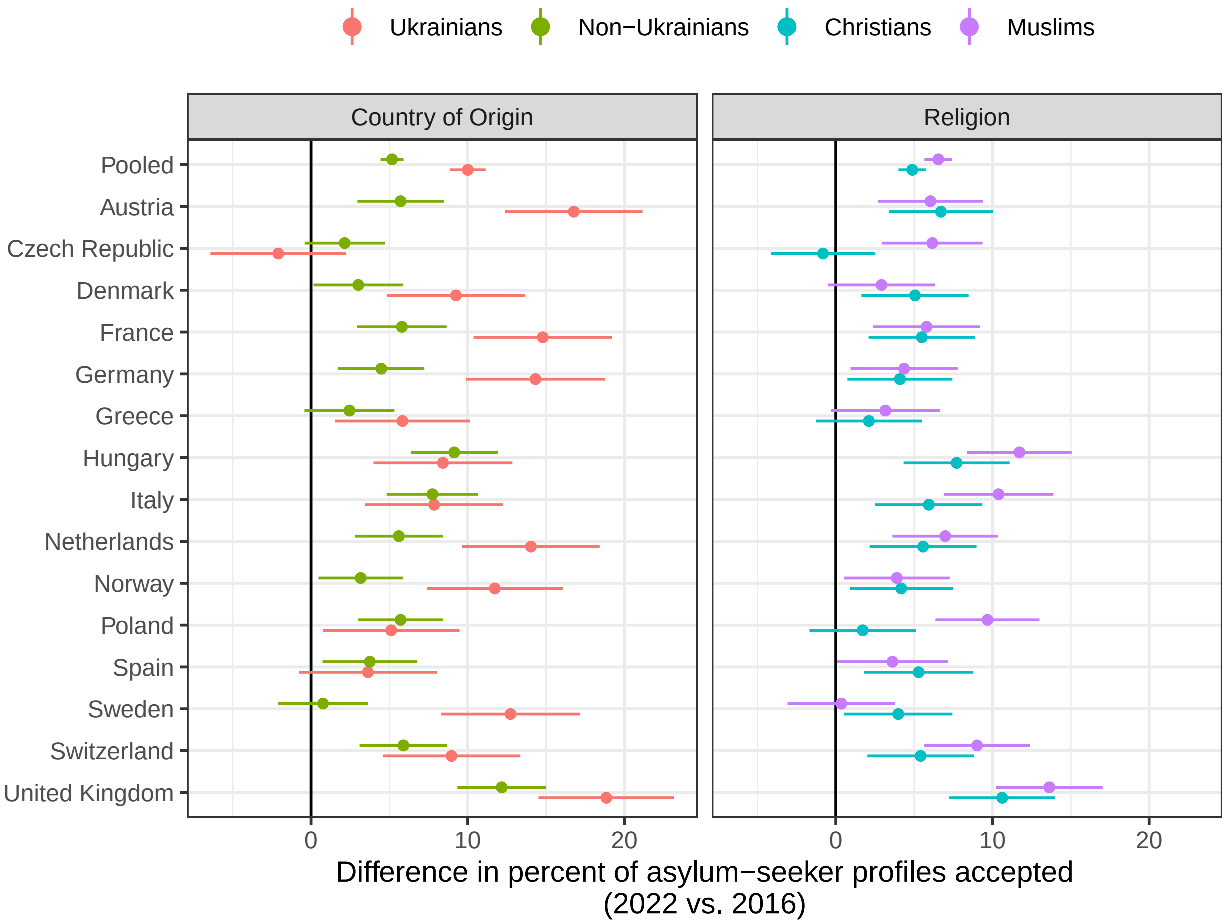

Figure S5: Unweighted version of Figure 5

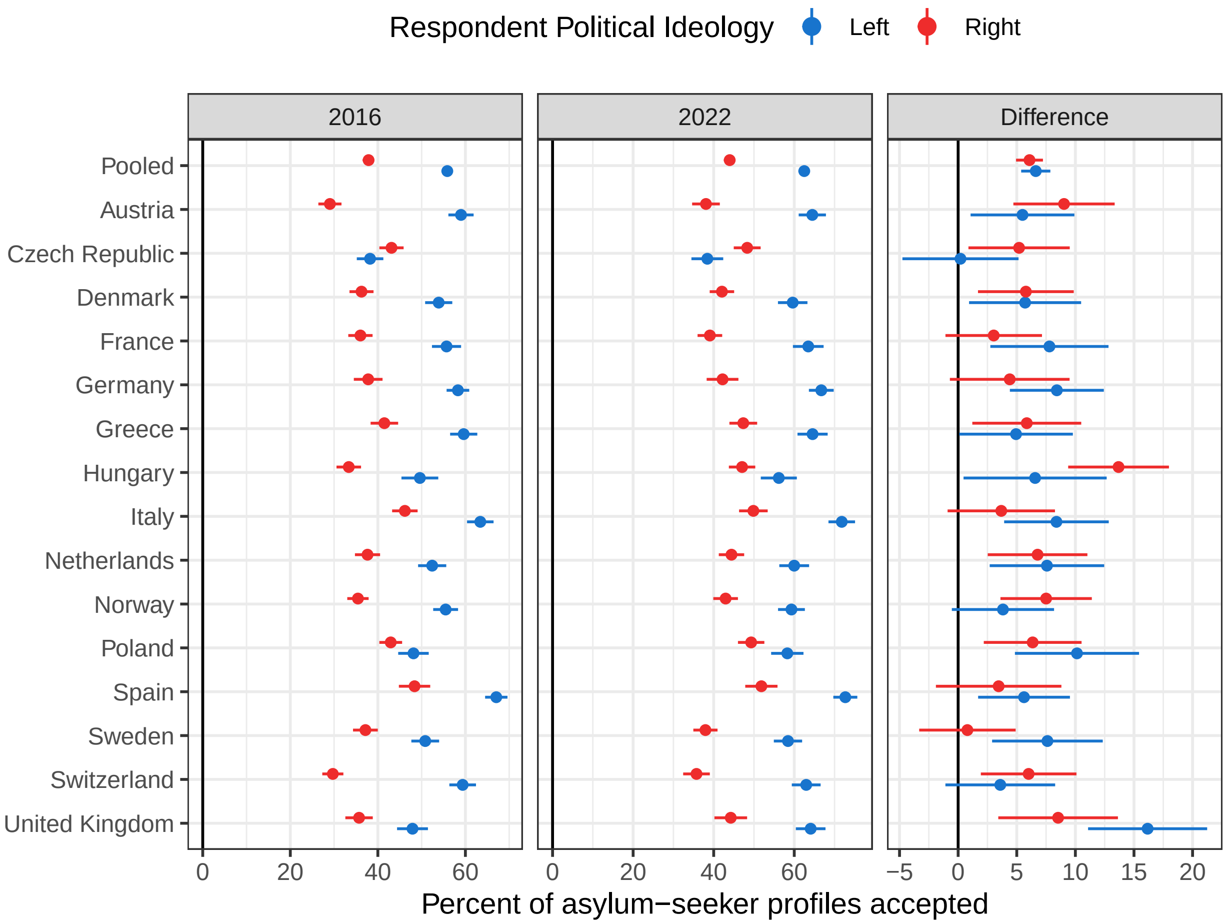

Figure S6: Version of Figure 1 using alternative weights

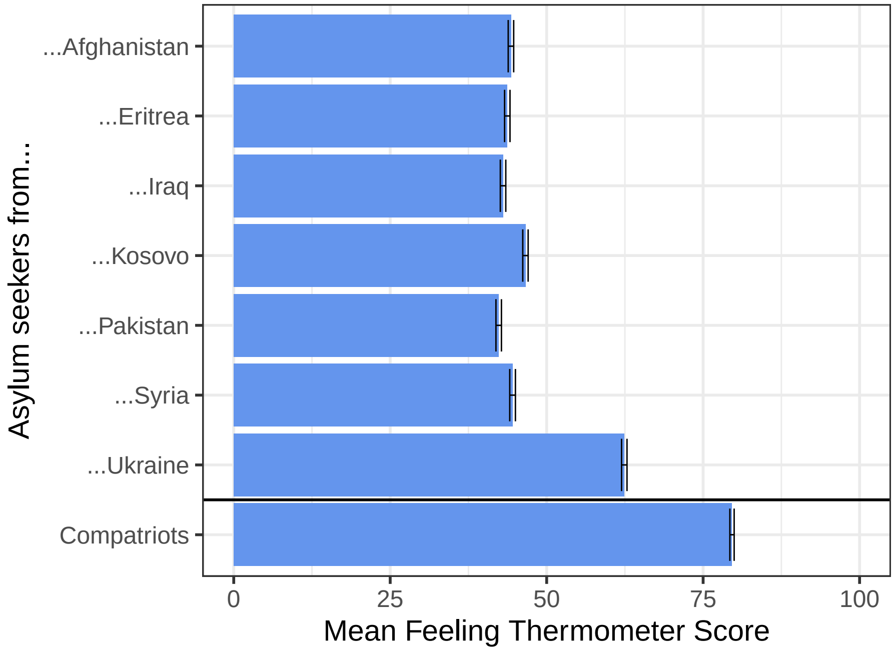

Figure S7: Version of Figure 2 using alternative weights

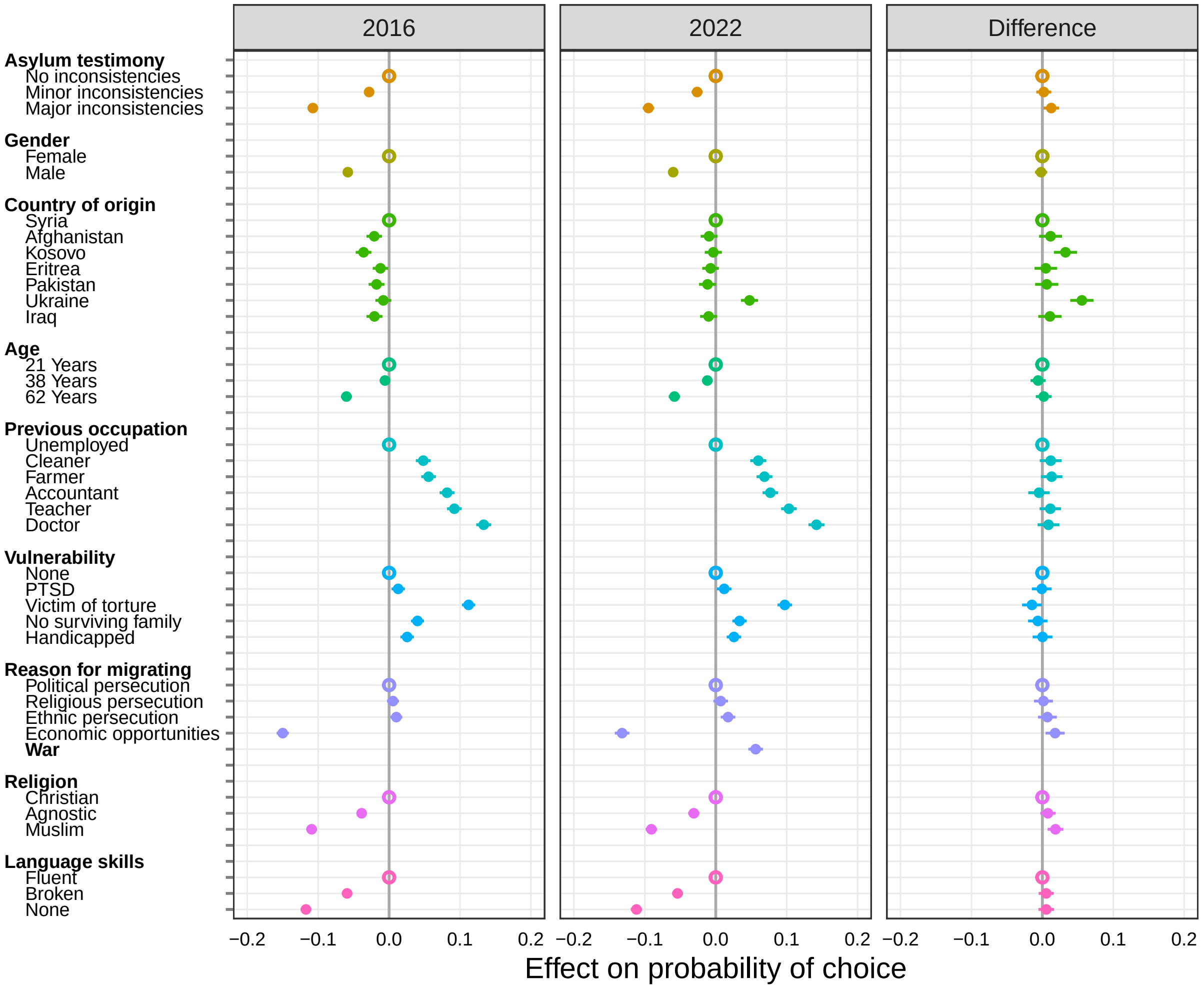

Figure S8: Version of Figure 3 using alternative weights

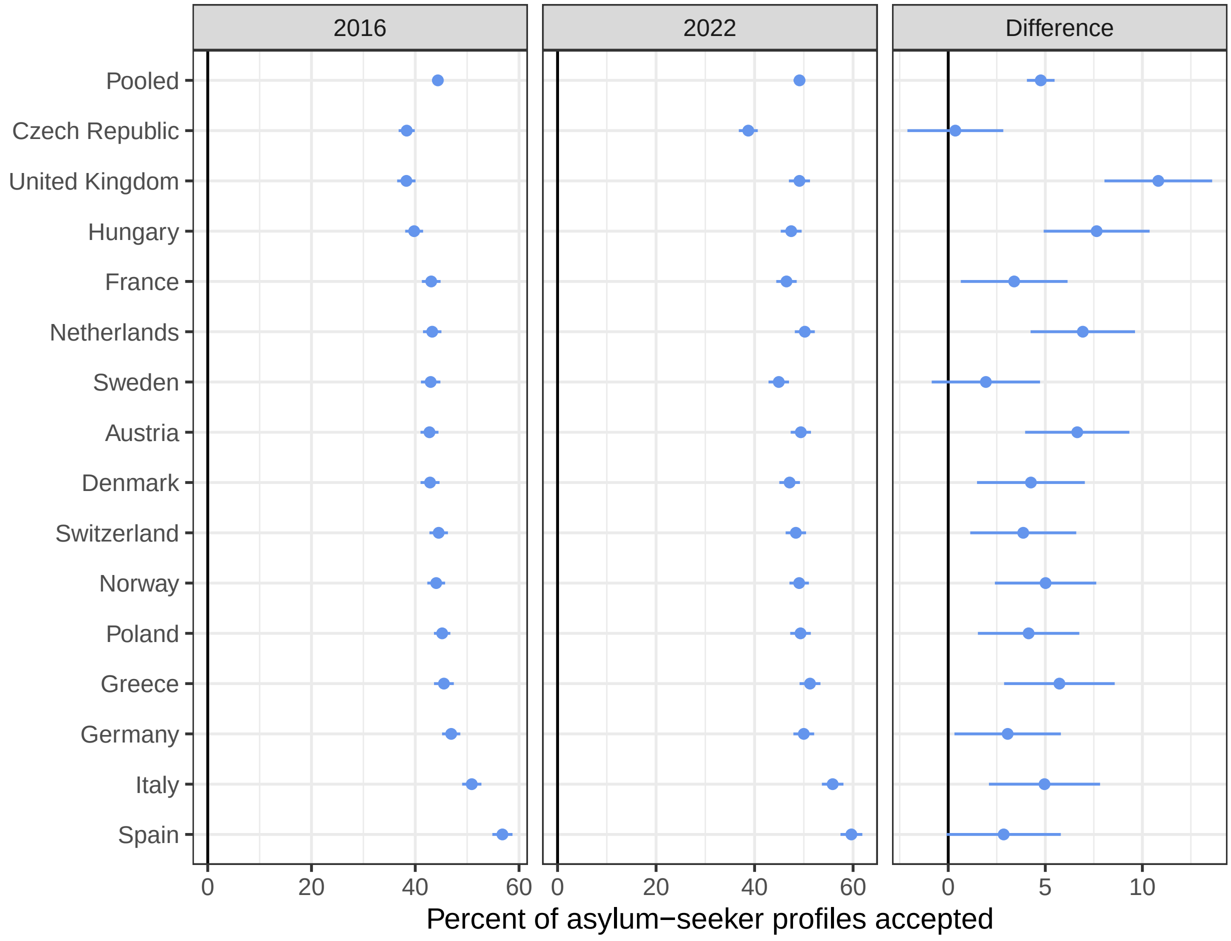

Figure S9: Version of Figure 4 using alternative weights

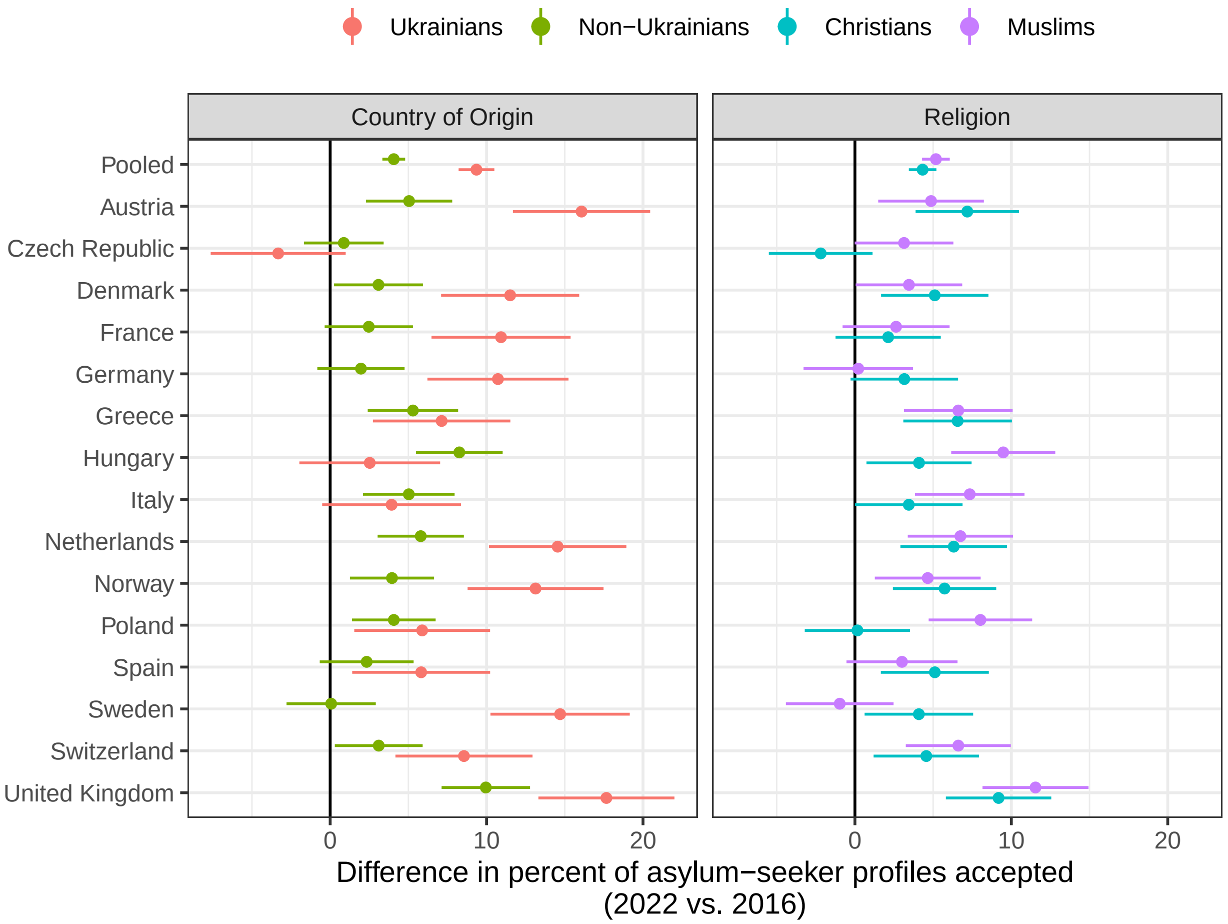

Figure S10: Version of Figure 5 using alternative weights

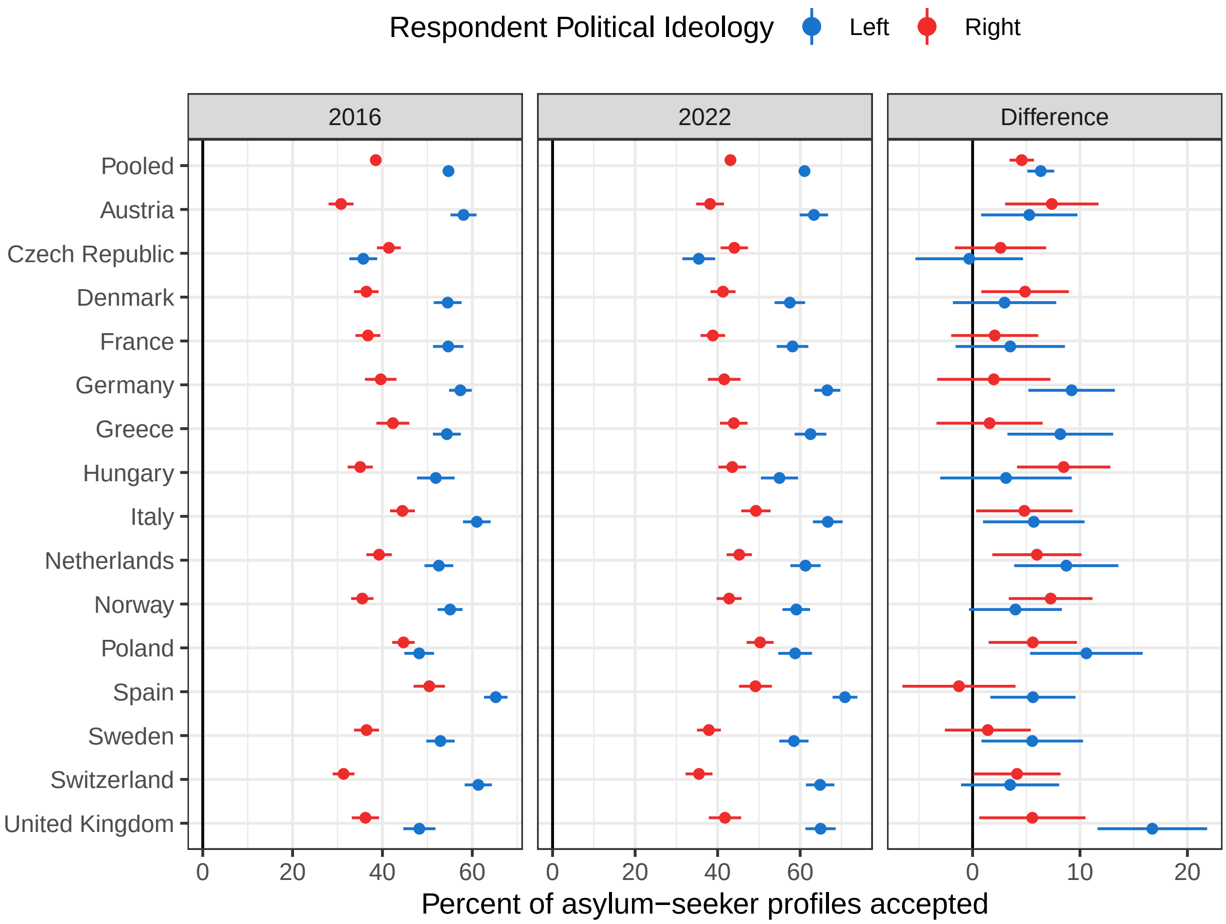

Figure S11: Effects of asylum-seeker attributes on respondent rating, results from 2016, 2022, and difference. The original 1-7 rating is rescaled to 0-1 (Scaled Rating outcome variable) for this analysis to facilitate comparison with the results using the Forced Choice outcome.

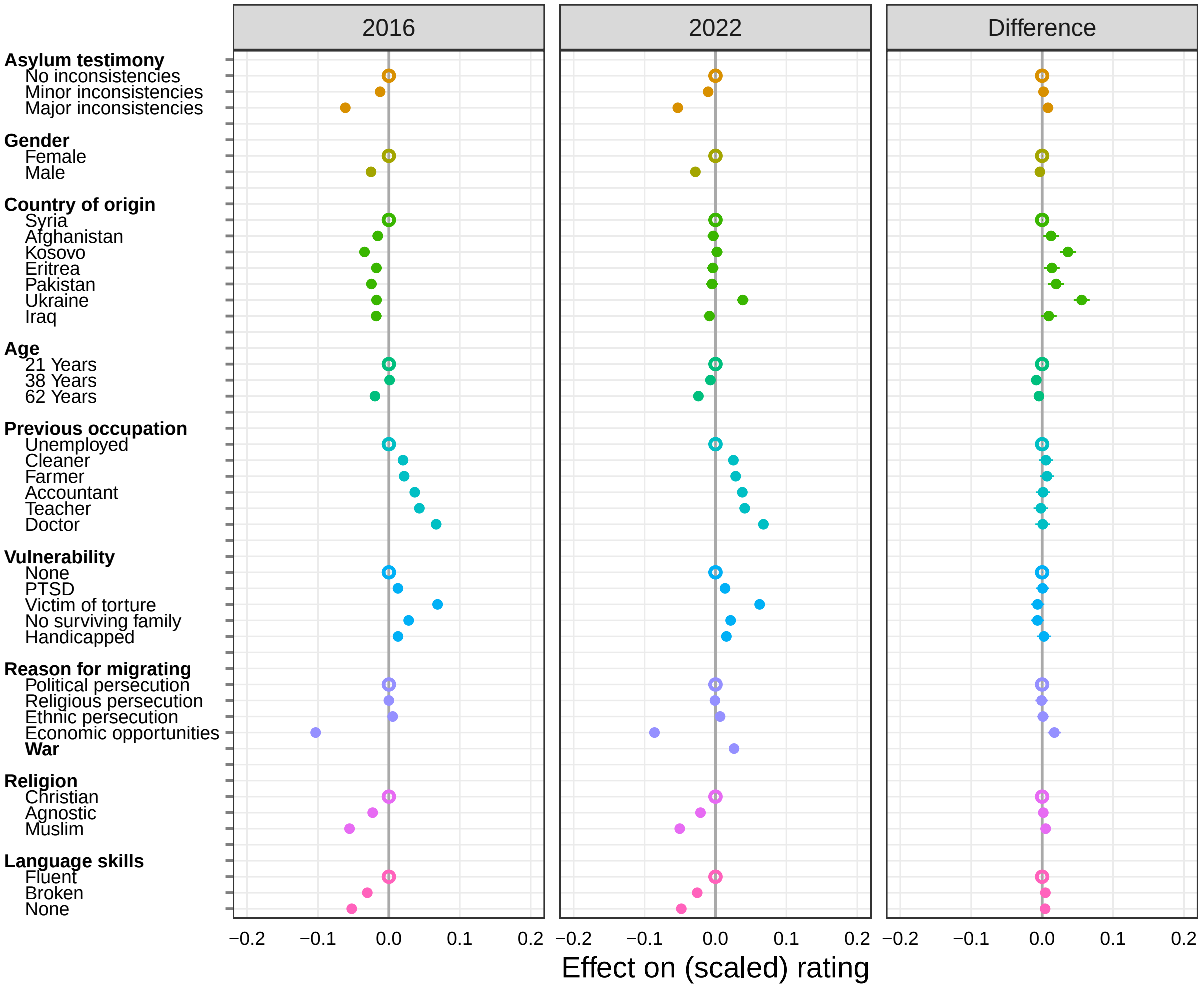

Figure S12: Effects of asylum-seeker attributes on the probability of respondent acceptance (using Binary Rating outcome variable), results from 2016, 2022, and difference

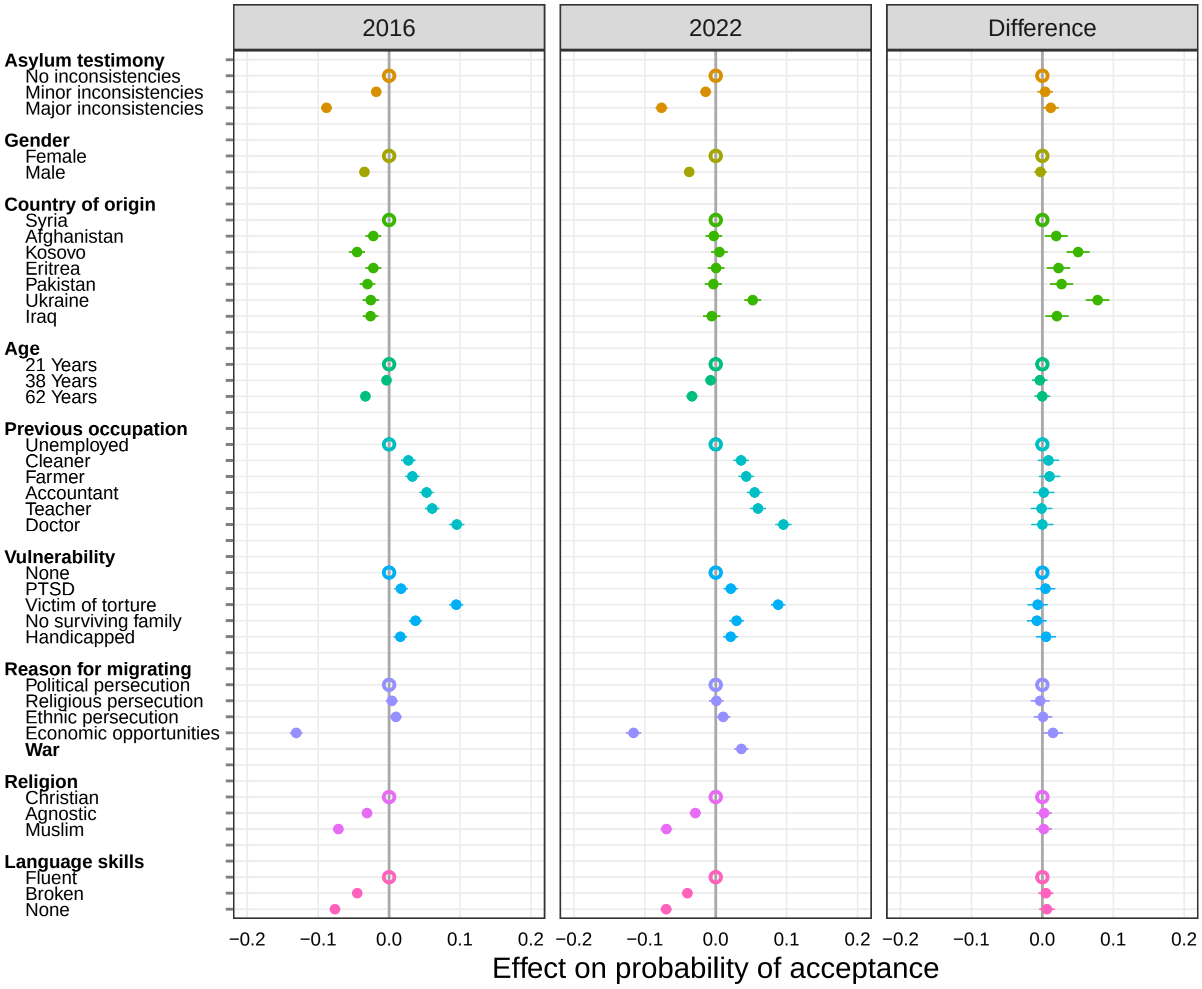

Figure S13: Effects of asylum-seeker attributes on the probability of respondent choice, by Reason for Migrating (War vs. not War), results from 2022

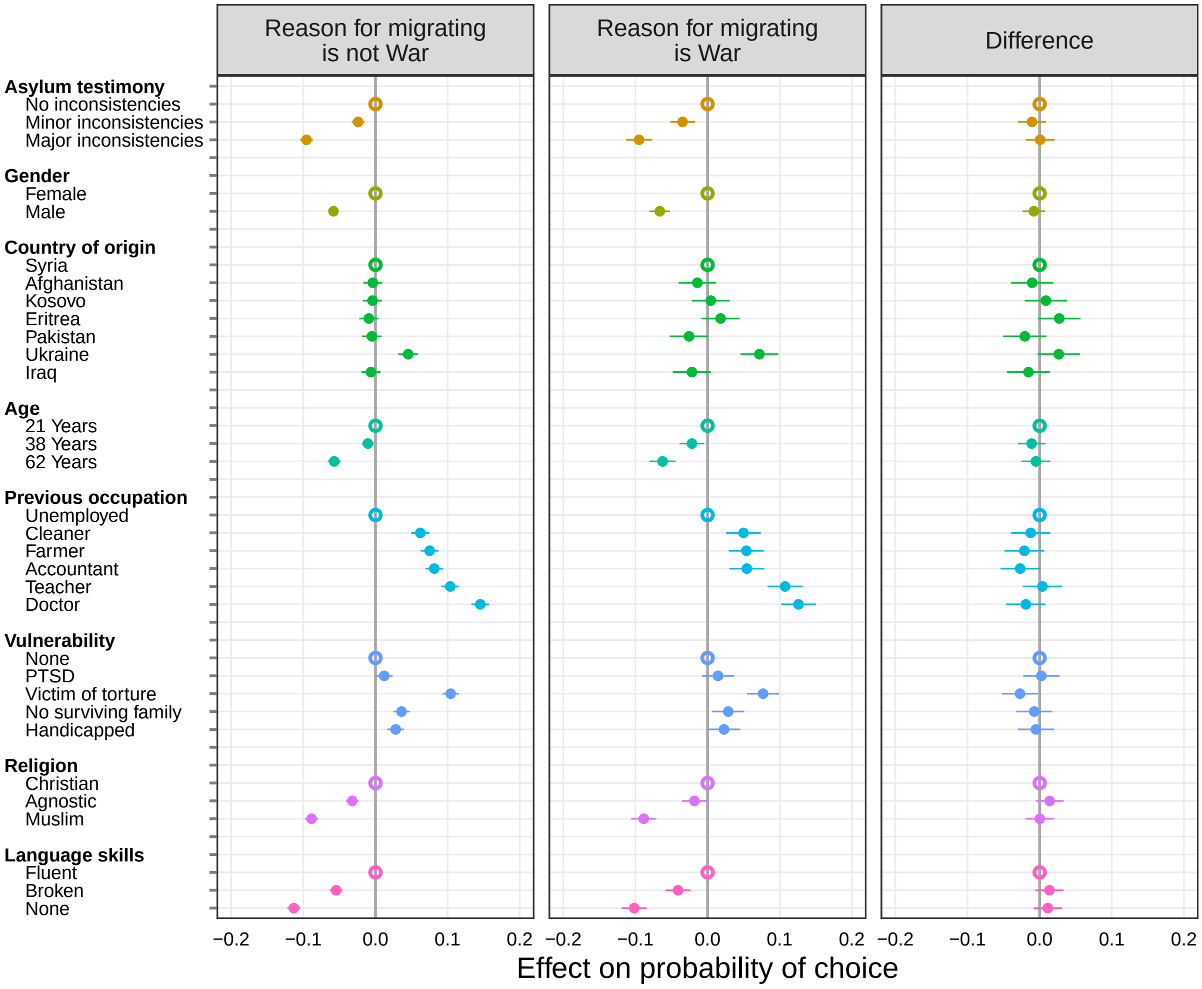

Figure S14: Effects of asylum-seeker attributes on the probability of respondent choice, by country, results from 2022

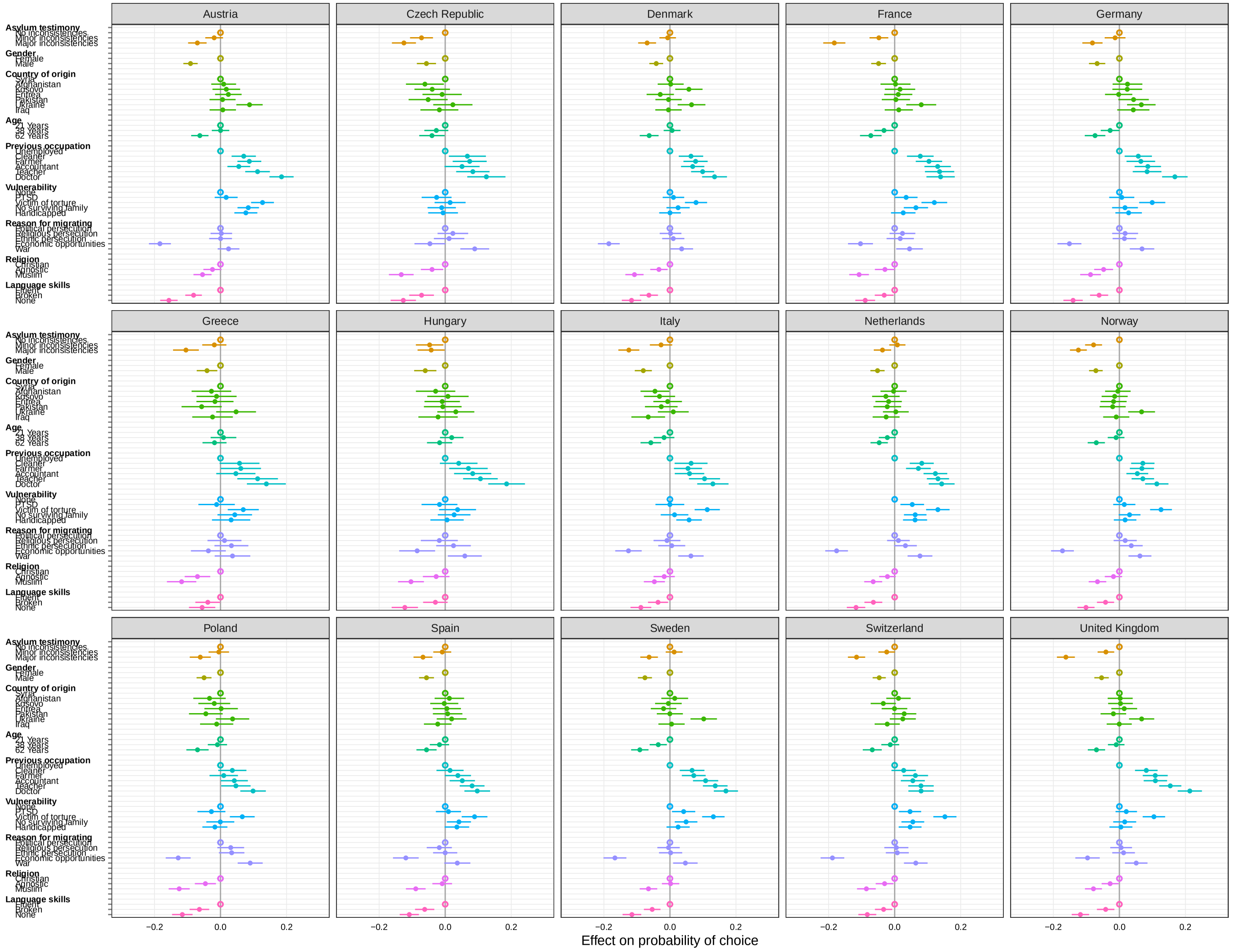

Figure S15: Effects of asylum-seeker attributes on the probability of respondent choice, by country, results from 2016

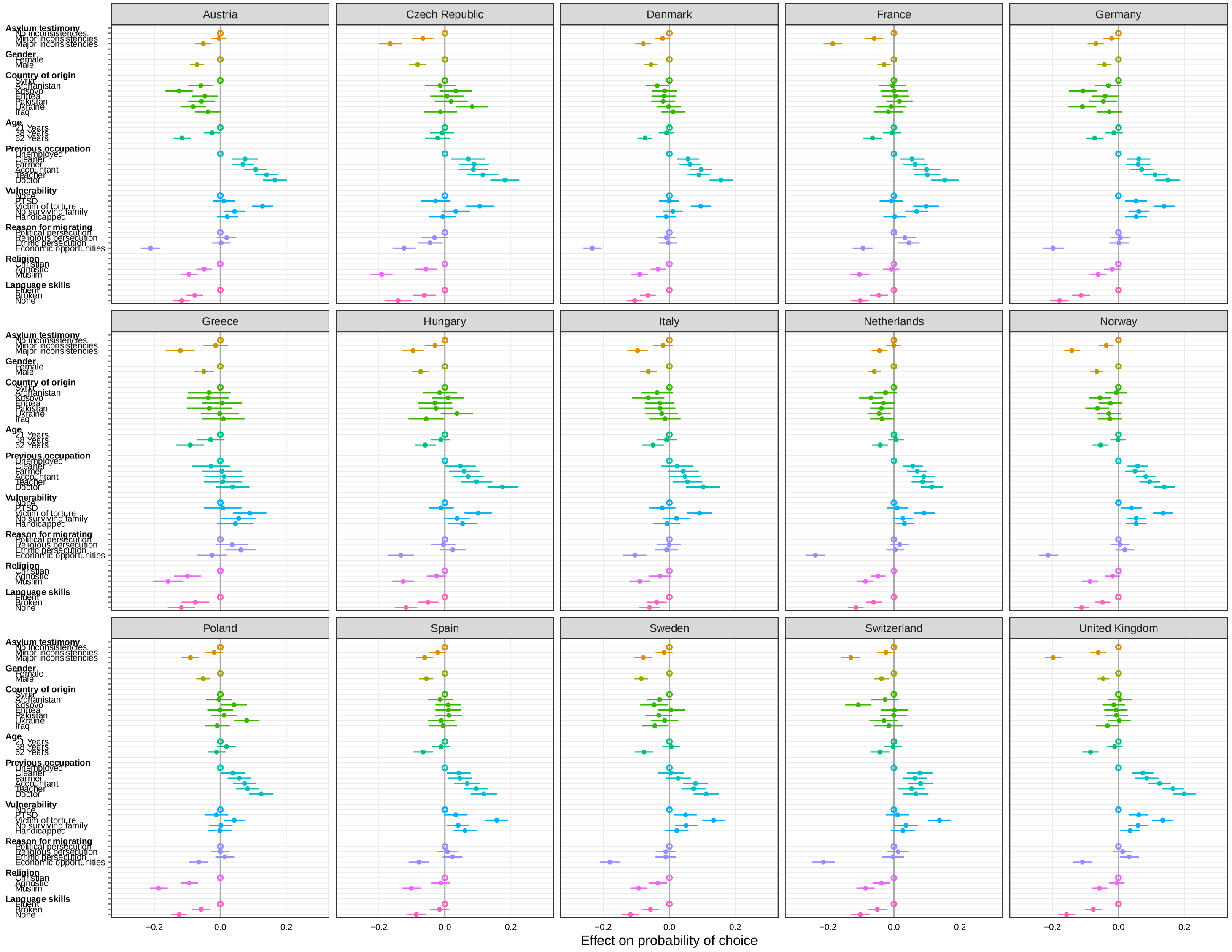

Figure S16: Difference in effects of asylum-seeker attributes on the probability of respondent choice (2022 vs. 2016), by country

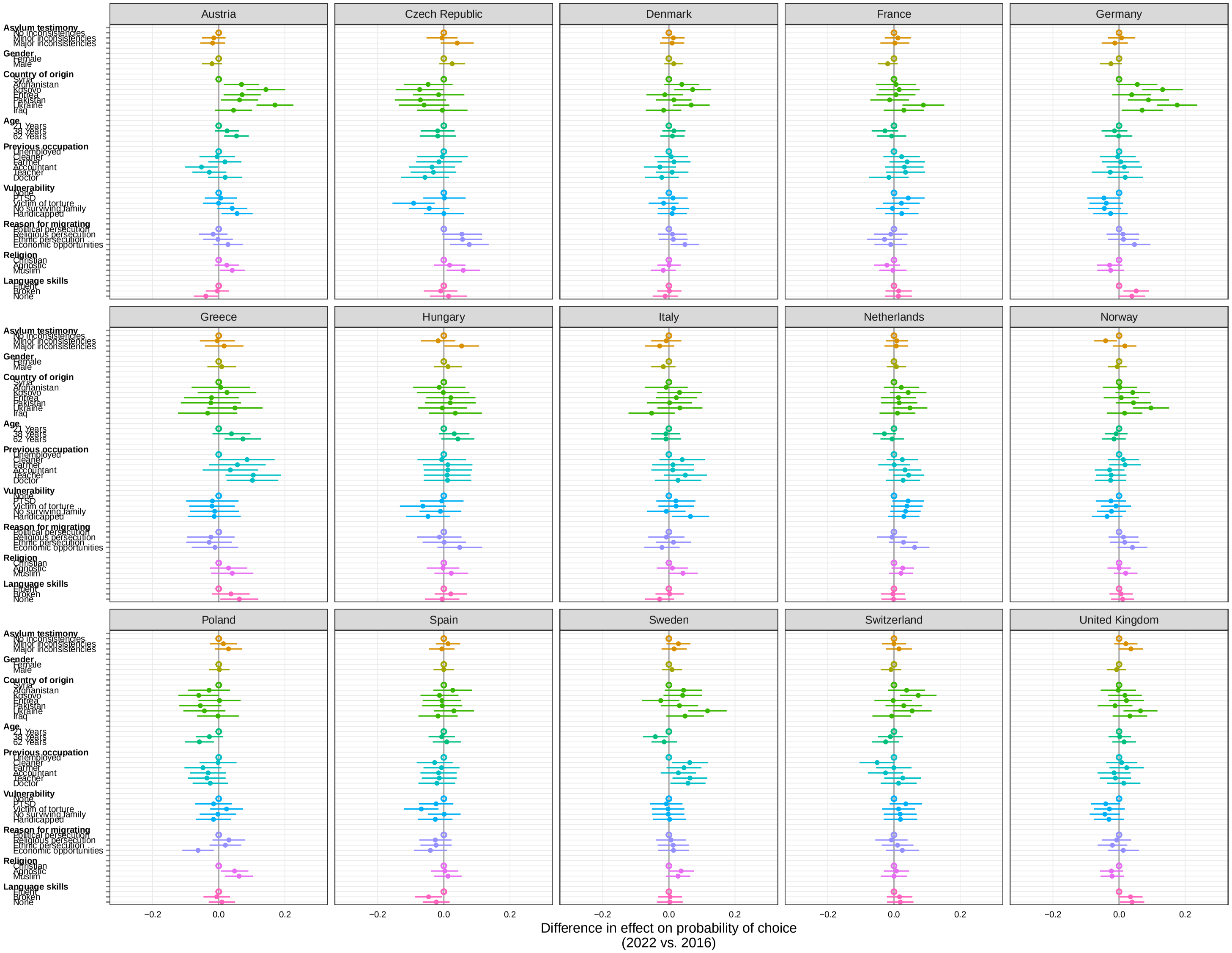

Figure S17: Effects of asylum-seeker attributes on the probability of respondent choice, for respondents from countries where the number of registered refugees from Ukraine (i.e. refugees from Ukraine registered for Temporary Protection or similar national protection schemes) is below/equal to the median vs. above the median within the sample of countries (and the difference), results from 2022

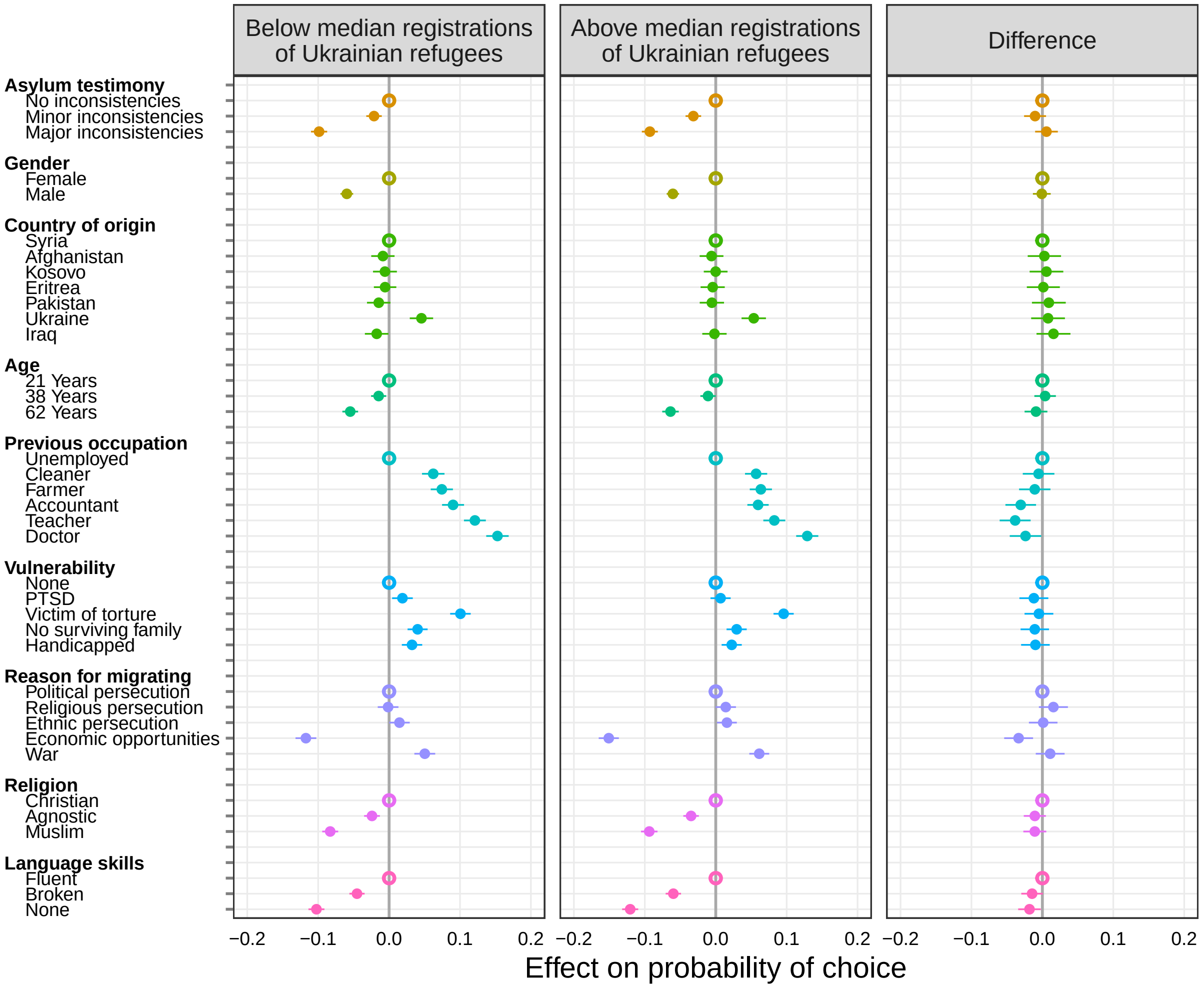

Figure S18: Effects of asylum-seeker attributes on the probability of respondent choice, by level of European Solidarity, results from 2022

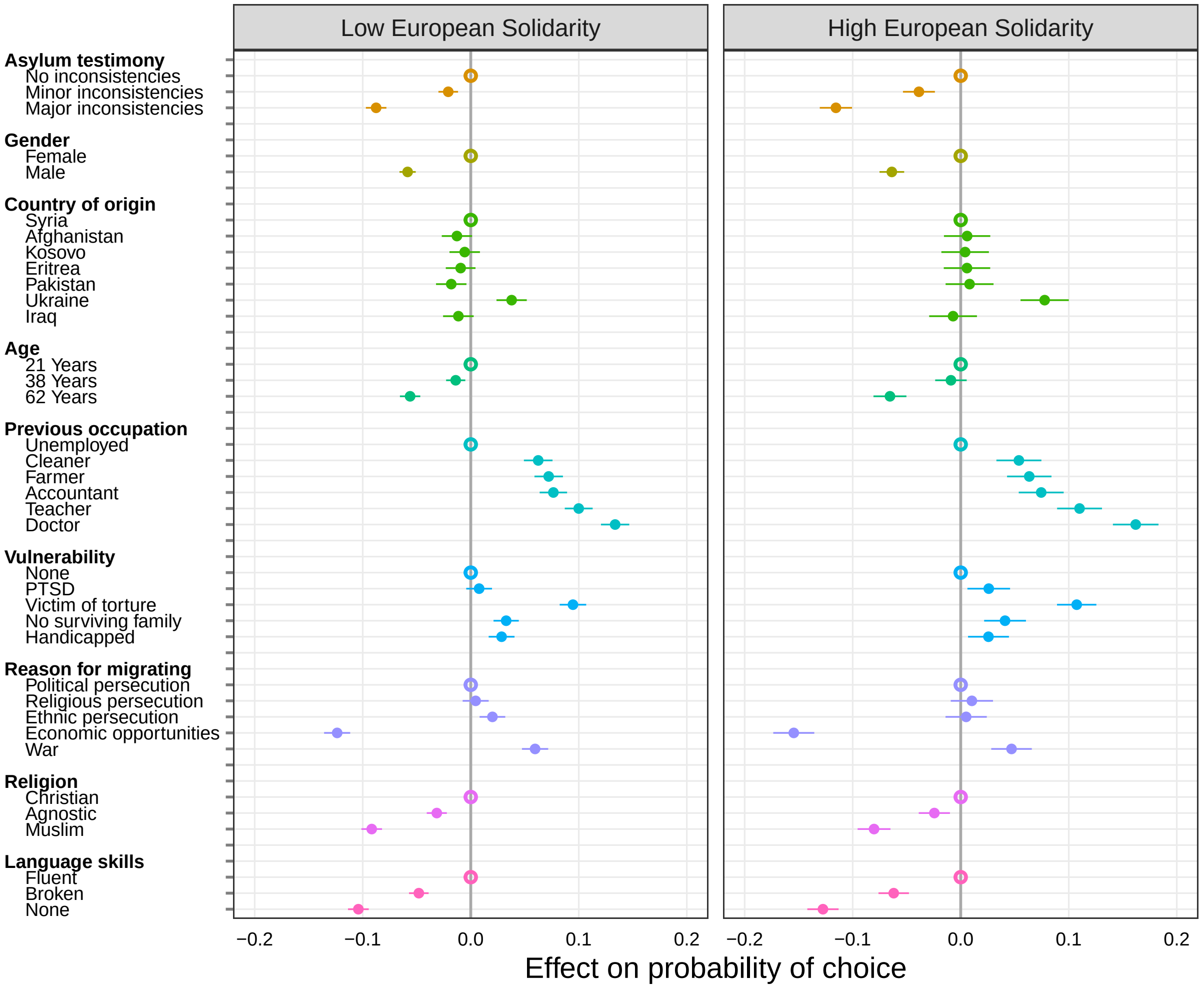

Figure S19: Influence of level of European Solidarity on effect of Ukraine country of origin (vs. all other countries) on the probability of respondent choice, results from 2022

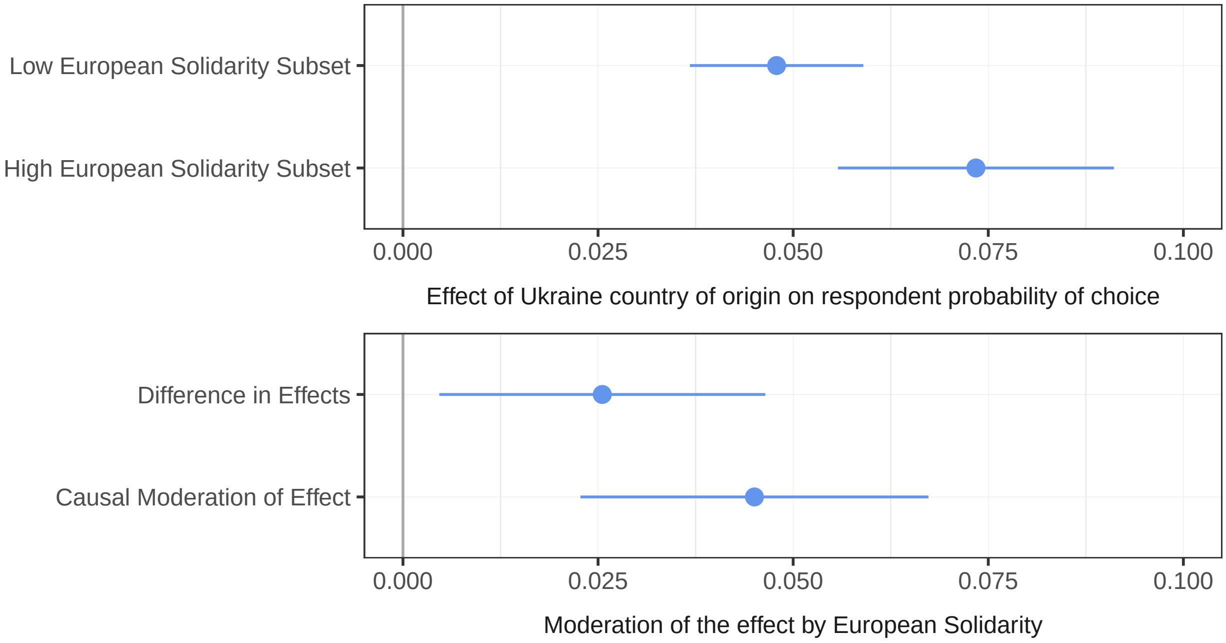

Figure S20: Support for increasing the number of people granted asylum in home country, results from 2016, 2022, and difference, by left-right ideology

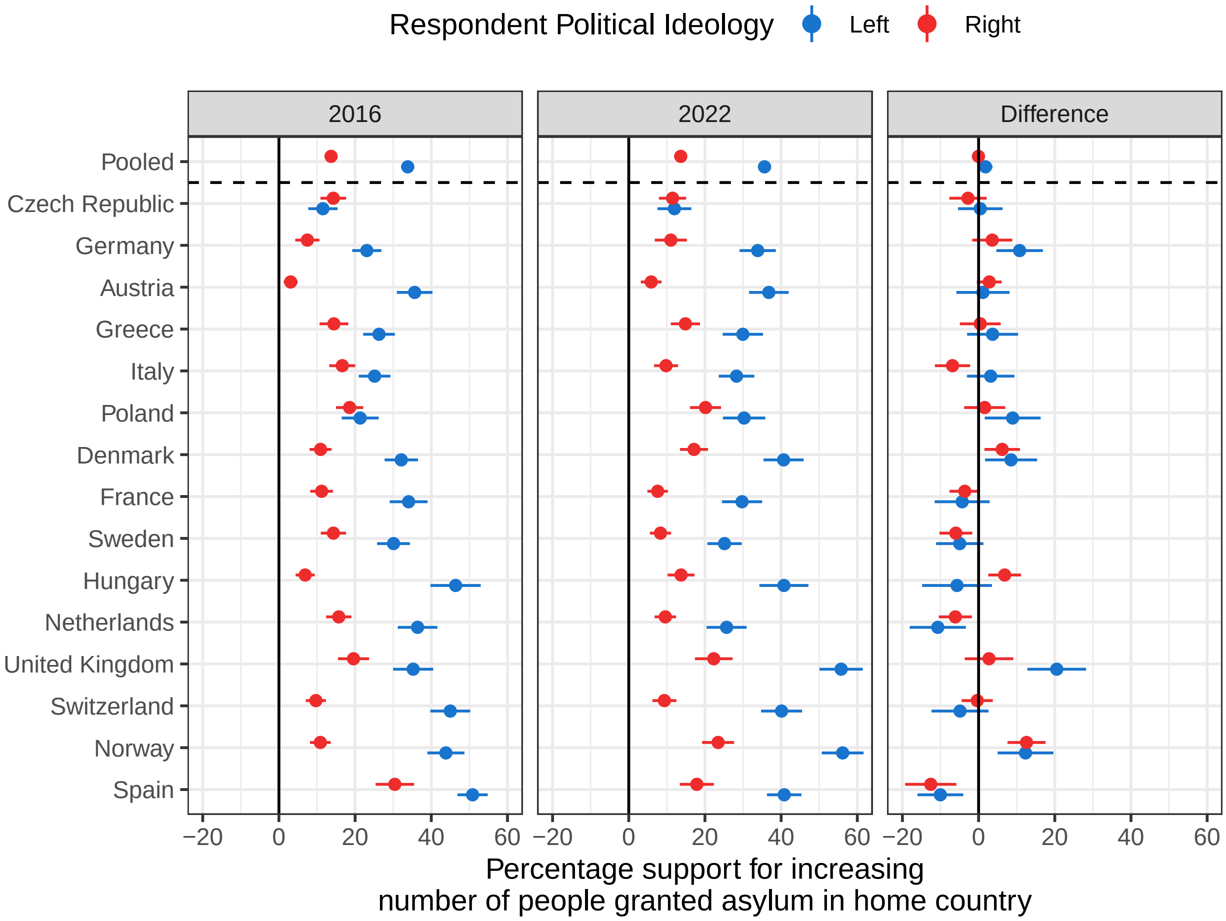

Figure S21: Support for increasing the number of people granted asylum in Europe, results from 2016, 2022, and difference, by left-right ideology

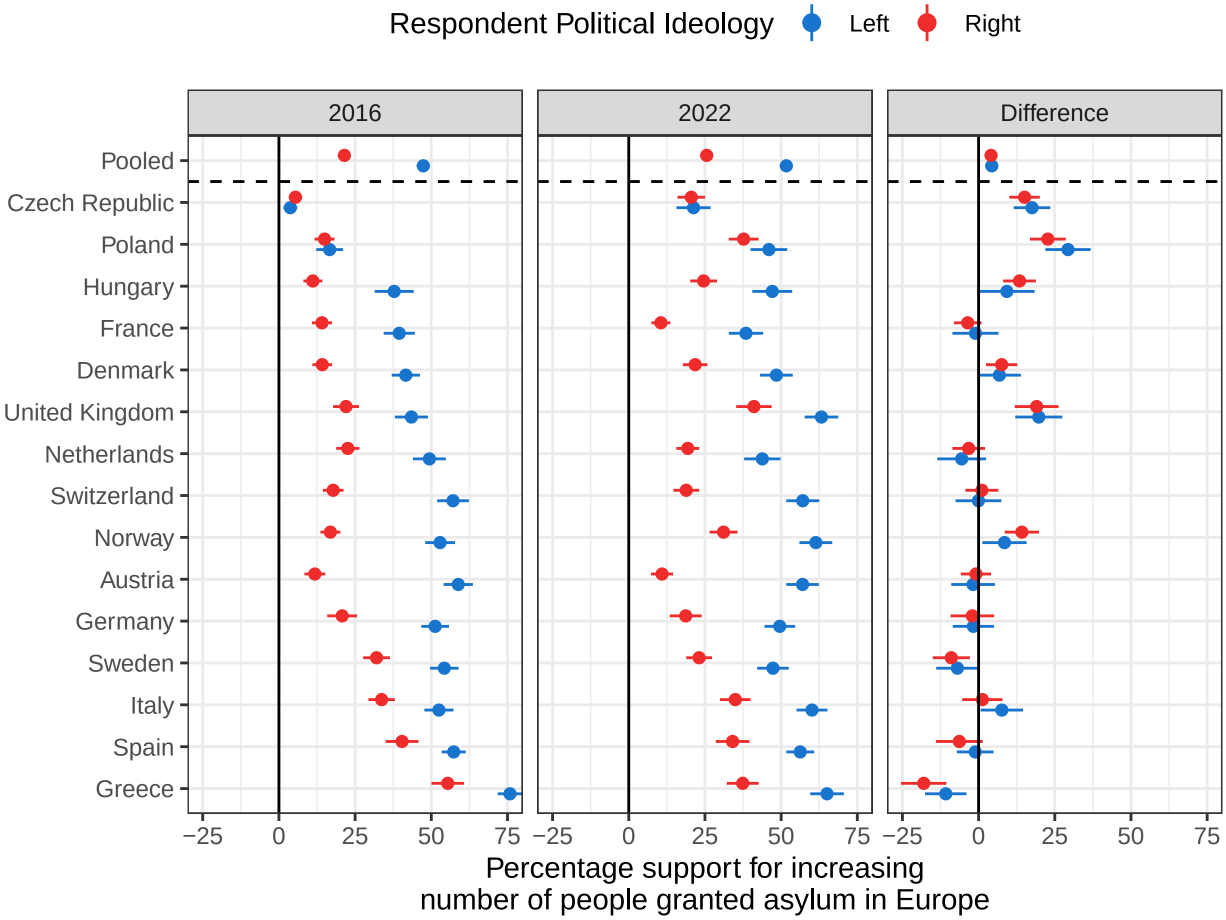

Figure S22: Effects of asylum-seeker attributes on the probability of respondent choice, by level of Russia Threat perception, results from 2022

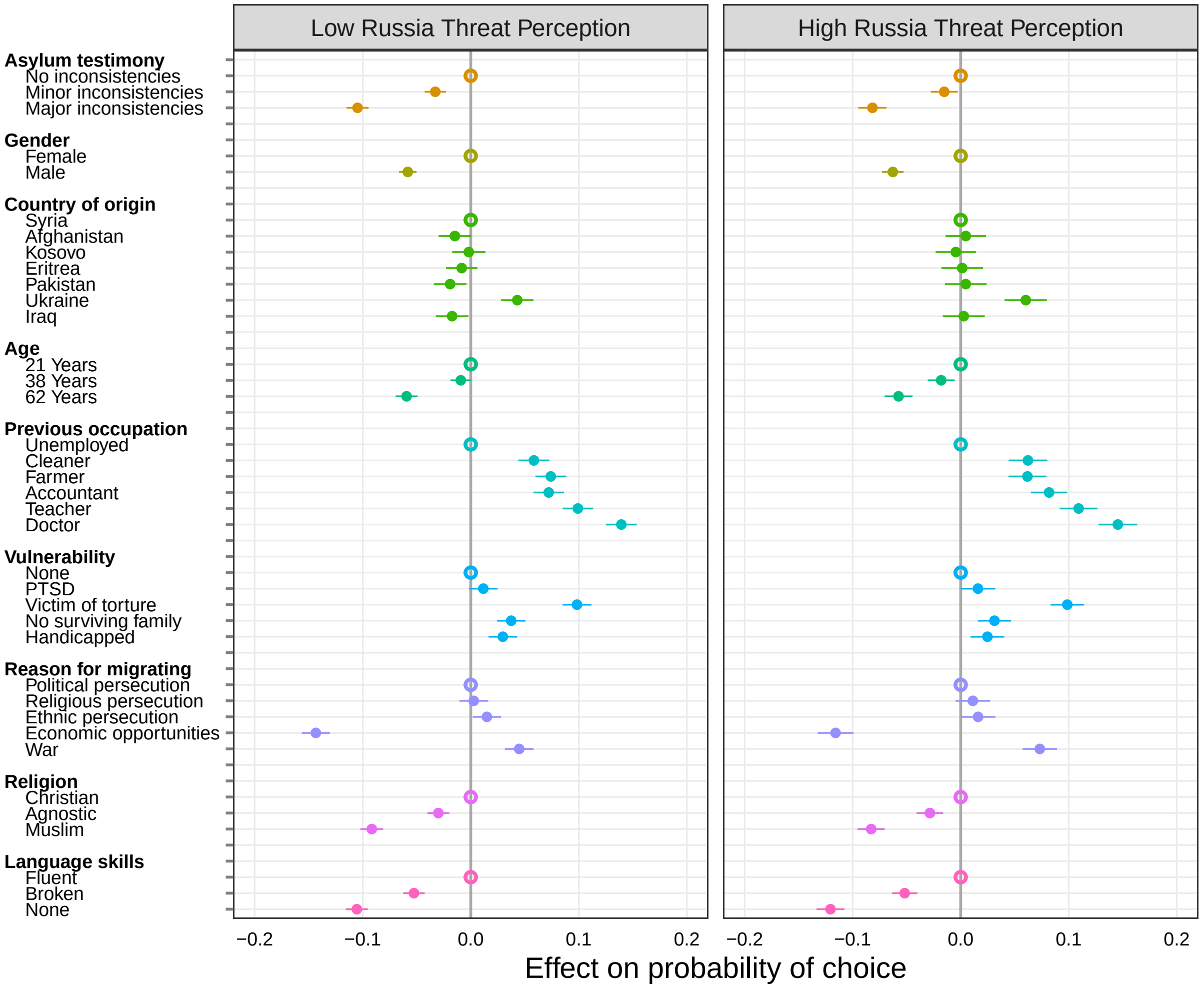

Figure S23: Influence of level of Russia Threat perception on effect of Ukraine country of origin (vs. all other countries) on the probability of respondent choice, results from 2022

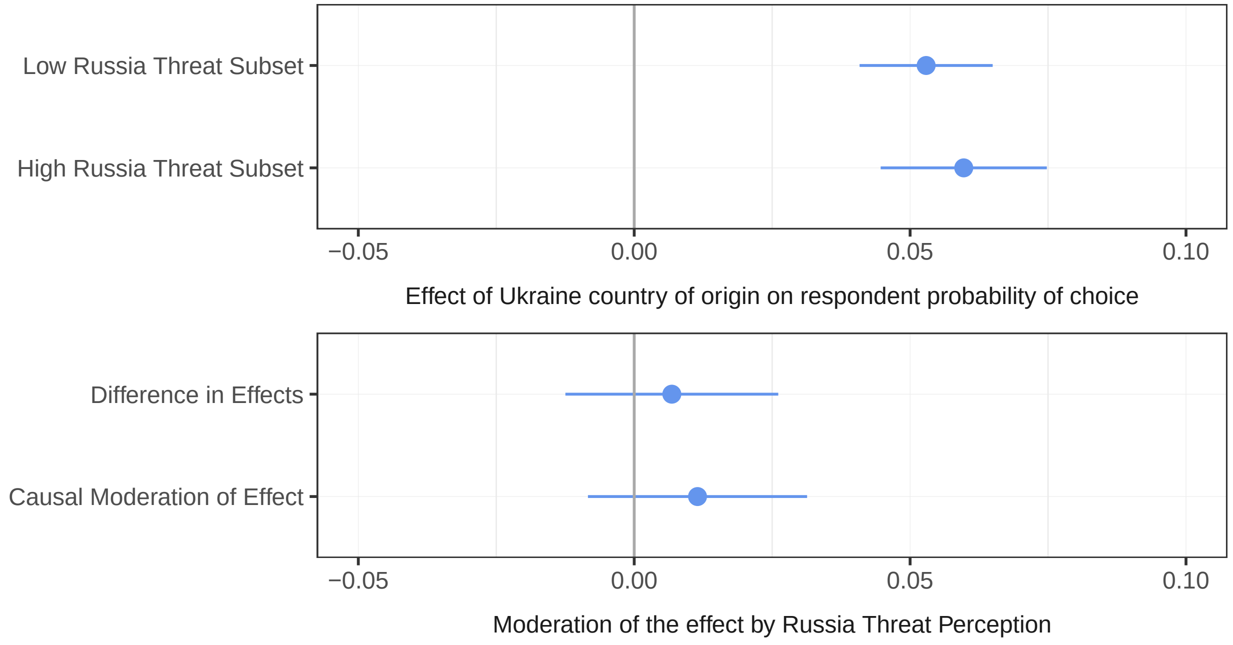

Figure S24: Effects of asylum-seeker attributes on the probability of respondent choice, by frame, results from 2022

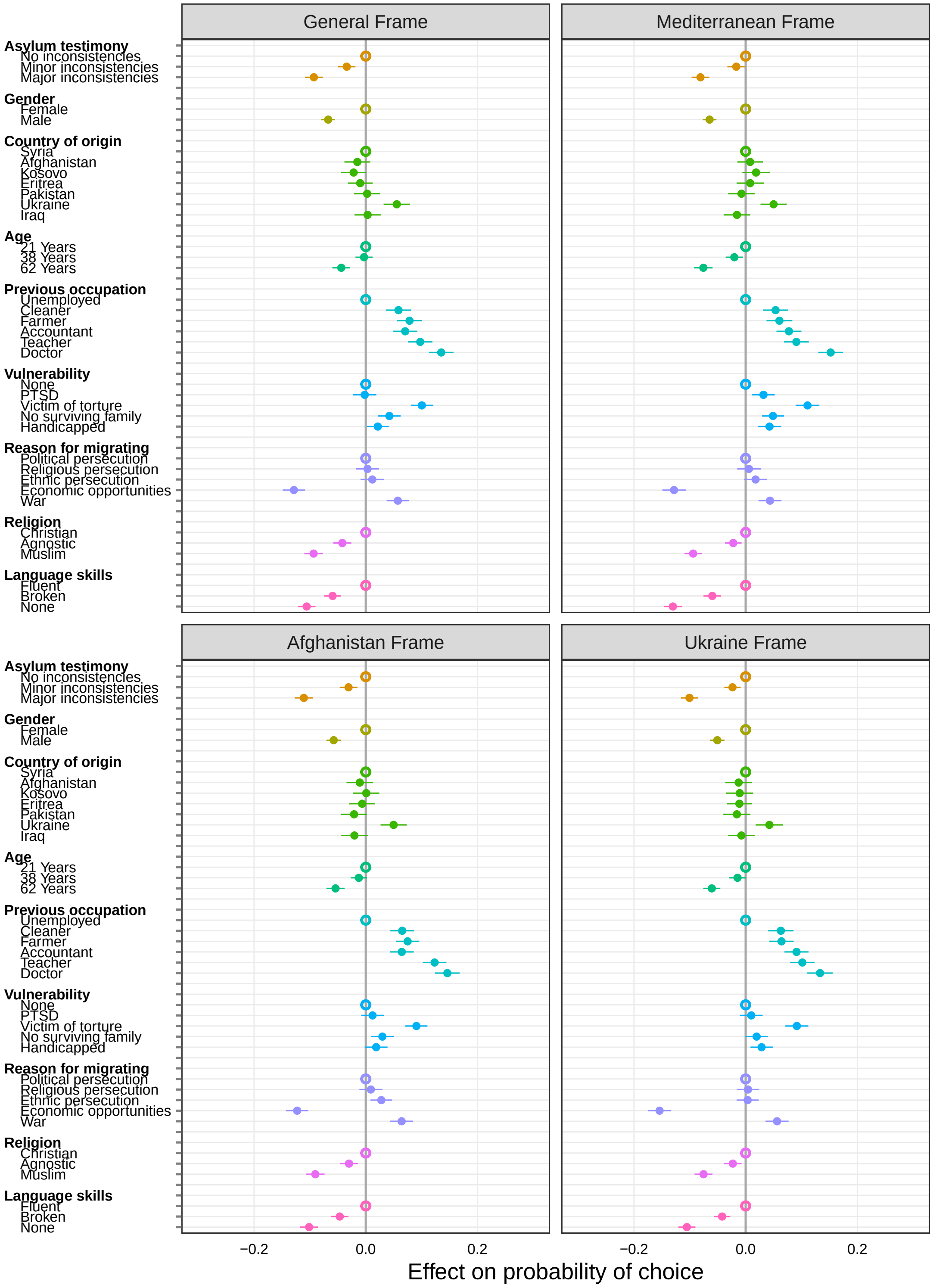

Figure S25: Distribution of p-values for interactions between the AMCEs and frames

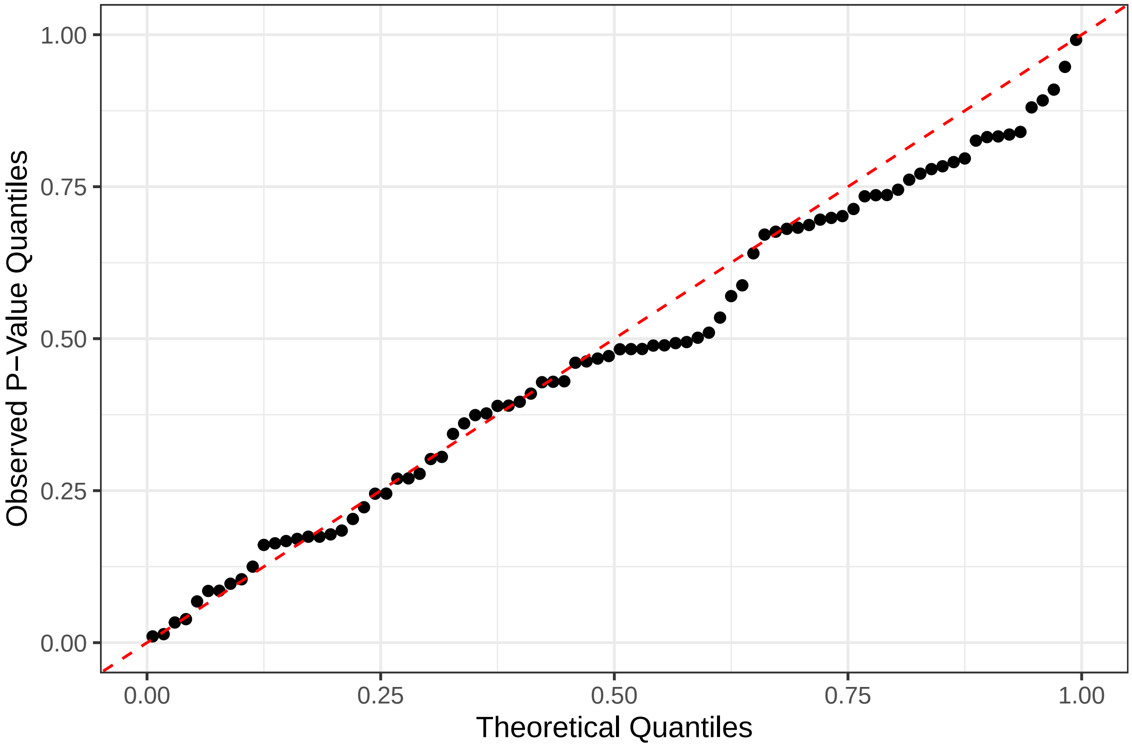

Figure S26: Effects of frames on feeling toward compatriots and asylum seekers from different countries of origin

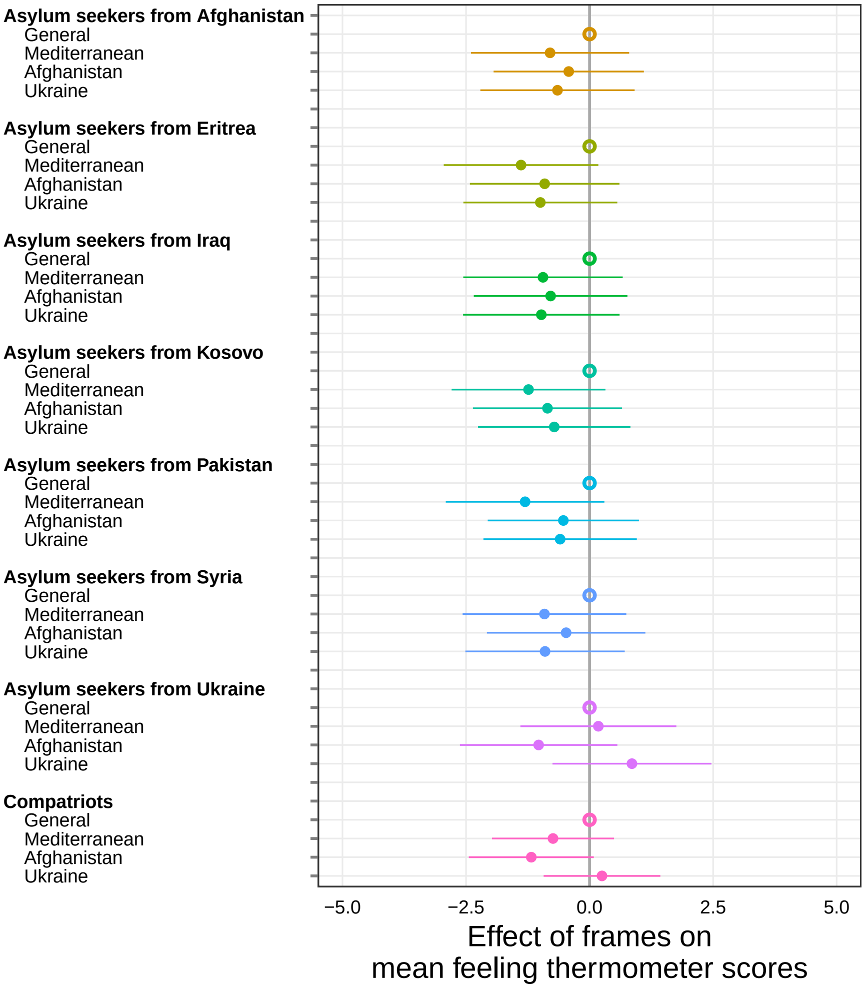

Figure S27: Support for immigration over time: Pooled Sample from European Social Survey (N=236,257 left panel and N=235,146 right panel)

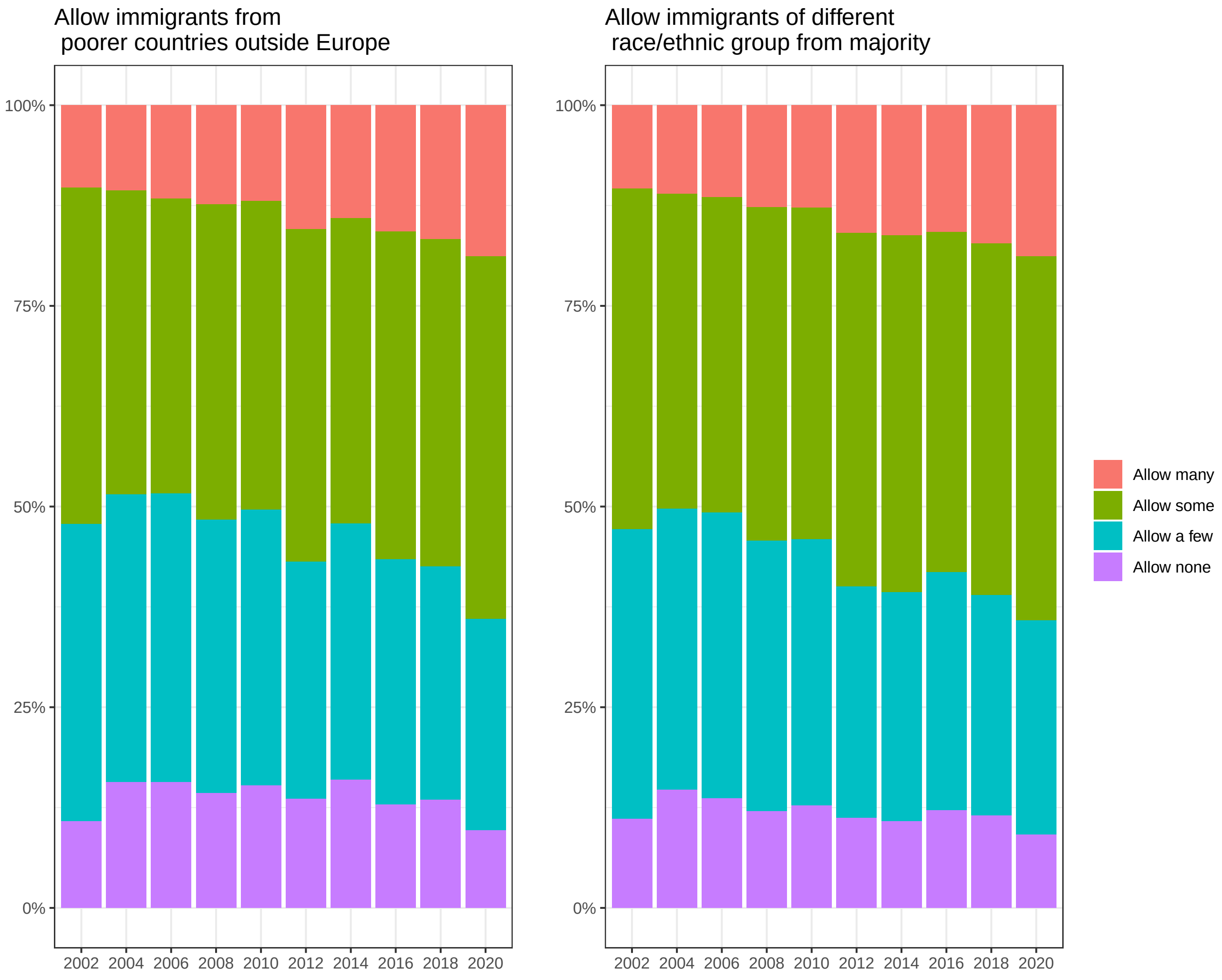

Figure S28: Support for immigration over time: Pooled Sample from European Social Survey with Countries that appear in all ten waves (N=155,309 left panel and N=140,018 right panel)

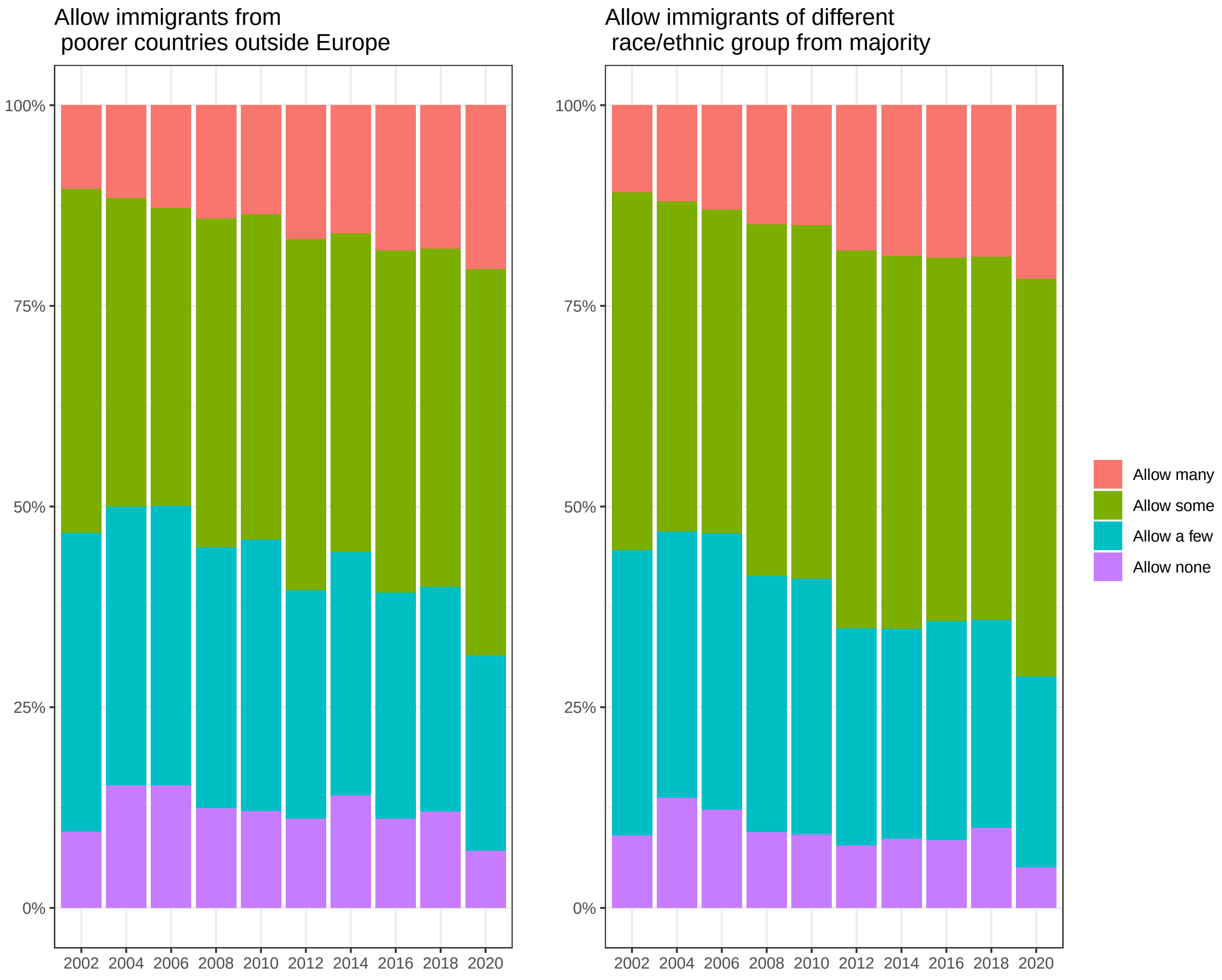

Figure S29: Support for immigration over time by country: European Social Survey

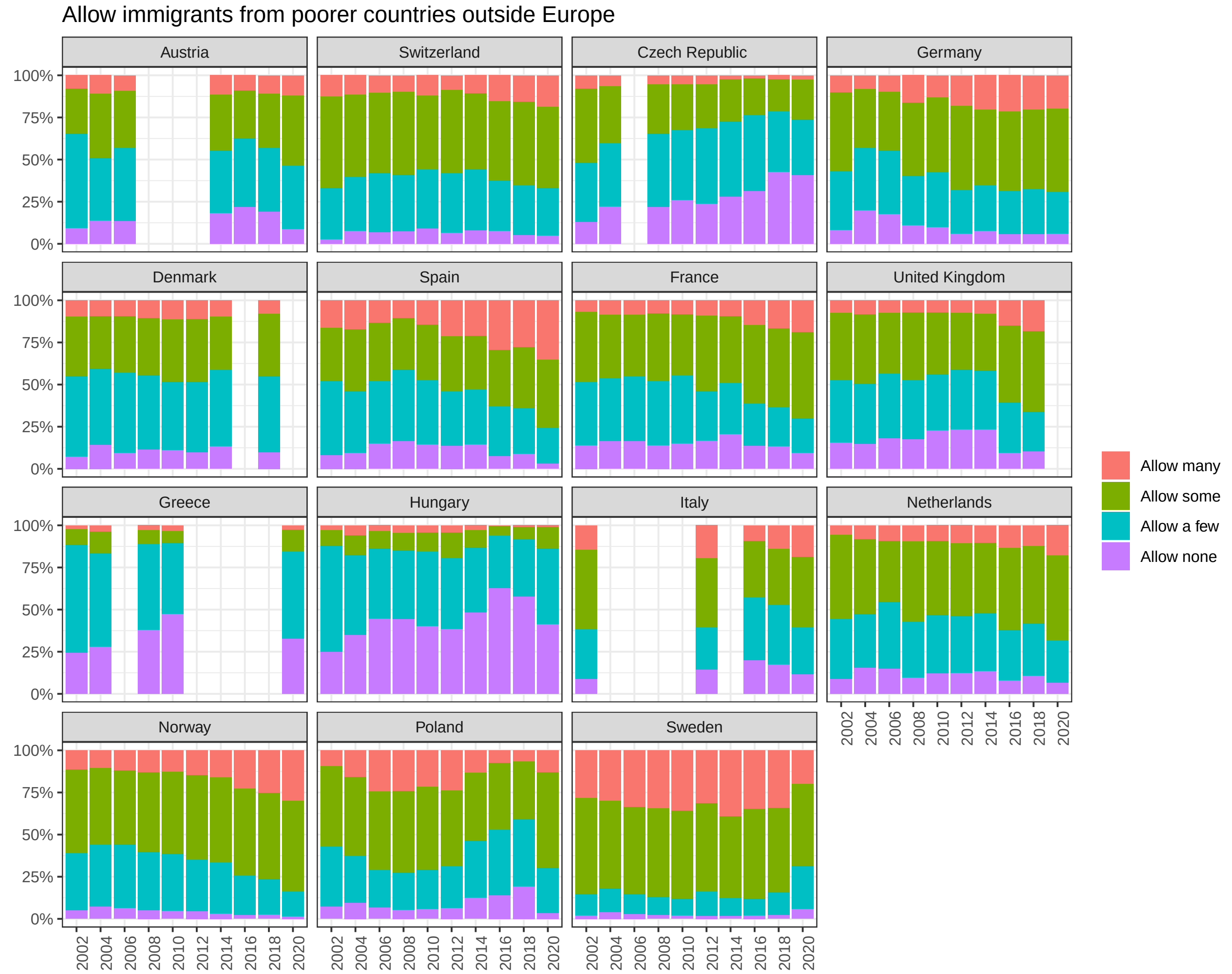

Figure S30: Support for immigration over time by country: European Social Survey

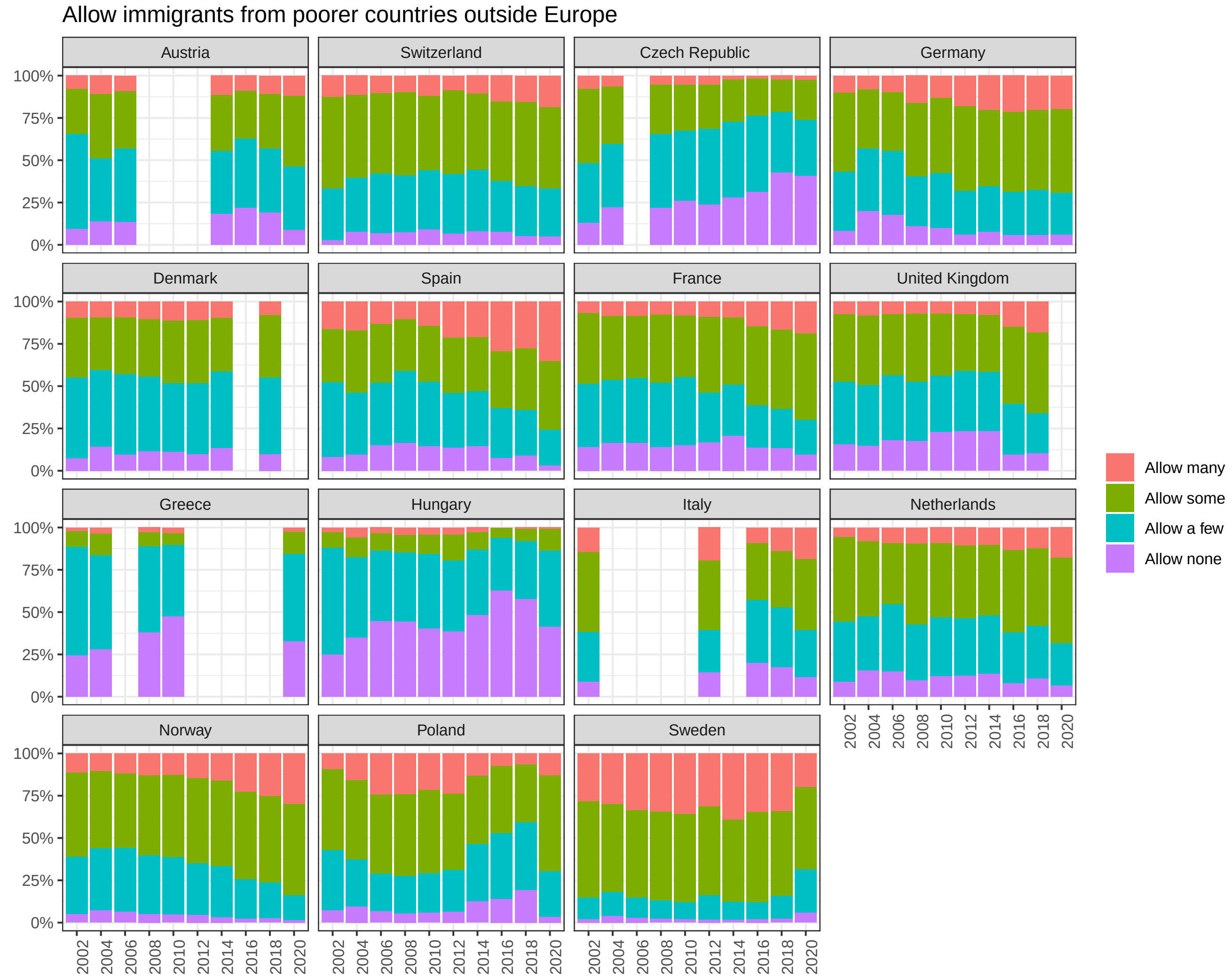

Figure S31: Probability calibration for boosted tree models predicting respondent choice of asylum-seeker conjoint profiles (as a function of the attributes)

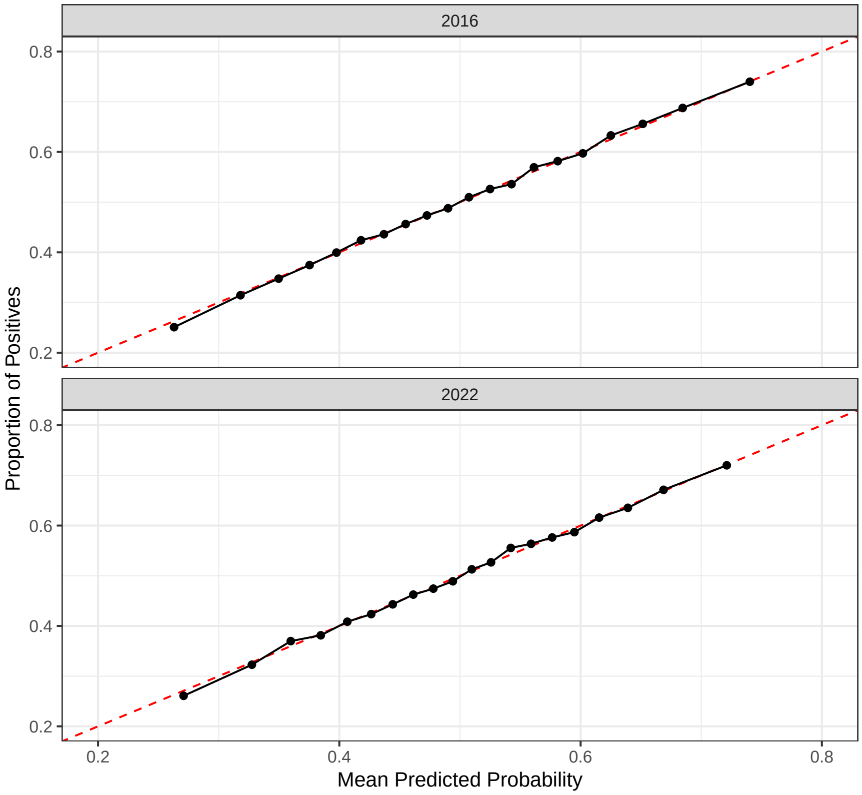

Figure S32: Distribution of choice probabilities over all possible attribute-level combinations, using boosted tree models

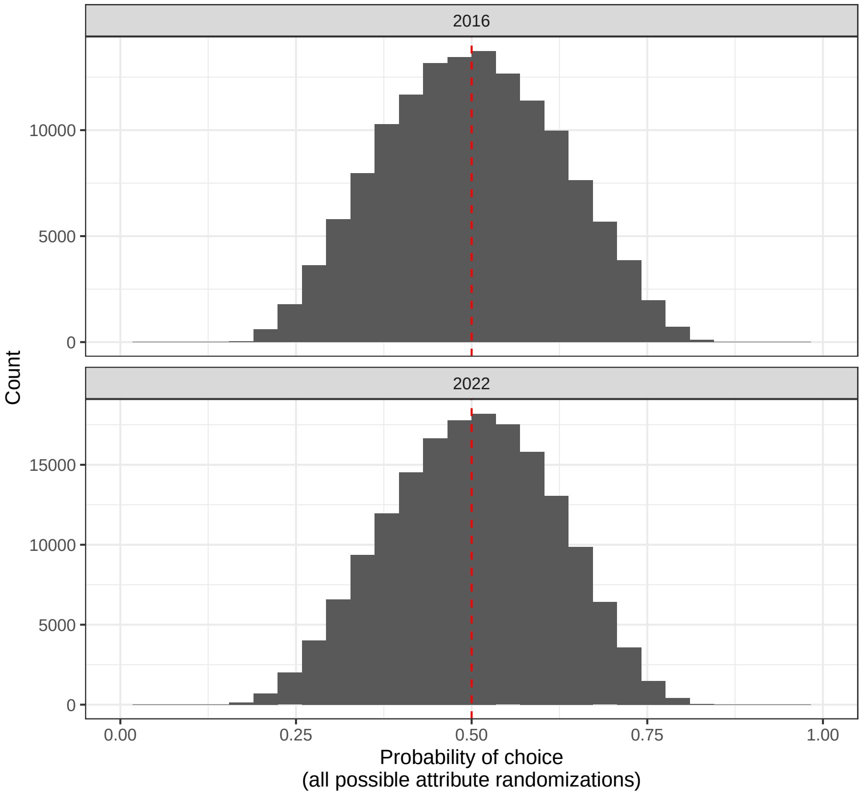

Figure S33: Distribution of the number of asylum-seeker profiles accepted by respondents, based on Binary Rating outcome (i.e. Rating of greater than 4 out of 7)

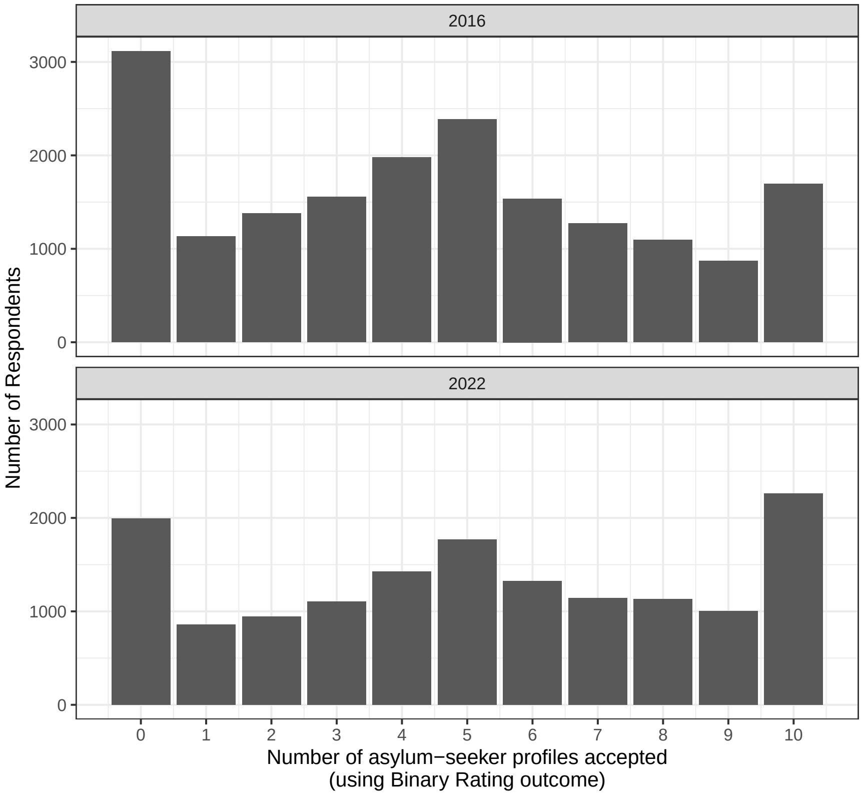

SI References

33. Roger Tourangeau, Frederick G Conrad, and Mick P Couper. *The Science of Web Surveys*. Oxford University Press, 2013.

34. Jens Hainmueller. Entropy balancing for causal effects: A multivariate reweighting method to produce balanced samples in observational studies. *Political Analysis*, 20(1):25–46, 2011.

35. Jens Hainmueller, Daniel J Hopkins, and Teppei Yamamoto. Causal inference in conjoint analysis: Understanding multidimensional choices via stated preference experiments. *Political Analysis*, 22(1):1–30, 2014.

36. Greg M Allenby, Nino Hardt, and Peter E Rossi. Economic foundations of conjoint analysis. In *Handbook of the Economics of Marketing*, volume 1, pages 151–192. Elsevier, 2019.

37. Kirk Bansak, Jens Hainmueller, Daniel J Hopkins, and Teppei Yamamoto. *Cambridge Handbook of Advances in Experimental Political Science*, chapter Conjoint Survey Experiments, pages 19–41. Cambridge University Press, 2021.

38. Jens Hainmueller, Dominik Hangartner, and Teppei Yamamoto. Validating vignette and conjoint survey experiments against real-world behavior. *Proceedings of the National Academy of Sciences*, 112(8):2395–2400, 2015.

39. Yusaku Horiuchi, Zachary Markovich, and Teppei Yamamoto. Does conjoint analysis mitigate social desirability bias? *Political Analysis*, 30(4):535–549, 2022.

40. Kirk Bansak, Jens Hainmueller, and Dominik Hangartner. How economic, humanitarian, and religious concerns shape European attitudes toward asylum seekers. *Science*, 354(6309):217–222, 2016.

41. Staatssekretariat für Migration SEM. Handbuch asyl und rückkehr: Artikel c6 befragung zur person (https://www.sem.admin.ch/dam/data/sem/asyl/verfahren/hb/c/hb-c6-d.pdf). Technical report, Abteilung Empfangs- und Verfahrenszentren, Direktionsbereich Asyl, Staatssekretariat für Migration SEM, 00 2015.

42. Staatssekretariat für Migration SEM. Handbuch asyl und ruckkehr, artikel c1, abteilung empfangs- und verfahrenszentren (https://www.sem.admin.ch/dam/data/sem/asyl/verfahren/hb/c/hb-c1-d.pdf). Technical report, Abteilung Empfangs- und Verfahrenszentren, Direktionsbereich Asyl, Staatssekretariat für Migration SEM, 00 2016.

43. Jens Hainmueller and Daniel J Hopkins. The hidden American immigration consensus: A conjoint analysis of attitudes toward immigrants. *American Journal of Political Science*, 59(3):529–548, 2015.

44. Kirk Bansak. Estimating causal moderation effects with randomized treatments and non-randomized moderators. *Journal of the Royal Statistical Society: Series A (Statistics in Society)*, 184(1):65–86, 2021.
