# Supplementary material for: Europeans’ support for refugees of varying background is stable over time
Source: Nature. 2023 Aug 9;620(7975):849–54. doi: 10.1038/s41586-023-06417-6 (PMC10447233; doi:10.1038/s41586-023-06417-6)
Supplement: Supplementary file 2 — Reporting Summary [file 41586_2023_6417_MOESM2_ESM.pdf]

nature portfolio

Corresponding author(s): Dominik Hangartner

Last updated by author(s): 26/06/2023

Reporting Summary

Nature Portfolio wishes to improve the reproducibility of the work that we publish. This form provides structure for consistency and transparency in reporting. For further information on Nature Portfolio policies, see our Editorial Policies and the Editorial Policy Checklist.

Statistics

For all statistical analyses, confirm that the following items are present in the figure legend, table legend, main text, or Methods section.

n/a	Confirmed	
☐	☒	The exact sample size (*n*) for each experimental group/condition, given as a discrete number and unit of measurement
☐	☒	A statement on whether measurements were taken from distinct samples or whether the same sample was measured repeatedly
☐	☒	The statistical test(s) used AND whether they are one- or two-sided<br>*Only common tests should be described solely by name; describe more complex techniques in the Methods section.*
☐	☒	A description of all covariates tested
☐	☒	A description of any assumptions or corrections, such as tests of normality and adjustment for multiple comparisons
☐	☒	A full description of the statistical parameters including central tendency (e.g. means) or other basic estimates (e.g. regression coefficient) AND variation (e.g. standard deviation) or associated estimates of uncertainty (e.g. confidence intervals)
☐	☒	For null hypothesis testing, the test statistic (e.g. *F*, *t*, *r*) with confidence intervals, effect sizes, degrees of freedom and *P* value noted<br>*Give P values as exact values whenever suitable.*
☒	☐	For Bayesian analysis, information on the choice of priors and Markov chain Monte Carlo settings
☒	☐	For hierarchical and complex designs, identification of the appropriate level for tests and full reporting of outcomes
☐	☒	Estimates of effect sizes (e.g. Cohen's *d*, Pearson's *r*), indicating how they were calculated

Our web collection on statistics for biologists contains articles on many of the points above.

Software and code

Policy information about availability of computer code

Data collection	To collect the survey data, we used Qualtrics software, Version 2022.
Data analysis	For all analysis, we used R Studio, Version 2022.07.2.

For manuscripts utilizing custom algorithms or software that are central to the research but not yet described in published literature, software must be made available to editors and reviewers. We strongly encourage code deposition in a community repository (e.g. GitHub). See the Nature Portfolio guidelines for submitting code & software for further information.

Data

Policy information about availability of data

All manuscripts must include a data availability statement. This statement should provide the following information, where applicable:

- Accession codes, unique identifiers, or web links for publicly available datasets
- A description of any restrictions on data availability
- For clinical datasets or third party data, please ensure that the statement adheres to our policy

All replication code and data are publicly available at the dedicated Harvard Dataverse: https://osf.io/jd8n3/

March 2021

Human research participants

Policy information about studies involving human research participants and Sex and Gender in Research.

Reporting on sex and gender	Gender was considered in the study design in two ways. First, gender is one of the randomized attributes of the hypothetical profiles in the conjoint analysis. Second, the sampling process ensured that our respondent sample was balanced in terms of gender (which was self-reported in the survey by the respondent). In addition, gender is one of the variables upon which we reweighted our samples to match the demographic margins for each country. This self-reported gender variable is included in the data. The following is the self-reported gender breakdown (n).<br>Female: 15080<br>Male: 14722
Population characteristics	The detailed descriptive statistics for gender, age, income and political ideology for our respondent samples are provided in Table S2.
Recruitment	For each country and survey wave, the survey firm Respondi and its local partners sampled eligible voters from their online panel. Respondi recruits new panelists for its panel from the general population, mostly through online channels and to a lesser extent through computer-assisted telephone interviews (CATI). After completing the enrollment interview, Respondi invites panelists to participate in several surveys (like ours), for which they are compensated. The same sampling mechanism was used for both our 2016 and 2022 survey waves, to maximize the comparability of the samples. In addition to Respondi's standard recruitment processes, the recruitment of panelists for both waves of our survey also employed age and gender quotas to roughly match the population margins for each of the countries in our study. Post-stratification weights were also constructed to account for remaining imbalances, as explained below.<br><br>There are, as with all surveys, potential concerns about representativeness of the sample and the subsequent risk of bias in our estimates. In both survey waves in 2022 and 2016 our sample is somewhat skewed towards more educated and younger respondents. This skew is common in surveys with online panels. To address these imbalances we follow the design described in our pre-analysis plan and match our sample to the demographic margins in each country using entropy balancing. In particular, we adjust the sample so that it matches the distribution of each country on age, gender, and educational attainment. For gender we match on the % female on the population. For age we match on three age categories, including the % aged 18-39, the % aged 40-59, and the % aged 60+. For education we match on three categories of highest educational attainment, including the % below upper secondary education, the % with upper secondary or post-secondary non-tertiary education, and the % with tertiary education. We calculated the population margins using the most recently available statistics from the OECD at the time of each survey wave. For age and gender we used the OECD Population Statistics (http://stats.oecd.org/; file EAG_NEAC_29062022222713553). For education we use the table on the share of population by educational attainment in the OECD Education at a Glance database (http://stats.oecd.org/).<br><br>To investigate the likelihood of bias in our results resulting from any remaining imbalances, we conduct two robustness tests to probe the stability of our results. First, we find that unweighted results are very similar to the estimates weighted for each country's age, gender, and education distributions. We also conduct our analyses using a second set of weights that, in addition, take into account each country's political ideological distribution, measured on a standard 0-10 left-right ideology scale. Again, the results are very similar to the original weighted estimates. All of these analyses are reported in the manuscript, with full details provided in the SI.
Ethics oversight	Our 2016 survey was conducted according to the University of Zurich's policy for human subjects research and approved by Stanford University's Institutional Review Board (protocol ID: 34881). Our 2022 survey was approved by Stanford University's Institutional Review Board (protocol ID 34881) and ETH Zurich's Ethics Committee (protocol IRB00007709).

Note that full information on the approval of the study protocol must also be provided in the manuscript.

Field-specific reporting

Please select the one below that is the best fit for your research. If you are not sure, read the appropriate sections before making your selection.

☐ Life sciences ☒ Behavioural & social sciences ☐ Ecological, evolutionary & environmental sciences

For a reference copy of the document with all sections, see nature.com/documents/nr-reporting-summary-flat.pdf

Behavioural & social sciences study design

All studies must disclose on these points even when the disclosure is negative.

Study description	This study reports the results from a quantitative survey experiment.
Research sample	The research sample is vote-eligible citizens in 15 European countries: Austria, the Czech Republic, Denmark, France, Germany, Greece, Hungary, Italy, the Netherlands, Norway, Poland, Spain, Sweden, Switzerland, and the United Kingdom. In both survey waves in 2022 and 2016 our sample is somewhat skewed towards more educated and younger respondents. This skew is common in surveys with online panels. To address these imbalances we follow the design described in our pre-analysis plan and match our

	sample to the demographic margins in each country using entropy balancing. In particular, we adjust the sample so that it matches the distribution of each country on age, gender, and educational attainment. For gender we match on the % female on the population. For age we match on three age categories, including the % aged 18-39, the % aged 40-59, and the % aged 60+. For education we match on three categories of highest educational attainment, including the % below upper secondary education, the % with upper secondary or post-secondary non-tertiary education, and the % with tertiary education. We calculated the population margins using the most recently available statistics from the OECD at the time of each survey wave. For age and gender we used the OECD Population Statistics (http://stats.oecd.org/; file EAG_NEAC_29062022222713553). For education we use the table on the share of population by educational attainment in the OECD Education at a Glance database (http://stats.oecd.org/). In addition, we constructed a second set of weights that take into account each country's political ideological distribution, measured on a standard 0-10 left-right ideology scale. Full detail on the samples and summary statistics by country and year are provided in Tables S2 - S4 in the SI.
Sampling strategy	For each country and survey wave, the survey firm Respondi and its local partners sampled eligible voters from their online panel. Respondi recruits new panelists for its panel from the general population, mostly through online channels and to a lesser extent through computer-assisted telephone interviews (CATI). After completing the enrollment interview, Respondi invites panelists to participate in several surveys (like ours), for which they are compensated. The same sampling mechanism was used for both our 2016 and 2022 survey waves, to maximize the comparability of the samples. In addition to Respondi's standard recruitment processes, the recruitment of panelists for both waves of our survey also employed age and gender quotas to roughly match the population margins for each of the countries in our study. Post-stratification weights were also constructed to account for remaining imbalances, as explained below.<br><br>Sample size calculations were not performed. Our 2016 sample size was determined by the sample size (and power) of similar conjoint experiments that we conducted in the past research (in particular: Hainmueller, Hangartner, Yamamoto 2015, PNAS). Our 2022 sample size was determined to be similar to our 2016 sample size.
Data collection	Qualtrics online survey software was used to collect the data. Respondents took the survey at a time, place, and device of their own choosing.
Timing	The first survey wave was conducted between late February and early March 2016, the second wave between mid May and early June 2022.
Data exclusions	For all weighted analyses we drop 147 respondents from the 2016 survey wave and 120 respondents from the 2022 survey wave for whom weights cannot be constructed due to missing data on the covariates. These exclusions were pre-registered, as we had pre-registered weighted analyses.
Non-participation	We do not have access to information on how many respondents declined an invitation from Respondi to participate in our survey. For respondents who began the survey and were found eligible to take the survey, the percentage of respondents who completed the survey was 79% for our 2016 sample and 87% for our 2022 sample.
Randomization	We leveraged a fully randomized paired profiles conjoint design. Each respondent was presented with five pairs of profiles of hypothetical asylum seekers displayed side-by-side. The profiles described hypothetical asylum seekers with nine attributes, including the asylum seeker's age, proficiency in the host country language, previous occupation, religion, consistency of the asylum testimony, special vulnerabilities, country of origin, reason for migrating, and gender. The attributes and attribute values were selected in consultation with asylum policy experts from the Migration Policy Group, UNHCR, and the Swiss Refugee Council, and based on the detailed handbook that the Swiss State Secretariat of Migration provides for its asylum officers. This handbook specifies the topics on which the officers are required to elicit information during the asylum interviews. The goal was to ensure that we captured the most relevant characteristics that officials typically consider when deciding on asylum claims. In addition, we also included attributes the previous academic literature had identified as important for generating support for the admission of immigrants. Table S1 in the SI describes the full list of attributes and the possible values each attribute could take. Fully independent and uniform randomization was employed for all attributes. For the 2022 survey, we added a ``War'' level for the attribute ``Reason for migrating" given the salience of the Russia-Ukraine war. However, we also perform analyses while omitting profiles with this war level to enable a fully identical comparison with the 2016 design.

Reporting for specific materials, systems and methods

We require information from authors about some types of materials, experimental systems and methods used in many studies. Here, indicate whether each material, system or method listed is relevant to your study. If you are not sure if a list item applies to your research, read the appropriate section before selecting a response.

Materials & experimental systems

n/a	Involved in the study
☒	☐ Antibodies
☒	☐ Eukaryotic cell lines
☒	☐ Palaeontology and archaeology
☒	☐ Animals and other organisms
☒	☐ Clinical data
☒	☐ Dual use research of concern

Methods

n/a	Involved in the study
☒	☐ ChIP-seq
☒	☐ Flow cytometry
☒	☐ MRI-based neuroimaging
